# Light-Controlled Assembly and Disassembly of Covalent RNA–Protein Conjugates to Control RNA Base Editing

**DOI:** 10.3390/molecules31162924

**Published:** 2026-08-21

**Authors:** Alfred Hanswillemenke, Tim Stefan Berneiser, Marius Blackholm, Johann Kaiser, Anna Sofia Imrich, Karthika Devi Kiran Kumar, Hayase Hakariya, Thorsten Stafforst

**Affiliations:** 1 Interfaculty Institute of Biochemistry, University of Tübingen, Auf der Morgenstelle 15, 72076 Tübingen, Germany; 2 Gene and RNA Therapy Center (GRTC), Faculty of Medicine, University of Tübingen, 72076 Tübingen, Germany

**Keywords:** RNA editing, RNA targeting, Clip-tag, Snap-tag, photo-control

## Abstract

Self-labeling enzymes, like SNAP-, CLIP- and Halo-tag have found wide application in biology, biochemistry, materials science and bioengineering. For example, they have been applied with high versatility to rewrite genetic information inside the living cell by providing rationally programmable RNA-targeting strategies of protein-based effectors. Such approaches benefit from the engineering capability of the small molecule-based self-labeling moieties. Here, we further engineered that approach to control RNA-targeting by light. Specifically, we combined two orthogonal self-labeling enzymes for the recruitment of two distinct fusion proteins to a target RNA inside the living cell and achieved concurrent assembly and disassembly of distinct guide RNA–protein conjugates. We applied this to control RNA base editing and achieved a photo-induced swap of two distinct editing events. Overall, this work describes new ways for tool development, RNA imaging, and transcript engineering.

## 1. Introduction

Self-labeling enzymes (SLE) like the SNAP-tag [1], CLIP-tag [2] and Halo-tag [3] can be genetically fused to any protein of interest (POI) for their covalent attachment to surfaces, beads or any molecule of interest, including dyes, small molecules, enzymes, and nucleic acids [4]. SLEs typically have a size similar to GFP, can be well expressed in various genetic backgrounds, and have been used in vitro, in cellular and in vivo. The kinetics of the enzymatic single-turnover self-labeling reactions have been determined for various substrates and are in the range of 10^4^–10^6^ M^−1^s^−1^ [5]. Particularly mature is their application in super-resolution microscopy where POIs are covalently attached to highly engineered dyes [6]. Besides visualization, SLEs have been used to study the interaction of POIs [7,8,9], to enforce the contact of proteins [10], and to manipulate biological processes [4]. In our lab, we introduced the usage of SLEs for the engineering of RNA base editing tools [11,12,13,14]. RNA base editing is a strategy to rewrite genetic information at the RNA level in a highly programmable way. One example is site-directed A(denosine)-to-I(nosine) RNA editing [15]. As inosine is biochemically read as guanosine, protein isoforms with altered function can be created in a highly rational manner [16]. To target particular sites on specific mRNA transcripts, we engineered an artificial RNA editing system called SNAP-ADAR [11,12], which is based on the fusion of the self-labeling SNAP-tag [1] domain with the catalytic deaminase domain from the RNA editing enzyme ADAR (adenosine deaminase acting on RNA). The SNAP-tag catalyzes the transfer of a single guide RNA to itself, provided the guide RNA is modified with the *O*^6^-benzylguanine (BG) moiety. The covalently attached guide RNA then steers the SNAP-ADAR to its target (m)RNA in a programmable way following Watson–Crick base-pairing rules. The SNAP-ADAR approach applies a unique RNA-targeting mechanism, which enables the use of chemically densely modified guide RNAs [17]. These chemical modifications allow for controlling bystander editing in adenosine-rich targets [18]. Furthermore, the RNA-targeting mechanism works very well even under low expression of the artificial editing enzyme, markedly reducing global off-target effects [12].

An emerging trend is to control SLEs by light [19]. Light is a highly attractive trigger to study the dynamics of biochemical processes in cell culture and in transparent animals or their developing embryos [20]. Editing with SNAP-ADAR is fully dependent on the SNAP-tag-mediated, covalent assembly of guide RNA and SNAP-ADAR. This conjugation reaction can be exploited to include photo-control. Specifically, by installing an Npom (6-nitropiperonyloxymethyl) photo-caging group at the *N*7 of the BG moiety on the guide RNA, we were able to implement a photo-triggered on-switch of RNA editing activity [21]. Similarly, others have shown photo control of the SNAP-tag assembly reaction by light too [22]. Considerable efforts have also been made to control other RNA-guided proteins, e.g., the RNA-induced silencing complex (RISC) or the CRISPR-Cas system. For this, laboratories have developed caged nucleotides and amino acids to block functionally important sites temporarily [20,23,24], and control over genome editing with caged nucleotides has been described recently in vitro and in vivo [25,26,27].

Here, we now report on the photo-induced disassembly of self-labeling enzymes from their covalently attached guide RNAs. By combining two orthogonal self-labeling enzymes, SNAP- and CLIP-tag, we achieved simultaneous photo-control of the conjugation and the disassembly process of two different proteins, which was exploited to drive their protein localization inside the living cell. Furthermore, we used the photo-induced disassembly of guide RNA and SNAP-ADAR protein to switch off programmable RNA base editing. In combination with the established on-switch of conjugation [21], this was used to perturb the balance of two editing events with opposite effects on the endogenous *STAT1* transcript. Overall, this highlights the versatility of this RNA-guided RNA-targeting platform, which relies on the chemical engineering of the self-labeling moieties at the guide RNA component.

## 2. Results and Discussion

**Engineering a photo-cleavable self-labeling moiety to disassemble guide RNA–protein conjugates**

**Figure 1:**
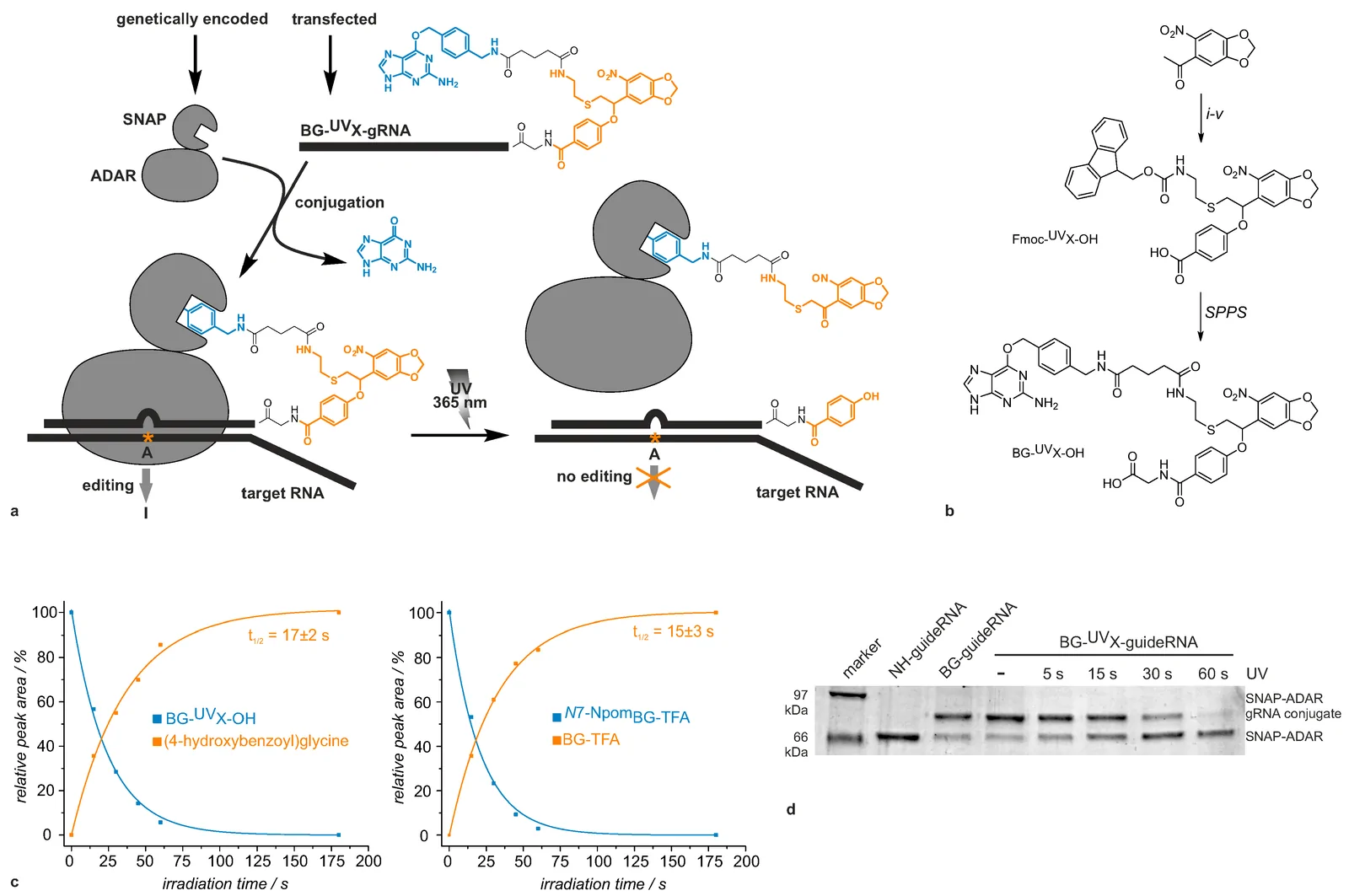
**Engineering of the light-induced disassembly of a guide RNA-effector protein conjugate.** (**a**) Schematic overview. The self-labeling BG moiety (blue) mediates the covalent assembly between guide RNA and SNAP-tagged effector protein, here exemplified by the editing enzyme ADAR, which leads to site-directed A-to-I RNA editing (orange asterisk). The BG-^UV^X-guide RNA contains a photosensitive moiety (orange), which can be used to photo-cleave the guide RNA-SNAP-ADAR conjugate, stopping targeted RNA editing abruptly. (**b**) Synthesis of the photo-cleavable, Fmoc-protected amino acid Fmoc-^UV^X-OH, and structure of the photo-cleavable linker BG-^UV^X-OH. (i) Br_2_, dioxane, 67%; (ii) (9H-Fluoren-9-yl)methyl (2-mercaptoethyl)carbamate, TEA, THF, 90%; (iii) NaBH_4_, MeOH, 98%; (iv) methyl paraben, PPh_3_, DIAD, THF, 28%; (v) LiOH, K_2_CO_3_, Fmoc-OSu, THF, ACN, H_2_O, 50%, yield SPPS: 71%. (**c**) Photo-cleavage kinetics of BG-^UV^X-OH and ^N7-Npom^BG-TFA monitored by HPLC. For more details, see Supporting Information, Figure S1. (**d**) In vitro assay of assembly and light-induced disassembly of the SNAP-ADAR-guide RNA conjugate. A BG-^UV^X-guide RNA was incubated with purified SNAP-ADAR3 for conjugation and treated with 365 nm light (transilluminator; light intensity 7.9 ± 0.2 mW/cm^2^) for various amounts of time (0–60 s) prior to SDS-PAGE analysis. As controls, the analog photo-insensitive BG-guide RNA and the conjugation-incompetent NH-guide RNA were loaded, defining the position of the free and guide RNA-conjugated SNAP-ADAR band, respectively. For more details on the experimental setup, see Supplementary Information.

To achieve photo-control over the disassembly of protein–RNA conjugates, we conceived a new photo-cleavable amino acid based on 6-nitropiperonyl alcohol [28]. In a simple five-step synthesis starting from 4′,5′-methylenedioxy-2′-nitroacetophenone, a photo-cleavable moiety was incorporated into the Fmoc-protected amino acid Fmoc-^UV^X-OH (Figure 1b). Through solid-phase peptide synthesis, Fmoc-^UV^X-OH was included in a photo-cleavable BG-linker (BG-^UV^X-OH) containing a terminal carboxyl group for subsequent attachment to a guide RNA. At this stage, the products and kinetics of photo-cleavage were determined (Figure 1c). We compared the cleavage of BG-^UV^X-OH side-by-side with the decaging of ^N7-Npom^BG-TFA [21]. We expected very similar key properties of both molecules (e.g., UV spectra, quantum yield). Indeed, BG-^UV^X-OH decomposed under formation of the respective nitrosoacetophenone and (4-hydroxybenzoyl)-glycine, as confirmed by HPLC-MS and comparison to reference samples (Supplementary Figure S1). The absorbance spectra > 340 nm of both photo-labile molecules overlapped almost perfectly, and both reacted upon irradiation (transilluminator, 365 nm at 7.9 ± 0.2 mW/cm^2^, PBS buffer pH 7, HPLC assay) with very similar kinetics, e.g., t_1/2_ = 17 ± 2 s (BG-^UV^X-OH) and 15 ± 3 s (^N7-Npom^BG-TFA). We then activated BG-^UV^X-OH as an N-hydroxysuccinimide ester, coupled it to a guide RNA carrying a 5′-terminal amino linker, and purified the product by PAGE following a recently described procedure [29]. The BG-^UV^X-guide RNA was incubated in vitro with purified SNAP-ADAR protein, and the assembly reaction was readily followed as a band shift in the SNAP-ADAR protein via SDS-PAGE, as previously described [21]. Within 60 s of irradiation (365 nm), the SNAP-ADAR-guide RNA conjugate almost entirely released SNAP-ADAR in a light-dose-dependent manner, demonstrating the photo-triggered disassembly of guide RNA and SNAP-ADAR protein (Figure 1d). Overall, the new photo-labile ^UV^X linker, when combined with a self-labeling moiety, enables the light-induced disassembly of a correspondingly tagged fusion protein from a guide RNA.

**The localization of two RNA-guided proteins can be controlled by light inside living cells**

**Figure 2:**
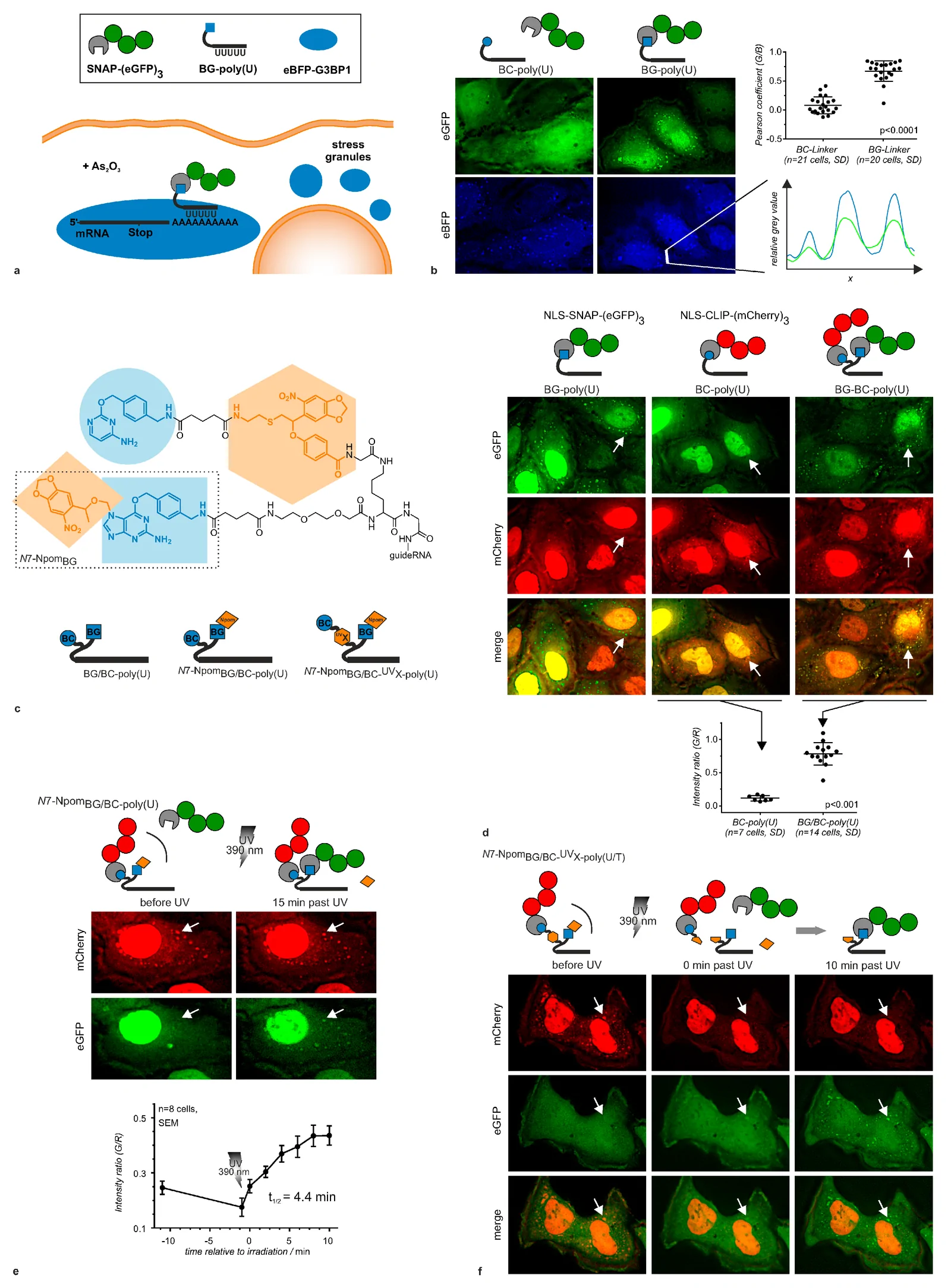
**Orthogonal recruitment of two different proteins under photo-control.** (**a**) Scheme of the staining of polyadenylated mRNAs in arsenite-induced stress granules with guide RNAs conjugated to fluorescent reporter proteins. (**b**) Recruitment of a BG-poly(U)-guide RNA into arsenite-induced stress granules (50 µM As_2_O_3_, 30 min) in a transgenic HeLa cell line, stably expressing a SNAP-(eGFP)_3_ reporter and a BFP-G3BP1 stress granule marker. Arsenite-induced stress granules appear as blue foci. The BG-poly(U)-guide RNA gives co-staining in the green channel, whereas the control BC-poly(U)-guide RNA does less, as indicated by the Pearson coefficient for the correlation of green and blue fluorescence at blue foci in N ≥ 20 cells. Statistical significance was calculated by an unpaired Welch’s *t*-test. (**c**) Highly functionalized linkers engineered to provide photo-control over the concurrent and orthogonal RNA-guided steering of two different proteins based on the self-labeling enzymes SNAP- and CLIP-tag. (**d**) Orthogonal recruitment of two proteins. Transgenic HeLa cells, stably co-expressing SNAP-(eGFP)_3_ and CLIP-(mCherry)_3_, were either transfected with a poly(U)-guide RNA carrying the BG, BC, or bifunctional BG/BC self-labeling moieties. The red channel monitors recruitment of mCherry; the green channel monitors recruitment of eGFP upon arsenite-induced stress granule formation. The intensity ratio (green/red) was measured at red foci in N = 7–14 cells. Statistical significance was calculated by an unpaired Welch’s *t*-test. (**e**) Light-triggered recruitment of a protein into stress granules. A bifunctional, photo-caged ^N7-Npom^BG/BC-poly(U)-guide RNA was transfected into a eGFP/mCherry transgenic HeLa reporter cell line. After arsenite treatment, the light-induced (390 nm, 100 ms, lumencor^®^ AuraII) recruitment of eGFP to mCherry-stained stress granules was monitored over time. Conjugation kinetics have been obtained by following the green/red ratios over 10 min. Error bars indicate standard error of the mean (SEM) for measurements in N = 8 cells. (**f**) Light-triggered swapping of two different proteins in stress granules. A bifunctional, photosensitive, and photo-caged ^N7-Npom^BG/BC-^UV^-X-poly(U)-guide RNA was transfected into the eGFP/mCherry transgenic HeLa reporter cell line. After arsenite treatment, stress granules appear red (mCherry) but not green (eGFP). After light treatment (390 nm, 100 ms, lumencor^®^ AuraII), mCherry fluorescence (red channel) disappears immediately and is replaced by eGFP fluorescence (green channel) over the course of several minutes. White arrows in panels c, e, f exemplarily point to foci where light-induced effects are clearly visible. For more detail on cell line creation and experimental setup, see the Supplementary Information.

To further characterize the achieved photo-control, we followed the photo-triggered disassembly reaction inside the living cell by fluorescence microscopy. First, we expressed a plasma membrane-bound SNAP-tag fusion protein [30] and labeled it with BG-^UV^X-Atto488, giving rise to intense green staining of the cell surface (Supplementary Figure S4). As expected, the Atto488 stain was efficiently removed with a short pulse (100 ms) of UV light (390 nm, lumencor^®^ AuraII, Beaverton, ON, USA), indicating that the engineered photo-labile linker is applicable to achieve fast light-induced disassembly of the fluorescence dye-protein conjugate on the living cell. To visualize the RNA-guided steering of proteins inside the living cell, we created a HeLa cell line stably expressing the green fluorescent reporter protein SNAP-(eGFP)_3_ and the blue fluorescent stress granule marker BFP-G3BP1 [31] (Figure 2a). We transfected a BG-poly(U)-guide RNA into these cells to recruit the SNAP-(eGFP)_3_ reporter protein in a guide RNA-dependent manner into arsenite-induced stress granules, which contain a bulk of polyadenylated mRNA [32]. Indeed, only in the presence of a BG-poly(U)-guide RNA a strong co-localization (Pearson coefficient 0.7) of the green (GFP) and blue (BFP) channel was detected (Figure 2b). A poly(U)-guide RNA carrying the *O*^2^-benzylcytosine (BC) moiety served as a negative control. Notably, the BC moiety could not induce a strong green-blue correlation (Pearson coefficient < 0.1). The BC moiety is the substrate for the self-labeling CLIP-tag [2]. Hence, the results indicated that the orthogonality between BG/SNAP- and BC/CLIP-tag might be sufficient to exploit them for the concurrent control of two different proteins inside the living cell. To further elucidate this, we created cell lines stably expressing the red fluorescent NLS-CLIP-(mCherry)_3_ reporter protein beside the green fluorescent NLS-SNAP-(eGFP)_3_ reporter protein and transfected them with poly(U)-guide RNAs either carrying the BG or BC self-labeling moiety (Figure 2d). Each guide RNA resulted in the respective coloring of arsenite-induced stress granules. The BG-guide RNA colored them predominantly green, the BC-guide RNA red. To better compare the fluorescence intensities of the red and green channels, we synthesized a bifunctional linker comprising one BG and one BC moiety, and attached it to the poly(U)-guide RNA (Figure 2c). The BG/BC-poly(U)-guide RNA consistently recruited both fluorescent proteins, presumably in a 1:1 stoichiometry, into the stress granules and achieved a stable green-to-red fluorescence intensity of 0.78. For comparison, the monofunctional BC-guide RNA recruited significantly less GFP, resulting in a green-to-red intensity ratio of only 0.11 (Figure 2d).

Next, we included photo-control of the guide RNA–protein conjugation/assembly reaction. By combining solid and liquid phase peptide chemistry, we synthesized the photo-caged, bifunctional linker ^N7-Npom^BG/BC, carrying an *N7*-Npom photo-caged BG moiety and a regular BC moiety, and attached it to the 5′-terminus of a poly(U)-guide RNA (Figure 2c). We had shown before that the installation of the *N*7-Npom protection group on *O*^6^-benzylguanin effectively inhibits the SNAP-tag-mediated conjugation reaction and enables an “on-switch” for the covalent conjugation of SNAP-tag fusion proteins, like the SNAP-ADAR effector, with guide RNAs under control of light [21]. Thus, as the BG moiety is photo-caged at the beginning, the respective ^N7-Npom^BG/BC-poly(U)-guide RNA stained arsenite-induced stress granules preferentially red before irradiation, resulting in an initial green-to-red ratio of ≈0.2. However, after a short pulse of 390 nm light (100 ms, lumencor^®^ AuraII), the green-to-red ratio increased to a final value of ≈0.45 after 10 min, demonstrating the light-induced, covalent co-recruitment of the free-floating SNAP-tagged GFP protein (Figure 2e). The kinetics of the SNAP-(eGFP)_3_ recruitment into the stress granules could be followed live by fluorescence microscopy. From the slope of the green-to-red ratio change over time, a half-life of ≈ 4.4 min was estimated, defining the time frame for photo-driven assembly reactions that can be realized with the SNAP-/CLIP-tag RNA-targeting approach. Finally, we combined the light-triggered control over assembly and disassembly of guide RNA–protein conjugates within one engineered guide RNA. For this, we synthesized the highly complex ^N7-Npom^BG/BC-^UV^X-Linker comprising the photo-caged BG moiety in combination with a photo-cleavable BC moiety, and attached it to a poly(U)-guide RNA. As expected, arsenite-induced stress granules turned red but not green after transfection of this guide RNA, indicating the preferred recruitment of CLIP-(mCherry)_3_. However, induced by a short UV light pulse (390 nm 100 ms, lumencor^®^ AuraII), the red fluorescence disappeared within seconds, followed by a slow recruitment of green fluorescence over several minutes, indicating the exchange of the RNA-targeted protein from CLIP-(mCherry)_3_ to SNAP-(eGFP)_3_ inside stress granules (Figure 2f).

While the microscopy supports the photo-induced recruitment and/or release of the SNAP- and CLIP-tagged reporter proteins to the respective poly(U)-guide RNA, the presence of poly(U)-guide RNAs in stress granules may not only be driven by their sequence-specific hybridization to poly(A) tails of mRNAs, but could also result from their unspecific deposition. To show that the modulation of sequence-specific RNA-targeting events is possible with our approach, we focused in the next step on switching RNA base editing on and off by the light-induced assembly and disassembly of guide RNA-SNAP-ADAR conjugates. RNA base editing is a highly programmable process that requires the sequence-specific formation of a guide RNA/target RNA duplex carrying a covalently attached SNAP-ADAR editing enzyme [12].

**Light-induced on- and off-switch of RNA editing with the SNAP-ADAR tool**

**Figure 3:**
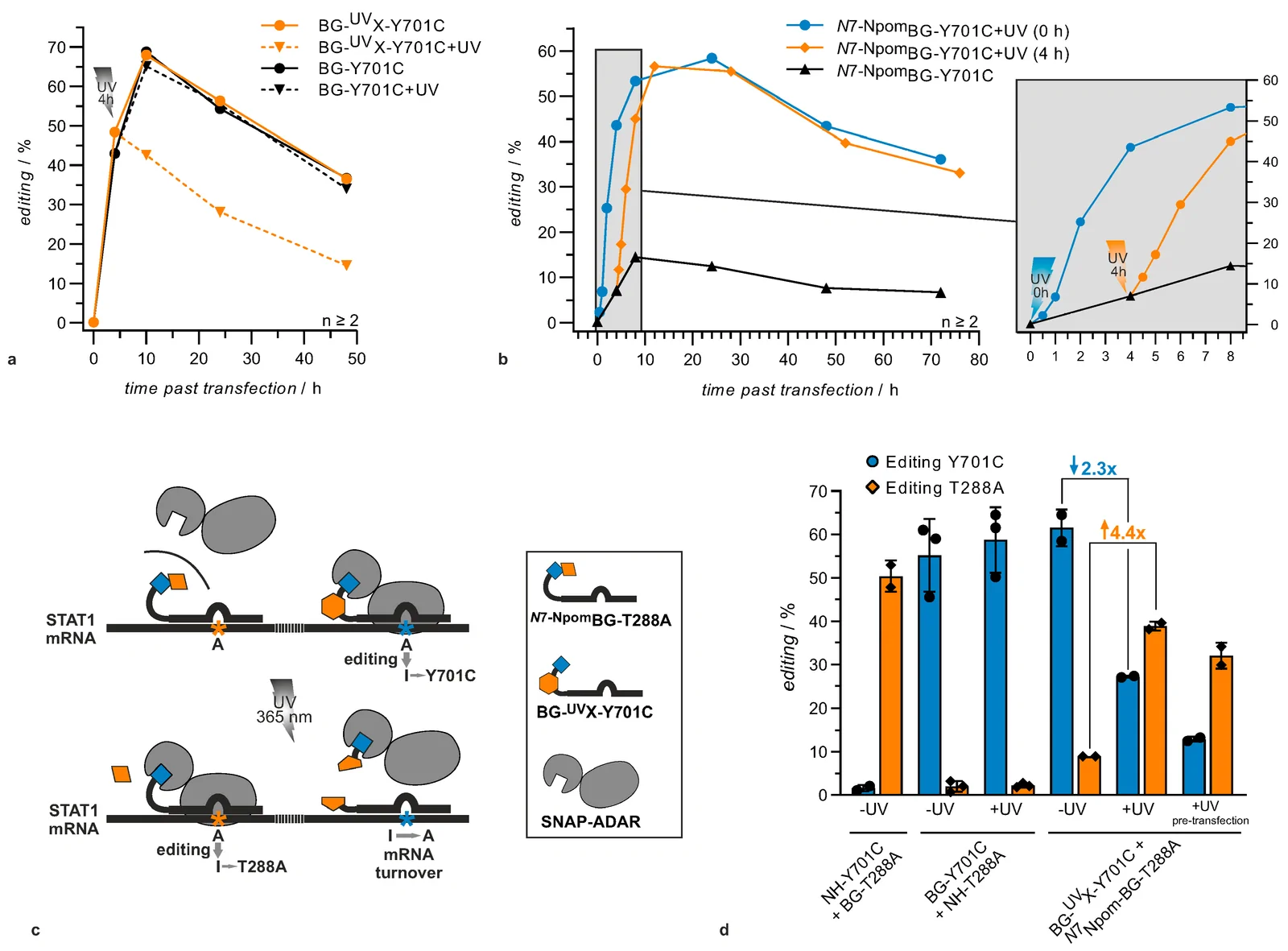
**Photo-control of RNA editing at two different sites in the endogenous STAT1 transcript.** (**a**) Photo off-switch of STAT1 editing. Shown is the editing yield at the endogenous STAT1 transcript (Y701C) over 72 h, with either a photo-cleavable BG-^UV^X- or a normal BG-guide RNA, in the absence or presence of a light trigger (+UV, 365 nm), which was applied for 3 min, 4 h after guide RNA transfection, as indicated. Shown are the mean values of N = 2–4 data points, for primary data see Supplementary Figure S2. (**b**) Photo on-switch of STAT1 editing. Shown is the editing yield at the endogenous STAT1 transcript (Y701C) over 48 h, in the presence of a photo-activatable ^N7-Npom^BG-guide RNA, absence or presence of a light trigger (+UV). Light was applied for 2 min (365 nm) either prior to guide RNA transfection (0 h) or 4 h afterwards (4 h). Shown are the mean values of N = 2–4 data points, for primary data, see Supplementary Figure S2. (**c**) Concept of photo-swapping between two editing events on endogenous STAT1. Two guide RNAs, one with a photo-activatable linker and one with a photo-cleavable linker, are co-transfected. Before irradiation, editing takes place at Y701 but not at T288. After irradiation, editing activity swaps, and T288 but not Y701 is edited. (**d**) Photo-swap is achieved by co-transfection of a photo-sensitive BG-^UV^X-guide RNA for Y701C and a photo-activatable ^N7-Npom^BG-guide RNA for T288A editing. Shown are controls with and without photo-trigger (365 nm, 3 min), control guide RNAs with and without self-labeling moiety (NH vs. BG), control guide RNAs with and without photo-control (BG vs. ^N7-Npom^BG vs. BG-^UV^X). For more details on the experimental setup, see Supplementary Information and Figure S2. The applied irradiation conditions did not show signs of cellular toxicity to SA1Q 293 Flp-In cells (Supplementary Figure S6).

An attractive application for the photo-induced disassembly of guide RNA–protein conjugates is site-directed RNA base editing with the SNAP-ADAR approach. Since the editing activity of the SNAP-ADAR tool is strictly dependent on its covalent conjugation to the guide RNA [12,21], we reasoned that introducing the photo-cleavable ^UV^X group between the guide RNA and the self-labeling BG moiety would enable a light-induced off-switch of RNA editing. We tested the concept on editing of the endogenous *STAT1* transcript in Flp-In 293T-REx cells stably expressing [12] the SNAP-ADAR1Q editase (Figure 3). STAT1 is an essential transcription factor in pathogen defense and cell homeostasis and is activated by phosphorylation of tyrosine 701 (Y701) in response to cytokines and growth factors [33]. After phosphorylation, STAT1 dimerizes, enters the nucleus, and acts as a transcription factor. We had shown before that Y701 in STAT1 can be defunctionalized to cysteine (C) by RNA editing [18], mimicking a naturally occurring genetic variant related to a dominant predisposition to mycobacterial diseases [34].

To follow the photo-induced switch off, we took a time profile of the editing reaction. With a standard BG-modified guide RNA, the maximum editing yield (~70%) was obtained 12 h post transfection; after 48 h, the editing yield was reduced by half. This effect was independent of irradiating the cells (Figure 3a). Exactly the same trend was observed for the photo-cleavable BG-^UV^X-guide RNA in the absence of light. However, when the cells were irradiated on a transilluminator (3 min, 365 nm) 4 h post transfection, the time profiles of the cleavable and non-cleavable guide RNA clearly segregated after irradiation. We speculate that the editing reaction was quickly reduced or stopped after photo-cleavage of guide RNA and SNAP-ADAR1Q. After irradiation the decline in editing yield over time followed the same kinetics as the two controls (Figure 3a). We speculate that this decline is governed by a slow replacement of the edited transcript prior to irradiation with newly synthesized, unedited RNA. Further evidence would be required to fully support this explanation.

We also tested the on-switch of RNA editing by transfecting a caged ^N7-Npom^BG-guide RNA with or without irradiation prior to transfection. As expected, the editing performance clearly differed, achieving editing yields >60% with the pre-irradiated guide RNA, while the untreated, caged guide RNA gave only minor yields <15% (Figure 3b). However, when the caged guide RNA was irradiated 4 h post transfection (2 min, 365 nm), editing levels increased abruptly to a maximum yield of ≈60%, and the profile matched that of the uncaged control guide RNA.

The transient manipulation of the *STAT1* transcript by RNA editing could become an attractive application for photo-triggered spatiotemporal control, as many heterozygous STAT1 mutations have been reported with distinct clinical phenotypes [34,35]. Particularly attractive could be the swapping between an editing event that writes a gain-of-function mutation and a contrary editing event that writes a loss-of-function mutation. To test this concept, we chose two editing events, Tyr701>Cys and Thr288>Ala. While the first mutation is reported as a loss-of-function mutation, the latter is a known gain-of-function mutation [35]. To achieve the swap, the cleavable BG-^UV^X-guide RNA for Y701C editing was co-transfected with a caged ^N7-Npom^BG-guide RNA for T288A editing. In the absence of light, an editing level of >50% was obtained at Y701 24 h post transfection, similar to that of the positive control (BG). In contrast, the editing level at T288 stayed <15%. However, when the cells were irradiated (3 min, 365 nm) 4 h post transfection, the editing levels at Y701 and T288 had swapped 20 h later (Figure 3d), demonstrating the feasibility of the concept. However, the light-driven off-switch was not complete. In a control experiment (Supplementary Figure S3) we simulated a disassembly of 97% of the guide RNA-SNAP-ADAR conjugate and found a residual editing yield of 10% showing that the notably high editing efficiency of the SNAP-ADAR tool is likely causing the residual editing yield seen in Figure 3d.

## 3. Materials and Methods

*Photodissociation kinetics of BG-^UV^X-OH.* To determine the decaging efficiency εΦ, the decay of BG-^UV^X-OH was compared to the decaging of the previously characterized ^N7-Npom^BG-TFA as described before [21]. The latter was chosen because of the similar absorbance properties of their photo-cleavable moieties (see Supplementary Figure S1a). For both substances the extinction coefficient was estimated to be ε365 nm = 4.3 mM^−1^cm^−1^ as determined for Npom-OH. Stock solutions of BG-^UV^X-OH and ^N7-Npom^BG-TFA were prepared in DMSO and diluted in PBS (137 mM NaCl, 2.7 mM KCl, 12 mM K_2_HPO_4_, 12 mM KH_2_PO_4_, pH = 7.4) to a final concentration of 10 μM (DMSO < 0.02%). Decaging was performed in PCR tubes by irradiation with 365 nm light on a UV transilluminator (UVP TFL-40V, 25 W power, intensity high, giving 7.9 ± 0.2 mW/cm^2^ on the sample) for the indicated amount of time at room temperature. Samples were covered with aluminum foil and stored at −20 °C until they were applied to analytical HPLC with UV detection at 280 nm and 365 nm (see Supplementary Figure S1d,f). Decomposition of ^N7-Npom^BG-TFA (D) results in two clean products, BG-TFA (F) which shows high absorption at 280 nm but no absorption at 365 nm and the released caging group (E) that shows high absorbance at 365 nm (see Supplementary Figure S1e,f). The photo-cleavable linker BG-^UV^X-OH (A) decomposed to several peaks on the HPLC (see Supplementary Figure S1c,d), one of which can be assigned to be (4-hydroxybenzoyl)glycine (C) by LC-MS and comparison to the reference substance synthesized (spectra below). (4-Hydroxybenzoyl)glycine is the cleavage product that remains on a guideRNA after irradiation in editing experiments and therefore demonstration of its timely release is most relevant for this study. Another peak could be assigned to be the nitroso acetophenone product (B) containing the benzyl guanine by LC-MS which shows absorption at 365 nm (spectra below). The peaks were integrated with the Shimadzu VP-Class 7.4 SP1 software.

The peak area of the cleavage products was plotted against irradiation time (see Figure 1c) and by logarithmic fit the half-time was determined to be t_1/2_ = 17 ± 2 s for (4-hydroxybenzoyl)glycine and t_1/2_ = 15 ± 3 s for BG-TFA. With the quantum yield of the reference substance (^N7-Npom^BG-TFA Φ ≈ 0.5), the quantum yield of BG-^UV^X-OH can be calculated to be Φ ≈ 0.4 according to following formula:

ΦBG−X UV−OH= ΦReference×t1/2 referencet1/2 BG−X UV−OH×εreferenceεBG−X UV−OH=0.41

*Chemical synthesis.* If not stated otherwise, all substrates and reagents required for synthesis and biochemical studies were purchased from commercial providers and used without further purification. *O*^6^-(4-aminomethyl-benzyl)guanine (BG-NH_2_) was synthesized as described in the literature [1]. 2-(4-(Aminomethyl)-benzyloxy)-4-aminopyrimidine (BC-NH2) was prepared from commercially available methyl-4-(aminomethyl) benzoate hydrochloride according to literature [2,36,37]. We recently described a detailed synthesis of ^N7-Npom^BG-TFA and ^N7-Npom^BG-NH2 [29].

All column chromatographic purifications were carried out on self-packed columns of silica gel (0.04–0.063 mm/230–240 mesh). Thin-layer chromatography (TLC) was performed on silica gel sheets (60 F254, 0.2 mm, 5 × 10 cm, Merck, Darmstadt, Germany) and visualized under UV light (254 nm). All analytical and preparative HPLC runs were performed on a Shimadzu (Kyoto, Japan) system (SCL-10A VP, SPD-20AV, LC-20AT) running with 0.1% TFA in water (eluent A) and 0.1% TFA in acetonitrile/water (9:1, eluent B). Analytical HPLC was performed using an EC 125/4 Nucleodur C18 column by Machery + Nagel (Düren, Germany) with a linear gradient from 5% eluent B (starting after 1 min) to 95% eluent B (ending after 25 min). Preparative HPLC was performed using a VP 250/10 Nucleodur C18 column by Machery + Nagel. High resolution mass spectrometry was performed on a maXis4G ESI-TOF-MS by Bruker Daltonics (Bremen, Germany). LC-MS analyses were conducted on a LC-MS2020 (Shimadzu, Kyoto, Japan) with a Kinetex C18 column (Phenomonex, Torrance, CA, USA). A total of 0.1% formid acid in water (eluent A) and 0.1% formic acid in acetonitrile/water (4:1, eluent B) were used as eluents. A linear gradient from 5% eluent B (starting after 1 min) to 95% eluent B (ending after 11 min) was used. NMR spectra were recorded on a Bruker ARX 250 spectrometer at 250 MHz for ^1^H spectra and 63 MHz for ^13^C spectra or a Bruker Avance 400 spectrometer at 400 MHz for ^1^H spectra and 101 MHz for ^13^C spectra. 2D-NMR spectra were recorded with a Bruker Avance 400 at 400 MHz/101 MHz.

The photo-cleavable amino acid Fmoc-^UV^X-OH was synthesized in a five-step synthesis starting with the bromination of 4′,5′-methylenedioxy-2′-nitroacetophenone which was carried out following the description of Pendrak et al. [38] The brominated product was reacted with Fmoc-protected cysteamine which was synthesized following the protocol of Miura et al. [39] The detailed protocol, including characterization of all compounds is given in the Supplementary Information.

*Solid-phase peptide synthesis (SPPS).* The general SPPS setup used here was recently described in detail [29]. In short manual synthesis was performed in polypropylene/polyethylene syringes equipped with a polyether sulfone frit. Incubation was carried out on a VIBRAX VXR basic (IKA) shaker (1500 rpm) at room temperature. Unless indicated otherwise the resin was washed after each coupling, capping or deprotection step with NMP/DCM 1:1 (3 times 5 mL), DCM (3 times 5 mL) and NMP (3 times 5 mL). If not indicated otherwise all amino acids were pre-activated with HBTU/HOBt for 5–15 min at room temperature before being added to the resin. BG-OH and ^N7-Npom^BG-OH were synthesized as previously described [29]. The detailed protocol, including characterization of all compounds is given in the Supplementary Information.

*guideRNA synthesis.* NH-Stop-66 guideRNA was obtained from Eurofins in HPLC-purified quality carrying a 5′-C6-aminolinker. NH-Y701C and NH-T288A were obtained from Biospring (Frankfurt, Germany) in HPLC-purified quality carrying a 5′-C6-aminolinker. O-Methyl-modified poly(U) was obtained from Sigma Aldrich in desalted quality carrying a 5′-C6-aminolinker and OMe/LNA modified poly(U) was obtained from Eurogentec (Seraing, Belgium) in desalted quality carrying a 5′-C6-aminolinker and a 3′-C7-aminolinker. NH-Y701C and NH-T288A guideRNAs were solved in RNase-free water to a concentration of 6 μg/μL. NH-Stop66 and NH-poly(U) were precipitated with 0.1 volumes of 3 M NaCl and 3 volumes of 100% EtOH, washed with 70% EtOH and dissolved in RNase-free water (6 μg/μL) prior to coupling. We recently described the guideRNA synthesis in detail [29]. In short the respective linker (BG-OH, BC-OH, ^N7-Npom^BG-OH, BG-^UV^X-OH, BC-^UV^X-OH, BG/BC-OH, ^N7-Npom^BG/BC-OH or ^N7-Npom^BG/BC-^UV^X-OH) was activated with DIC, NHS and DIPEA in DMSO according to the protocol for the synthesis of bifunctional linker described above. For all linker containing photo-sensitive moieties the reaction was performed protected from light. The resulting NHS ester was then reacted with the corresponding NH_2_-guideRNA in DMSO/H2O/DIPEA 66:33:1 and purified on a 20% UREA-PAGE. For all linker containing photo-sensitive moieties, the UREA-PAGE was performed in the dark. While the main part of the gel was protected with aluminum foil one lane containing ca. In total, 10% of the crude guideRNA was analyzed on a TLC plate under 254 nm UV light. The migration of NH_2_-guideRNA and modified gRNA were noted, and the analyzed lane was discarded. The region corresponding to migration of the modified gRNA was cut out from the remaining lanes and was transferred to an amber reaction tube. guideRNAs were extracted from the gel slices by shaking overnight at 4 °C with nuclease free water and precipitated with 0.1 volumes sodium acetate (NaOAc, 3 M) and 3 volumes of EtOH 100% (incubation at −20 °C for >4 h). After centrifugation (17,000× *g*, −4 °C, >45 min) the guideRNA pellet was washed with 70% EtOH and dissolved in nuclease free water. Concentrations were determined photometrically by absorption at 260 nm. The extinction coefficients were estimated from the ε260 nm provided from the commercial supplier and the sum of the following moieties incorporated into the terminal modification: BG (ε260 nm~2.5 mM^−1^cm^−1^), BC (ε260 nm~4.2 mM^−1^cm^−1^), ^N7-Npom^BG (ε260 nm~6.5 mM^−1^cm^−1^) and ^UV^X (ε260 nm~23 mM^−1^cm^−1^).

The synthesis of BG-guideRNAs and ^N7-Npom^BG-guideRNAs has been well established in previous publications [21,29]. In our experience the same protocol also achieves good yields of BG-UVX and bifunctional guideRNAs. Further data and full sequences of guide RNAs are given in the Supplementary Information.

*ADAR SDS-PAGE Shift Assay*. Expression and purification of SNAP-ADAR3 was performed as previously described [40]. SNAP-ADAR 3 (0.5 µM) and guideRNAs (2 µM) were diluted in 8 µL reaction buffer (10 mM Tris-HCl, 100 mM NaCl, 5% glycerol at pH 8.0). The reaction was carried out in PCR tubes which were wrapped in aluminum foil for light protection. After incubating for 30 min at 30 °C, the samples were irradiated on an UV table (UVP high performance UV transilluminator, 365 nm). In total, 4 µL of 4× SDS loading buffer (SDS (8% *w*/*v*), glycerol (40% *v*/*v*) and bromophenol blue (0.015% *w*/*v*) was added, and the samples were applied to an SDS-PAGE gel electrophoresis (4% stacking gel, 12% loading gel, gel was run in the dark). GE Healthcare (Chicago, IL, USA) LMW protein marker was applied as size marker and the proteins were visualized by Coomassie staining. The staining solution was composed of Coomassie Brilliant Blue G-250 (0.02% *w*/*v*), Al_2_(SO_4_)_3_ (5% *w*/*v*), EtOH (10% *v*/*v*) and phosphoric acid (2% *v*/*v*).

*Cloning.* The full sequence of all previously unpublished plasmids used in this study can be found in the appendix of the Supplementary Information and plasmid maps are found below. *eBFP-G3BP1* was created by amplifying the coding sequence of *G3BP1* from the cDNA of Flp-In 293T-REx cells and fusing it with *eBFP2* (obtained from Addgene, Watertown, NY, USA, Cat.No.54595) by primer extension and restriction cloning. *SNAP-(eGFP)_3_* and *NLS-SNAP-(eGFP)_3_* were created by primer extension PCR of the mammalian cell optimized *SNAPf-tag* (New England Biolabs, Ipswich, MA, USA) and fusion to three copies of *eGFP* by restriction cloning. *NLS-CLIP-(mCherry)_3_* was created by primer extension PCR of the *CLIPf* tag (New England Biolabs) and fusion to three copies of the red fluorescent protein *mCherry* by restriction cloning.

For generating stable HeLa cell lines expressing two transgenes we used the PiggyBac transposase approach [41]. The carrier plasmid PB-CA was obtained from Addgene (Watertown, NY, USA, Cat.No.20960) [42] and the CA resistance was exchanged with a puromycin resistance gene (*PuroR*). The CMV promoter was replaced by an expression cassette containing a bidirectional tetracycline responsive promoter [43]. Subcloning of *eBFP-G3BP1* and *SNAP-(eGFP)_3_* followed by a SV40 polyadenylation signal resulted in PiggyBac-Puro_SNAP-(eGFP)_3__eBFP-G3BP1 (used in Figure 3b). Subcloning of *NLS-CLIP-(mCherry)_3_* and *NLS-SNAP-(eGFP)_3_* followed by a SV40 polyadenylation signal resulted in PiggyBac-Puro_NLS-SNAP-(eGFP)_3__NLS-CLIP-(mCherry)_3_. Subcloning of *NLS-CLIP-(mCherry)_3_* and *SNAP-(eGFP)_3_* followed by a SV40 polyadenylation signal resulted in PiggyBac-Puro_SNAP-(eGFP)_3__NLS-CLIP-(mCherry)_3_.

For generating a stable, doxycycline-inducible HeLa cell line expressing the SNAP and CLIP tag on the plasma membrane surface, we used the previously described all-in-one, Tet-On 3G inducible PiggyBac plasmid XLone [44]. XLone-GFP was obtained from Addgene (Cat.No. 96930) and the ORF of the blasticidin resistance gene (*Bsd*) was replaced with a puromycin resistance gene (*PuroR*) by restriction cloning. Afterwards *GFP* was entirely replaced by the SNAP-CLIP-display fusion protein which was a kind gift from Prof. Dr. Kai Johnsson´s laboratory (Max Planck Institute for Medical Research, Heidelberg, Germany) [30], resulting in the XLone-Puro_SNAP-CLIP-display plasmid. A mammalian codon-optimized PiggyBac-transposase (*mPB*) gene [41] was subcloned into the commercial pcDNA3.1 backbone resulting in the pcDNA3.1_mPB plasmid.

*Cell culture.* If not stated otherwise, cells were cultivated in Dulbecco’s Modified Eagle Medium (DMEM, Thermo Fisher Scientific) supplemented with 10% fetal bovine serum (FBS, Thermo Fisher Scientific) at 37 °C with 5% CO_2_ in a water-saturated steam atmosphere. For subcultivation and seeding, cells were washed with phosphate-buffered saline (PBS), detached with 0.25% trypsin/EDTA (Sigma Aldrich, St. Louis, MO, USA), and resuspended in fresh DMEM/FBS. Subcultivation was performed every 3–5 days. For editing experiments Flp-In 293T-REx cells, stably expressing SNAP-ADAR1Q were used. The creation and subcultivation of this cell line have been previously described [12,45].

Creation of HeLa PiggyBac cells. We used the previously described mPB PiggyBac transposase for generating stable transgenic HeLa cell lines [41]. For this, 1 × 10^5^ HeLa cells (DSMZ, Braunschweig, Germany, no: ACC 57) were seeded in 0.5 mL DMEM/FBS in a 24-well plate. After 24 h, medium was replaced with 0.4 mL fresh DMEM/FBS, and 800 ng of the respective PiggyBac carrier plasmid and 200 ng pcDNA3.1_mPB were transfected with 3 µL Fugene6 (Promega, Madison, WI, USA) in 100 µL OptiMEM according to the manual. After 24 h, the cells were transferred to two wells of a 6 well plate. Forty-eight h later, the medium was replaced with DMEM/10% FBS/5 µg/mL puromycin. After 12 days of selection, the medium was replaced by DMEM/FBS. Cells were used without monoclonal selection and subsequently cultured in DMEM/FBS.

*Microscopy.* Microscopy was performed on a Nikon (Tokyo, Japan) Elipse Ti2-E inverted fluorescent microscope equipped with a Photometrics^®^ Prime 95B camera and a lumencor^®^ AuraII light engine. All pictures were recorded using a 60X oil objective (numerical aperture 1.4) and Olympus IMMOIL-F30CC immersion oil. The excitation wavelengths and corresponding filter sets used to record each channel are specified in the following table. Typically, a z-stack covering 6 µm (0.2 µm steps) was recorded. The images were deconvoluted using Nikon NIS-Elements AR v4.30.00 software and a single layer (z resolution ~0.6 µM) is displayed. Assignment of lookup tables, contrast settings and cropping were performed in FIJI ImageJ v1.53c [46]. The same acquisition settings were chosen for every channel within one subfigure.

Quantification of fluorescent signal in stress granules. Quantification of the red and green signal in stress granules was performed in Nikon NIS-Elements software using a maximum projection in z. The experiments were performed with the NLS-SNAP-(eGFP)_3_+NLS-CLIP-(mCherry)_3_ cell line, due to the low background signal in the cytoplasm. Since the NLS-CLIP-(mCherry)_3_ signal served as reference, we manually chose cells with clearly visible stress granules in the red channel for quantification. The cytoplasm was defined as region of interest (ROI) by manually outlining the cells. The nucleus was detected in the red channel and excluded using the bright spot detection function of Nikon NIS-Elements software (minimal ⌀ set to 15 µM). Background was subtracted using rolling ball correction (ball size = 1.5 µM) and stress granules were detected using bright spot detection in NIS-Elements software (settings: growing 400–600). The mean intensity of eGFP and mCherry signal for each spot was measured and the ratio calculated. For each cell the mean of the ratios of all quantified granules is shown (Figure 2d,e). The values for all individual granules quantified are shown below in Figure S5, right panel. Significance was calculated in GraphPad Prism 8 (version 8.4.3) using an unpaired Welch’s *t* test.

The same quantification could not be applied to the correlation of SNAP-(eGFP)_3_ and G3BP1 since the expression levels of eBFP-G3BP1 varied stronger and the transfection efficiency influenced the amount of SNAP-(eGFP)_3_ in the granules relative to eBFP-G3BP1. Instead, the local correlation of eBFP-G3BP1 and SNAP-(eGFP)_3_ was determined (Figure 2b). Since the eBFP-G3BP1 signal served as reference, we manually chose cells with clearly visible stress granules in the blue channel for quantification. The cells were manually outlined and defined as region of interest (ROI). Stress granules were selected using bright spot detection in NIS-Elements software (1–1.2 µM size) and a circular selection (⌀ 15px) around each granule was analyzed. Pearson correlation was determined for the eGFP and G3BP1 channel within each of the selections (Figure S5, left panel) and averaged for all stress granules of one cell (Figure 2b). Significance was calculated in GraphPad Prism 8 (version 8.4.3) using an unpaired Welch’s *t* test.

## 4. Conclusions

In summary, we demonstrated the high versatility of using self-labeling enzymes like SNAP [1] and CLIP-tag [2] for targeting proteins to RNAs of interest. Self-labeling enzymes have unique and advantageous properties compared to competing strategies that rely on Cas9 [47] or Cas13 [15,48]. First, SNAP- and CLIP-tags are small proteins (28 kDa), engineered from a human *O*^6^-methylguanine-DNA-methyltransferase, and are ready for C- and N-terminal tagging. They are readily cloned and stably integrated into cell lines, as demonstrated here. Second, self-labeling enzymes apply a covalent mechanism for the assembly of functional RNA–protein conjugates [11]. Importantly, different self-labeling enzymes can be combined for the orthogonal and concurrent recruitment of two different proteins in a defined stoichiometry inside the living cell, as we have demonstrated here. Furthermore, the unique covalent assembly mechanism can be manipulated and controlled inside the living cell. Specifically, we now demonstrate both photo-induced assembly and disassembly of guide RNA–protein conjugates. This was used to recruit poly(U)-RNA-conjugated proteins to polyadenylated bulk mRNAs in stress granules and release them upon irradiation. While this process might not be fully hybridization-dependent, we further applied this approach to switch strictly hybridization-dependent RNA base editing activity off by the photo-induced cleavage of guide RNA-SNAP-ADAR conjugates. RNA base editing is highly programmable by Watson–Crick base pairing as it strictly depends on the formation of a guide RNA/target mRNA substrate duplex. From experimental data provided here (Supplementary Figure S3) and elsewhere [18,21], we expect that cleaving the covalent bond between the SNAP-ADAR deaminase and its guide RNA results in a very quick loss of editing activity, by either dissociation of the SNAP-ADAR from its guide RNA/mRNA substrate duplex or by dissociation of the substrate duplex, and the apparently slow decline of the editing yield likely reflects the RNA turnover kinetics. Importantly, after photo-induced disassembly of the guide RNA-SNAP-ADAR conjugate, the released guide RNA and SNAP-ADAR protein are both conjugation-incompetent and cannot engage in the formation of a next round of editing-competent conjugates. However, their presence will also not interfere strongly with the editing activity of the functional conjugate (see Supplementary Figure S3). Finally, we used the approach to swap between two different RNA editing events on the endogenous *STAT1* transcript by photo-induction, highlighting novel opportunities for the targeted engineering of the transcriptome [18]. With a pair of orthogonal caging groups, a sequential photo-control would also be conceivable in the future [24,25]. Our approach complements the available classical optochemical approaches [49,50], where either a nucleo base or an essential amino acid in a protein would be caged with a photo-cleavable moiety in order to engineer photo-control [51]. Here, we show that a biochemical activity, like RNA base editing, can be controlled by light-induced disassembly of the engineered guide RNA/effector protein conjugate. An on/off switch of the RNA/protein assembly process has been achieved in the past with optogenetic approaches too [52]. While optogenetic approaches typically satisfy the need for easy-to-use, genetically fully encoded approaches [53], optochemical systems are more difficult to use but ensure more flexibility in the design [49]. Overall, our strategy may enable and inspire new applications to photo-control biochemical processes [20], e.g., it might become useful to transiently manipulate specific transcripts with sufficient spatiotemporal control in quickly developing embryos, like those of zebrafish or *Platynereis dumerilii* [21].

## Data Availability

The original contributions presented in this study are included in the article/Supplementary Materials. Further inquiries can be directed to the corresponding author.

## Supplementary Materials

The following supporting information can be downloaded at: https://www.mdpi.com/article/10.3390/molecules31162924/s1, Figure S1: Photo-dissociation kinetics of BG-^UV^X-OH, Figure S2: Editing yields of independent biological replicates of photo-controlled RNA editing at the Y701C site in the endogenous STAT1 transcript, Figure S3: Correlation of editing and the amount of functional, conjugation-competent BG-guideRNA, Figure S4: Characterization of the light-triggered cleavage reaction, Figure S5: Quantification of orthogonal recruitment of two different proteins to stress granules, Figure S6: The applied amount UV light did not lead to visible toxicity, further details on photodissociation kinetics, chemical syntheses, characterization of chemical compounds, solid phase peptide syntheses, cloning, cell culture, RNA editing experiments, microscopy, full sequences of all plasmids.
