# Supplementary material for: Light-Controlled Assembly and Disassembly of Covalent RNA–Protein Conjugates to Control RNA Base Editing
Source: Molecules. 2026 Aug 21;31(16):2924. doi: 10.3390/molecules31162924 (PMC13515618; doi:10.3390/molecules31162924)
Supplement: Supplementary file 1 [file molecules-31-02924-s001.zip › molecules-4275037-supplementary.pdf]

Supplementary Information

Content:

**Extended data**	**2**
Supporting Figures S1-S6	2
Photodissociation kinetics of BG-^{UV}X-OH	8
**Chemical synthesis**	**12**
Synthesis of Fmoc-^{UV}X-OH	12
Solid-phase peptide synthesis (SPPS)	27
Synthesis of bifunctional linker	33
Synthesis of BG-Atto488 and BG-^{UV}X-Atto488	36
guideRNA synthesis	37
**SNAP-ADAR SDS-PAGE shift Assay**	**39**
**Cloning**	**40**
**Cell culture**	**43**
Creation of HeLa PiggyBac cells	43
RNA editing experiments	45
Stress granule imaging experiments	45
Surface labeling experiments	46
Microscopy and image processing	46
**Quantification of fluorescent signal in stress granules**	**47**
**Appendix**	**49**
Unedited image of SDS-PAGE, Fig. 1d	49
Full plasmid sequences	50
Exemplary Sanger sequencing traces	65

Extended data

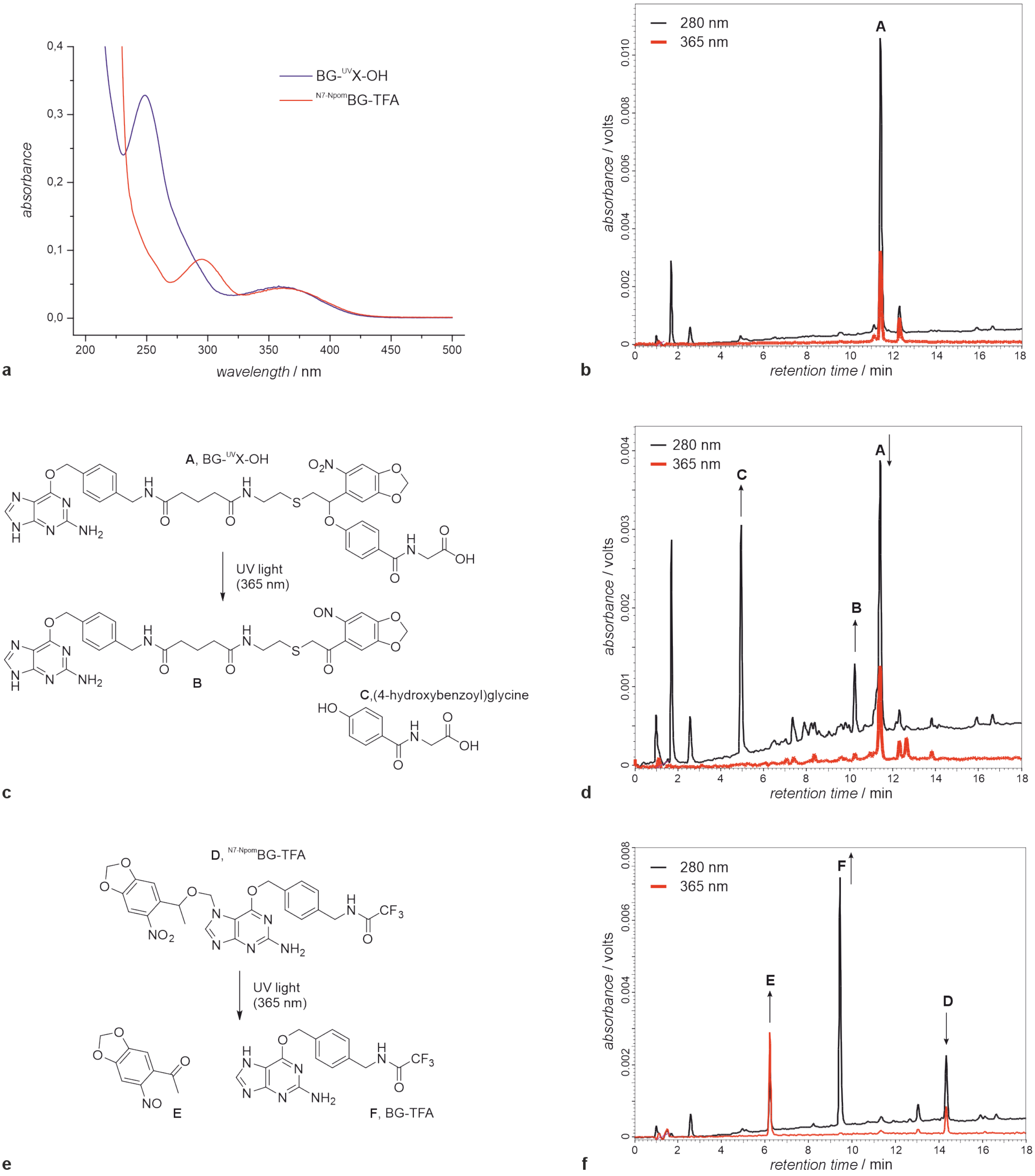

Figure S1. **Photo-dissociation kinetics of BG-^{UV}X-OH.** ***a)*** *Absorption spectra of BG-^{UV}X-OH and $^{N7\text{-}Npom}$BG-TFA.* ***b)*** *Analytical HPLC of BG-^{UV}X-OH before irradiation.* ***c)*** *Expected photo-dissociation products (**B+C**) of BG-^{UV}X-OH (**A**).* ***d)*** *Analytical HPLC of BG-^{UV}X-OH after partial photo-dissociation. Peaks are labeled according to Fig.S1c* ***e)*** *Reference substance $^{N7\text{-}Npom}$BG-TFA (**D**) and the expected decaging products (**E+F**)* ***f)*** *Analytical HPLC of $^{N7\text{-}Npom}$BG-TFA after partial decaging. Peaks are labeled according to Fig.S1e.*

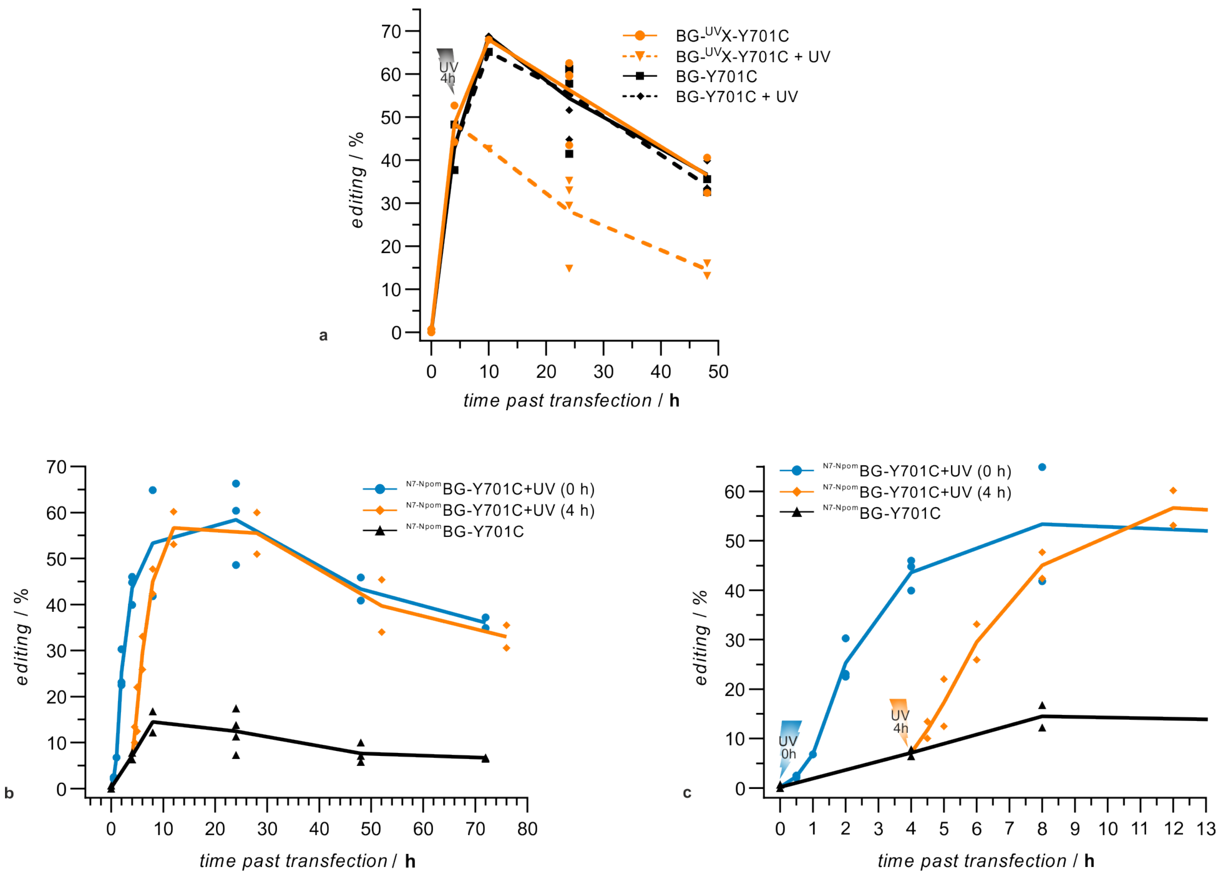

Figure S2. **Editing yields of independent biological replicates of photo-controlled RNA editing at the Y701C site in the endogenous STAT1 transcript.** ***a)*** *and* ***b)*** *Photo off-switch of STAT1 editing as shown in Figure 2a. Shown is the editing yield of independent biological experiments, with either a photo-cleavable BG-^{UV}X- or a normal BG-guideRNA, in the absence or presence of a light trigger (+UV, 365 nm), which was applied for 3 min, 4 h after guideRNA transfection, as indicated.* ***c)*** *Photo on-switch of STAT1 editing as shown in Figure 2b. Shown is the editing yield of independent biological experiments over 72 h, in the presence of a photo-activatable $^{N7\text{-}Npom}$BG-guideRNA, absence or presence of a light trigger (+UV). The light trigger was applied for 2 min (365 nm) either prior to guideRNA transfection (0 h) or 4 h afterwards (4 h).*

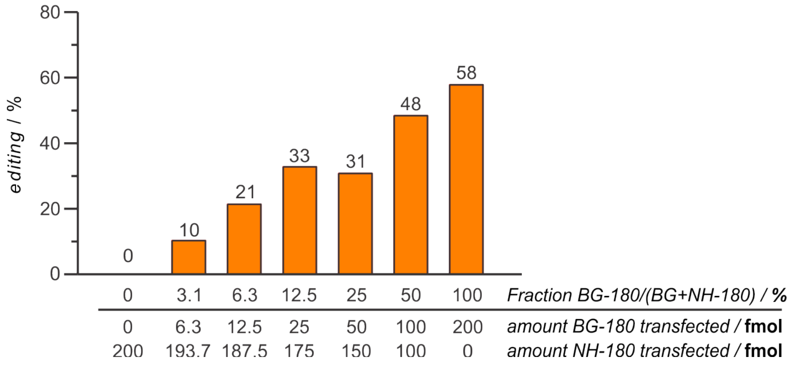

Figure S3. **Correlation of editing and the amount of functional, conjugation-competent BG-guideRNA.** *BG-guideRNA (conjugation-competent) and NH-guideRNA (nonfunctional control, conjugation-incompetent) targeting the Y701C site within the endogenous STAT1 transcript were mixed at different ratios and transfected into SNAP-ADAR-expressing Hek293T cells. Shown is the editing yield obtained upon transfection of 200 fmol total guideRNA containing 3.1% to 100% of conjugation-competent BG-guideRNA. Notably, editing does not show a linear correlation with the fraction of BG-guideRNA, but already 6.3 fmol BG-guideRNA were able to induce significant editing (10%) even in the presence of 30fold excess NH-guideRNA. The setup mimics a situation where a tiny fraction of* $^{N7\text{-}Npom}$*BG-guideRNA is decaged by ambient light or dark hydrolysis in an on-switch editing experiment, or it mimics the result of incomplete cleavage of BG-*^{UV}*X-guideRNA in an off-switch editing experiment. In both situations, tiny amounts of conjugation-competent guideRNA can account for the observed premature editing (on-switch) or residual editing (off-switch) due to the high efficiency of the SNAP-ADAR enzyme for targeted editing.*

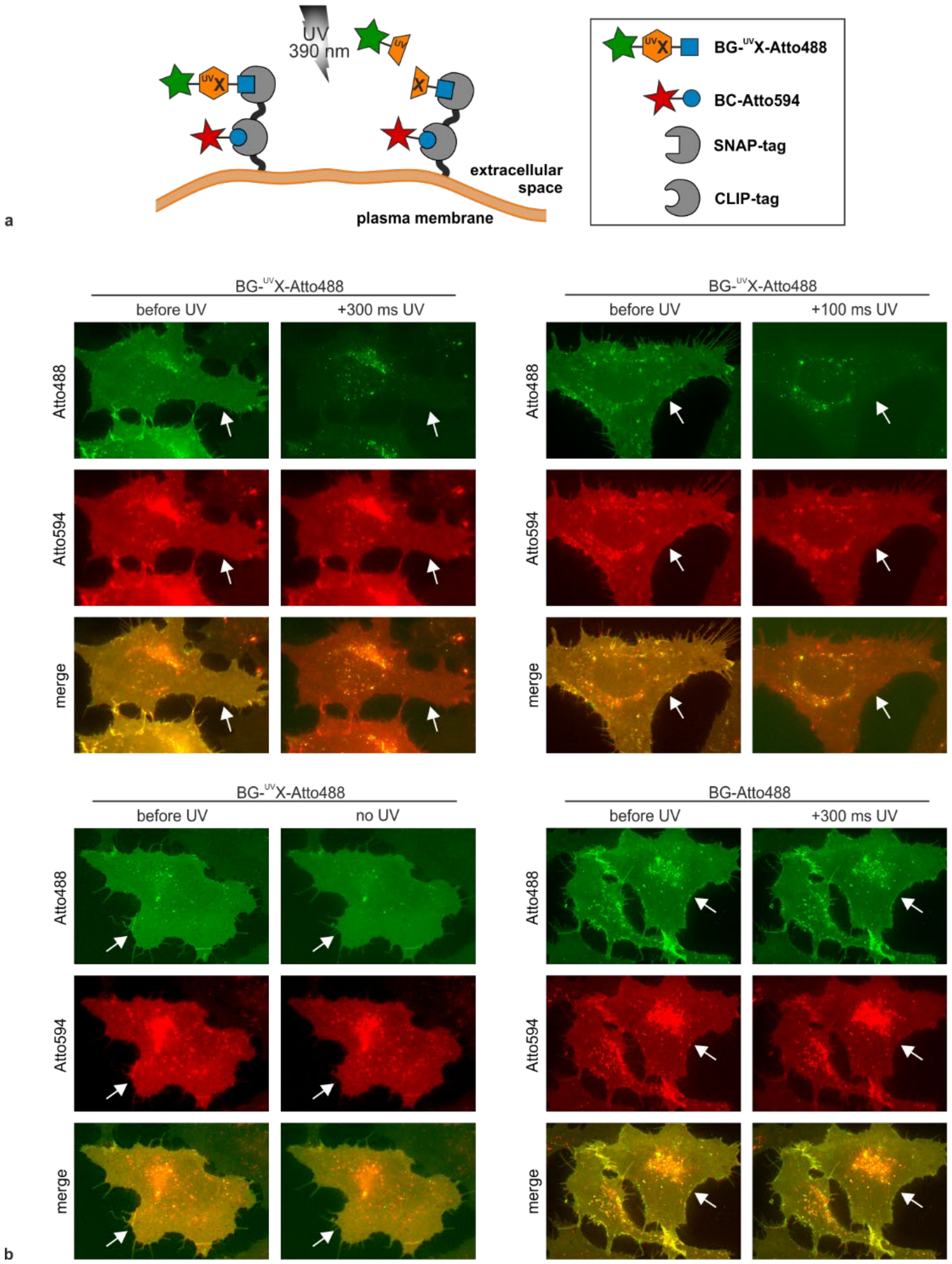

Figure S4. **Characterization of the light-triggered cleavage reaction.** ***a)*** *Scheme of cell surface labeling with BG-^{UV}X-Atto488 and BC-Atto594. A fusion protein containing SNAP-tag and CLIP-tag (SNAP-CLIP display) was localized to the plasma membrane of HeLa cells. A CLIP-tag substrate carrying a red fluorescent dye (BC-Atto594) and a SNAP-tag substrate carrying a green fluorescent dye (BG-Atto488,* ***16****) are added to the medium. If a photocleavable linker is placed between benzyl guanine and Atto488 (BG-^{UV}X-Atto488,* ***17****) the green surface stain can be removed under the microscope by a short UV light pulse.* ***b)*** *Live cells expressing the SNAP-CLIP display construct were stained with BC-Atto594 and BG-Atto488 or BG-^{UV}X-Atto488. Both dyes successfully stained the cell surface (marked on one edge with a white arrow for orientation). Upon UV irradiation (390 nm, lumencor® AuraII, 100 ms or 300 ms) the red signal (BC-Atto594) remains unaffected, but the green signal (BG-^{UV}X-Atto488) is removed immediately. This is not the case in the absence of UV light or if the non-cleavable BG-Atto488 is used.*

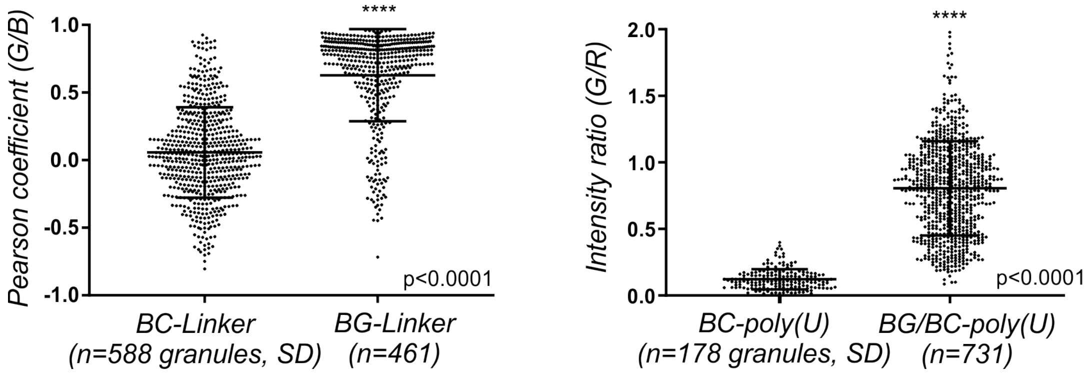

Figure S5. Quantification of orthogonal recruitment of two different proteins to stress granules. a) *Recruitment of a BG-poly(U)-guideRNA into arsenite-induced stress granules (50 µM As_2O_3, 30 min) in a transgenic HeLa cell line, stably expressing a SNAP-$(eGFP)_3$ reporter and a BFP-G3BP1 stress granule marker. Arsenite-induced stress granules appear as blue foci. The BG-poly(U)-guideRNA gives co-staining in the green channel, whereas the control BC-poly(U)-guideRNA does less, as indicated by the Pearson coefficient for the correlation of green (G) and blue (B) fluorescence at N ≥461 blue foci. Statistical significance was calculated by an unpaired Welch's t-test. Details of experimental procedures and image processing can be found in the supplementary information.* ***b)*** *Orthogonal recruitment of two proteins. Transgenic HeLa cells, stably co-expressing SNAP-$(eGFP)_3$ and CLIP-$(mCherry)_3$, were either transfected with a poly(U)-guideRNA carrying the BG, BC, or bifunctional BG/BC self-labeling moieties. The red channel (R) monitors recruitment of mCherry; the green channel (G) monitors recruitment of eGFP upon arsenite-induced stress granule formation. The intensity ratio (green/red; G/R) was measured at N ≥178 red foci. Statistical significance was calculated by an unpaired Welch's t-test. Details of experimental procedures and image processing can be found in the supplementary information.*

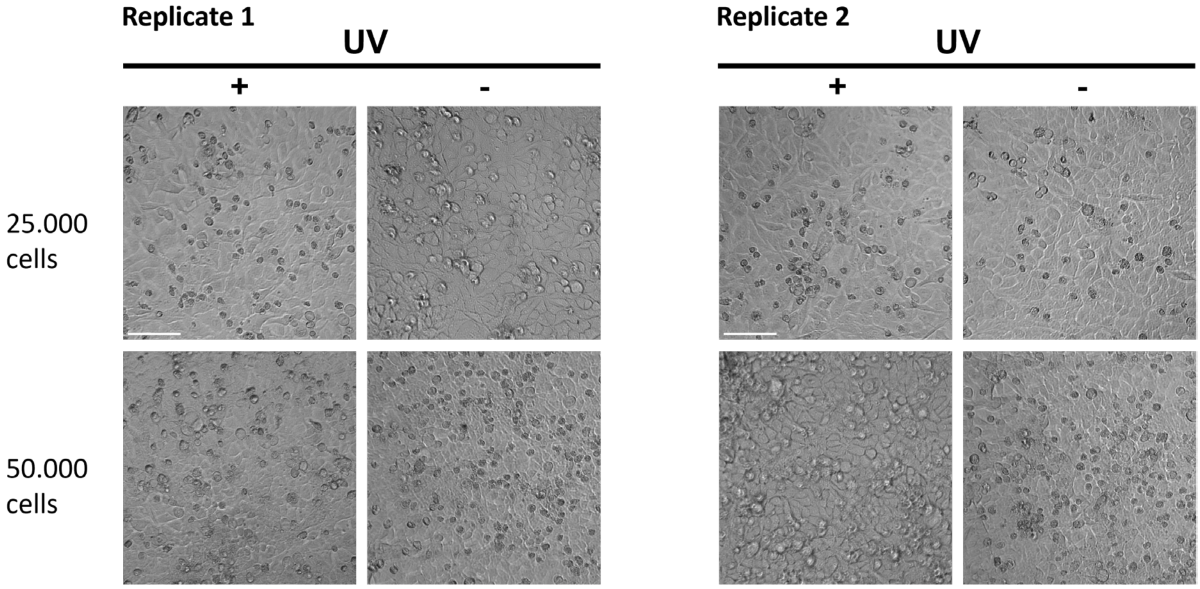

Figure S6. **The applied amount UV light did not lead to visible toxicity.** *SNAP-ADAR1Q Flp-In cells were seeded at a density of 25.000 or 50.000 cells / 96 well with doxycycline (10 ng/mL) and were grown for 24 hours, followed by the irradiation of UV (365 nm) for 0 min (- UV) versus 3 min (+ UV). After further incubation of 20 hours, microscopy observation was performed for morphology evaluation. Pictures were recorded with a 40x air objective. Scale bar 100 µm.*

Photodissociation Kinetics of BG-^{UV}X-OH

To determine the decaging efficiency εΦ, the decay of BG-^{UV}X-OH (**8**) was compared to the decaging of the previously characterized $^{N7\text{-}Npom}$BG-TFA as described before. The latter was chosen because of the similar absorbance properties of their photocleavable moieties (see supporting Figure S1a). For both substances the extinction coefficient was estimated to be ε_{365nm} = 4.3 mM^{-1}cm^{-1} as determined for Npom-OH. Stock solutions of BG-^{UV}X-OH and $^{N7\text{-}Npom}$BG-TFA were prepared in DMSO and diluted in PBS (137 mM NaCl, 2.7 mM KCl, 12 mM K_2HPO_4, 12 mM KH_2PO_4, pH = 7.4) to a final concentration of 10 µM (DMSO < 0.02%). Decaging was performed in PCR tubes by irradiation with 365 nm light on a UV transilluminator (UVP TFL-40V, 25 W power, intensity high, giving 7.9±0.2 mW/cm^2 on the sample) for the indicated amount of time at room temperature. Samples were covered with aluminium foil and stored at –20°C until they were applied to analytical HPLC with UV detection at 280nm and 365 nm (see supporting Figures S1d and S1f). Decomposition of $^{N7\text{-}Npom}$BG-TFA (**D**) results in two clean products, BG-TFA (**F**) which shows high absorption at 280nm but no absorption at 365 nm and the released caging group (**E**) that shows high absorbance at 365nm (see supporting Figures S1e and S1f). The photocleavable linker BG-^{UV}X-OH (**A**) decomposed to several peaks on the HPLC (see supporting Figures S1c and S1d), one of which can be assigned to be (4-hydroxybenzoyl)glycine (**C**) by LC-MS and comparison to the reference substance synthesized (spectra below). (4-Hydroxybenzoyl)glycine is the cleavage product that remains on a guideRNA after irradiation in editing experiments and therefore demonstration of its timely release is most relevant for this study. Another peak could be assigned to be the nitroso acetophenone product (**B**) containing the benzyl guanine by LC-MS which shows absorption at 365 nm (spectra below). The peaks were integrated with the Shimadzu VP-Class software.

The peak area of the cleavage products was plotted against irradiation time (see Figure 1c) and by logarithmic fit the half-time was determined to be $t_{1/2}$ = 17±2 s for (4-hydroxybenzoyl)glycine and $t_{1/2}$ = 15±3 s for BG-TFA. With the quantum yield of the reference substance ($^{N7\text{-}Npom}$BG-TFA Φ ≈ 0.5), the quantum yield of BG-^{UV}X-OH can be calculated to be Φ ≈ 0.4 according to following formula:

$$\Phi_{BG-{}^{UV}X-OH} = \Phi_{Reference} \times \frac{t_{1/2\,reference}}{t_{1/2\,BG-{}^{UV}X-OH}} \times \frac{\varepsilon_{reference}}{\varepsilon_{BG-{}^{UV}X-OH}} = 0.41$$

Table of the relative peak area of BG-^{UV}X-OH and $^{N7\text{-}Npom}$BG-TFA and their cleavage products upon UV irradiation as determined in analytical HPLC:

	relative peak area			
time [s]	BG-^{UV}X-OH	(4-hydroxy-benzoyl)glycine	$^{N7\text{-}Npom}$BG-TFA	BG-TFA
0	100.0	0.0	100.0	0.0
15	56.7	35.6	53.2	35.7
30	28.4	55.0	23.3	70.0
45	14.2	69.9	9.3	77.3
60	5.7	85.7	2.9	83.5
180	0.0	100.0	0.0	100.0

UV chromatogram of the LC-MS analysis of BG-^{UV}X-OH after partial photo-dissociation (corresponds to supporting Figure S1d). Peaks corresponding to BG-^{UV}X-OH (**A**) and its decay products (**B**) and (**C**, (4-hydroxybenzoyl)glycine) are marked. See also supporting Figure S1c.

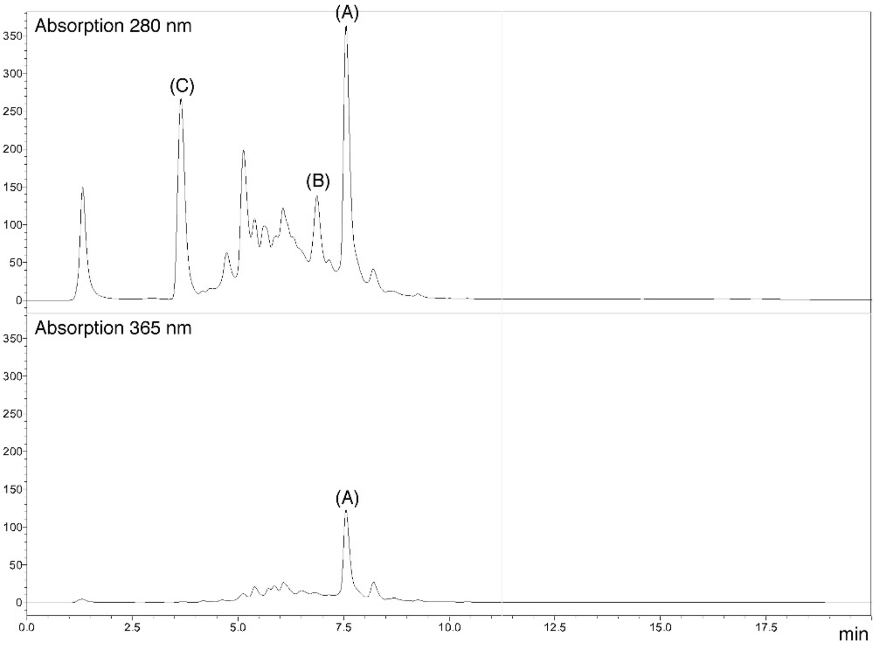

Extracted MS spectrum of BG-^{UV}X-OH (**A**), positive mode (top) and negative mode (bottom)

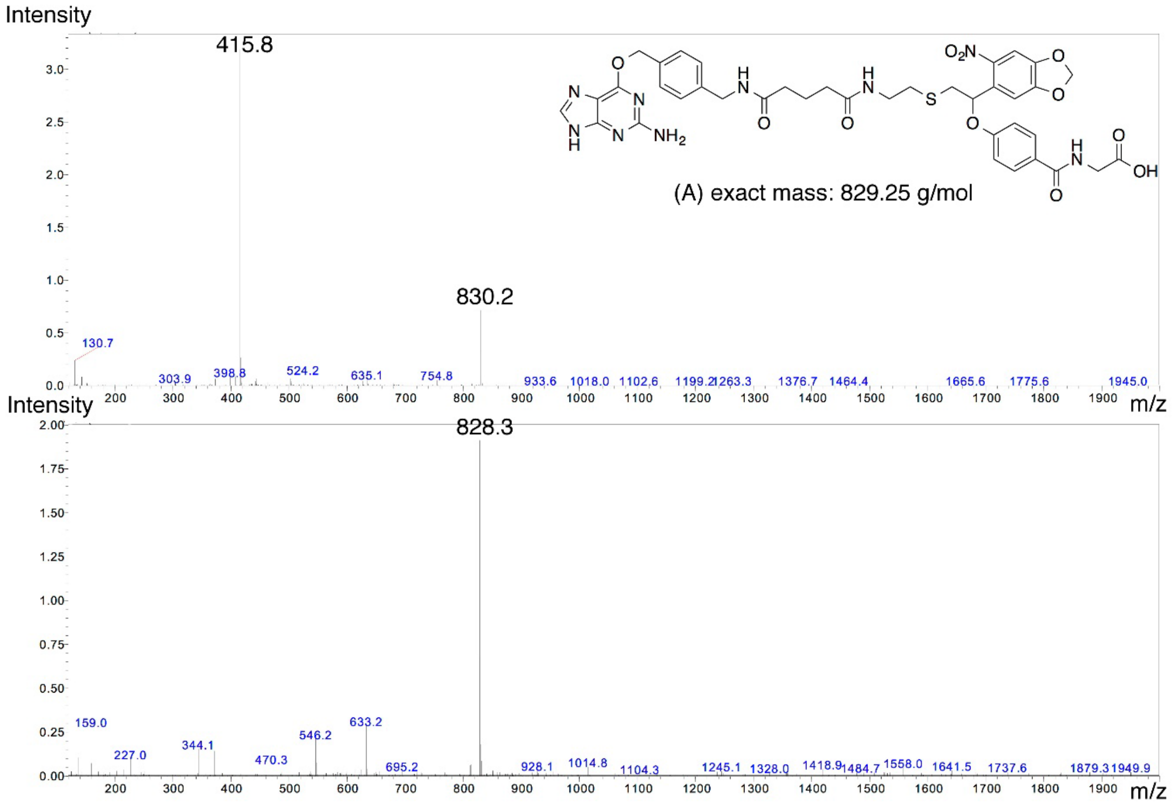

Extracted MS spectrum of peak (**B**), corresponding to the nitroso acetophenone decay product of BG-^{UV}X-OH, positive mode (top) and negative mode (bottom)

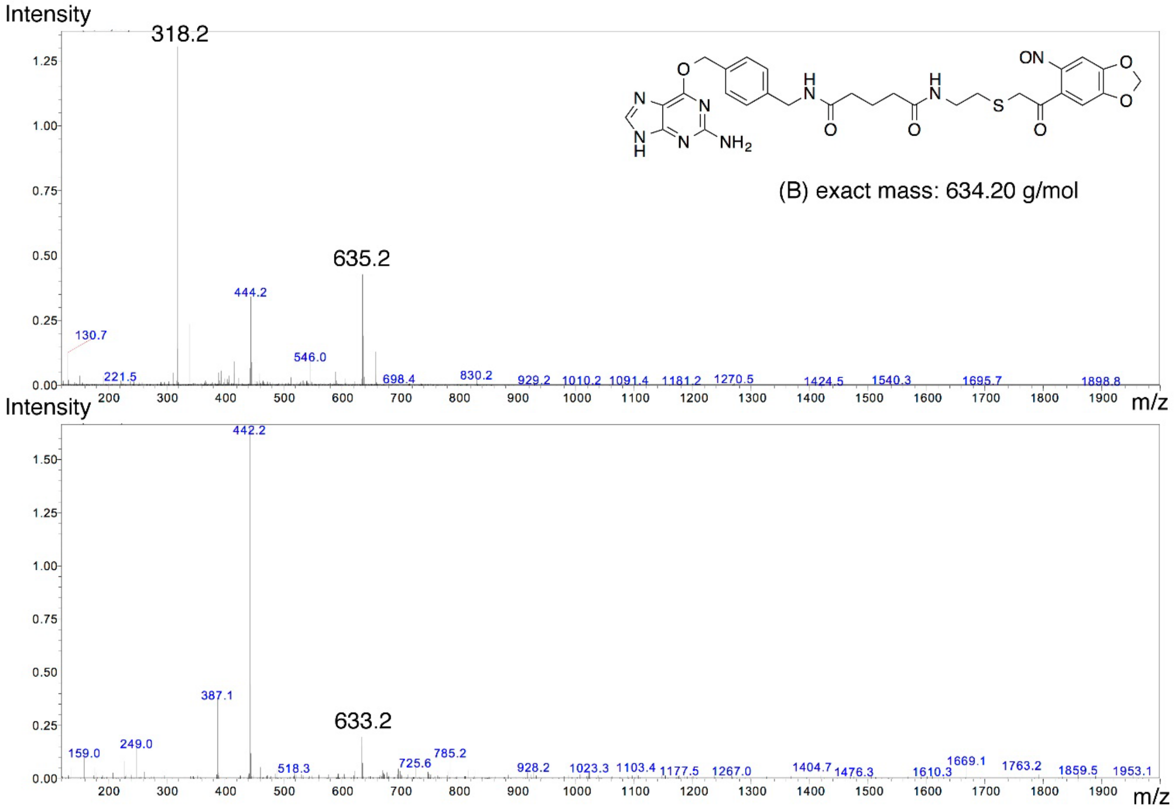

Extracted MS spectrum of peak (**C**), corresponding to (4-hydroxybenzoyl)glycine, positive mode (top) and negative mode (bottom)

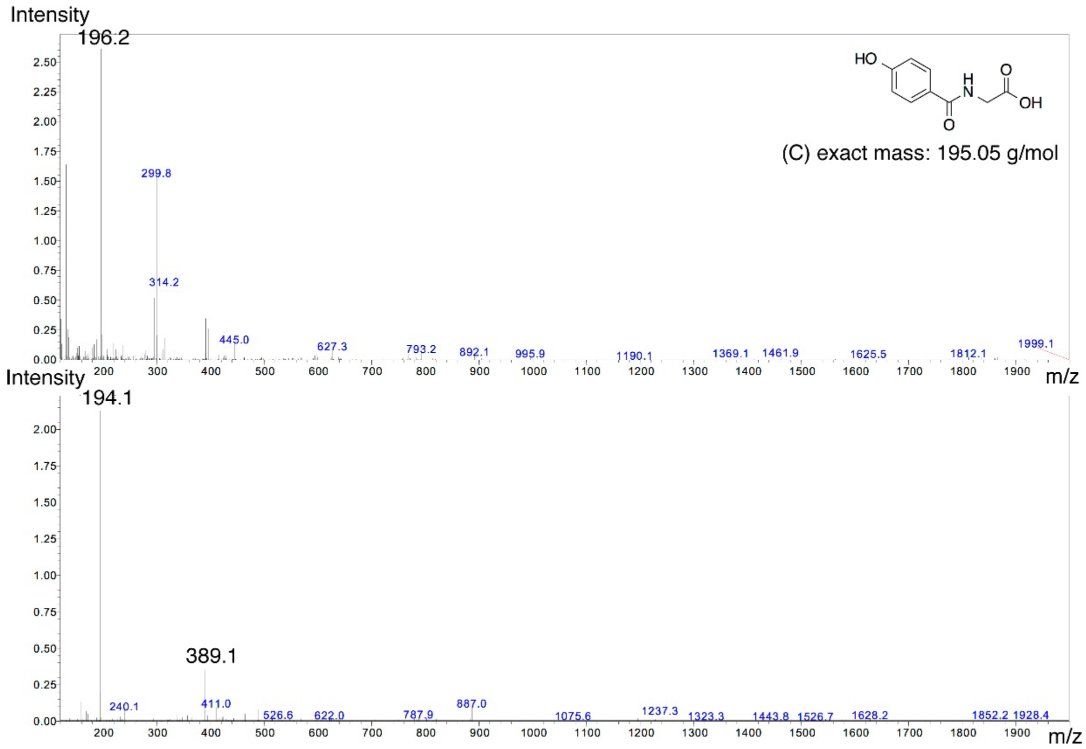

UV chromatogram of LC-MS analysis and extracted MS spectrum of synthesized (4-hydroxybenzoyl)glycine (**10**), positive mode (top) and negative mode (bottom). Elution time and detected m/z ratios correspond to decaging product (**C**) detected in the LC-MS analysis of partially photo-cleaved BG-^{UV}X-OH.

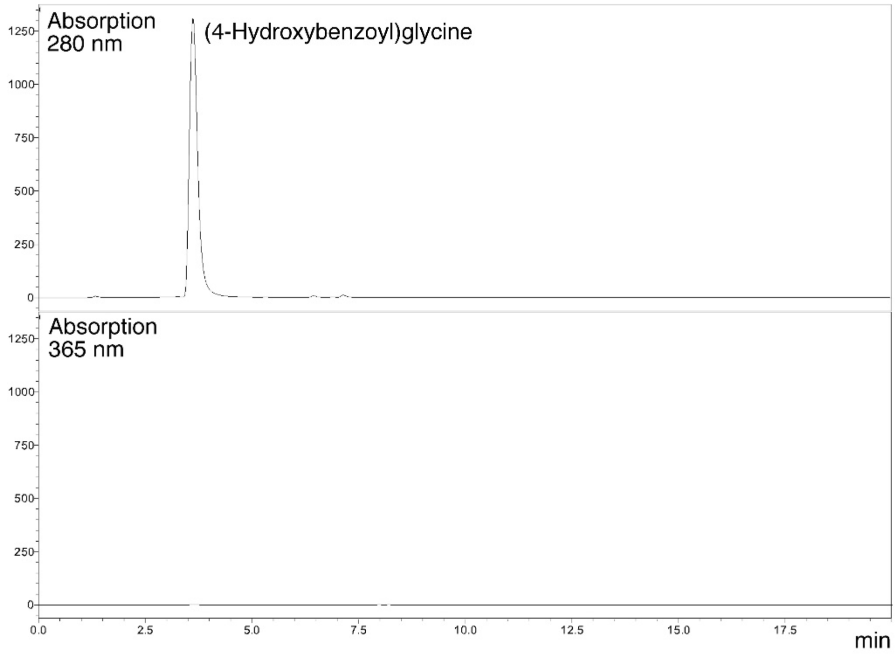

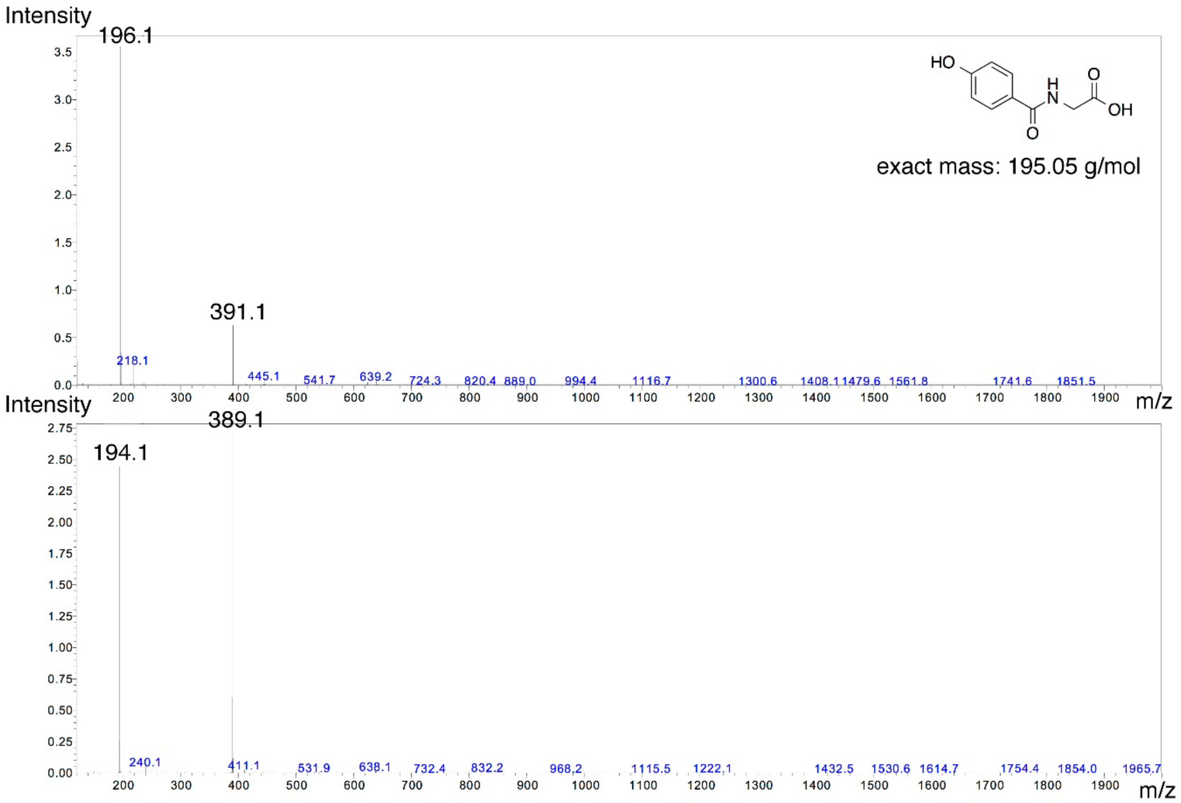

Chemical synthesis

Chemicals

If not stated otherwise, all substrates and reagents required for synthesis and biochemical studies were purchased from commercial providers and used without further purification.

O^6-(4-aminomethyl-benzyl)guanine (BG-NH_2) was synthesized as described in literature. 2-(4-(Aminomethyl)-benzyloxy)-4-aminopyrimidine (BC-NH_2) was prepared from commercially available methyl-4-(aminomethyl) benzoate hydrochloride according to literature. We recently described a detailed synthesis of $^{N7\text{-}Npom}$BG-TFA and $^{N7\text{-}Npom}$BG-NH_2.

General Methods

All column chromatographic purifications were carried out on self-packed columns of silica gel (0.04-0.063 mm/230-240 mesh). Thin-layer chromatography (TLC) was performed on silica gel sheets (60 F254, 0.2 mm, 5 x 10 cm, Merck) and visualized under UV light (254 nm). All analytical and preparative HPLC runs were performed on a Shimadzu system (SCL-10A VP, SPD-20AV, LC-20AT) running with 0.1% TFA in water (eluent A) and 0.1% TFA in acetonitrile/water (9:1, eluent B). Analytical HPLC was performed using an EC 125/4 Nucleodur C18 column by Machery + Nagel with a linear gradient from 5% eluent B (starting after 1 min) to 95% eluent B (ending after 25 min). Preparative HPLC was performed using a VP 250/10 Nucleodur C18 column by Machery + Nagel. High resolution mass spectrometry was performed on a maXis4G ESI-TOF-MS by Bruker Daltonics. LC-MS analyses were conducted on a LC-MS2020 (Shimadzu) with a Kinetex C18 column (Phenomonex). 0.1% formid acid in water (eluent A) and 0.1% formic acid in acetonitrile/water (4:1, eluent B) were used as eluents. A linear gradient from 5% eluent B (starting after 1 min) to 95% eluent B (ending after 11 min) was used. NMR spectra were recorded on a Bruker ARX 250 spectrometer at 250 MHz for ^{1}H spectra and 63 MHz for ^{13}C spectra or a Bruker Avance 400 spectrometer at 400MHz for ^{1}H spectra and 101 MHz for ^{13}C spectra. 2D-NMR spectra were recorded with a Bruker Avance 400 at 400 MHz/101 MHz.

Synthesis of Fmoc-^{UV}X-OH

The photo-cleavable amino acid Fmoc-^{UV}X-OH was synthesized in a five-step synthesis starting with the bromination of 4’,5’-methylenedioxy-2’- nitroacetophenone which was carried out following the description of Pendrak et al. The brominated product was reacted with Fmoc-protected cysteamine which was synthesized following to the protocol of Miura et al. The full synthesis scheme is shown below.

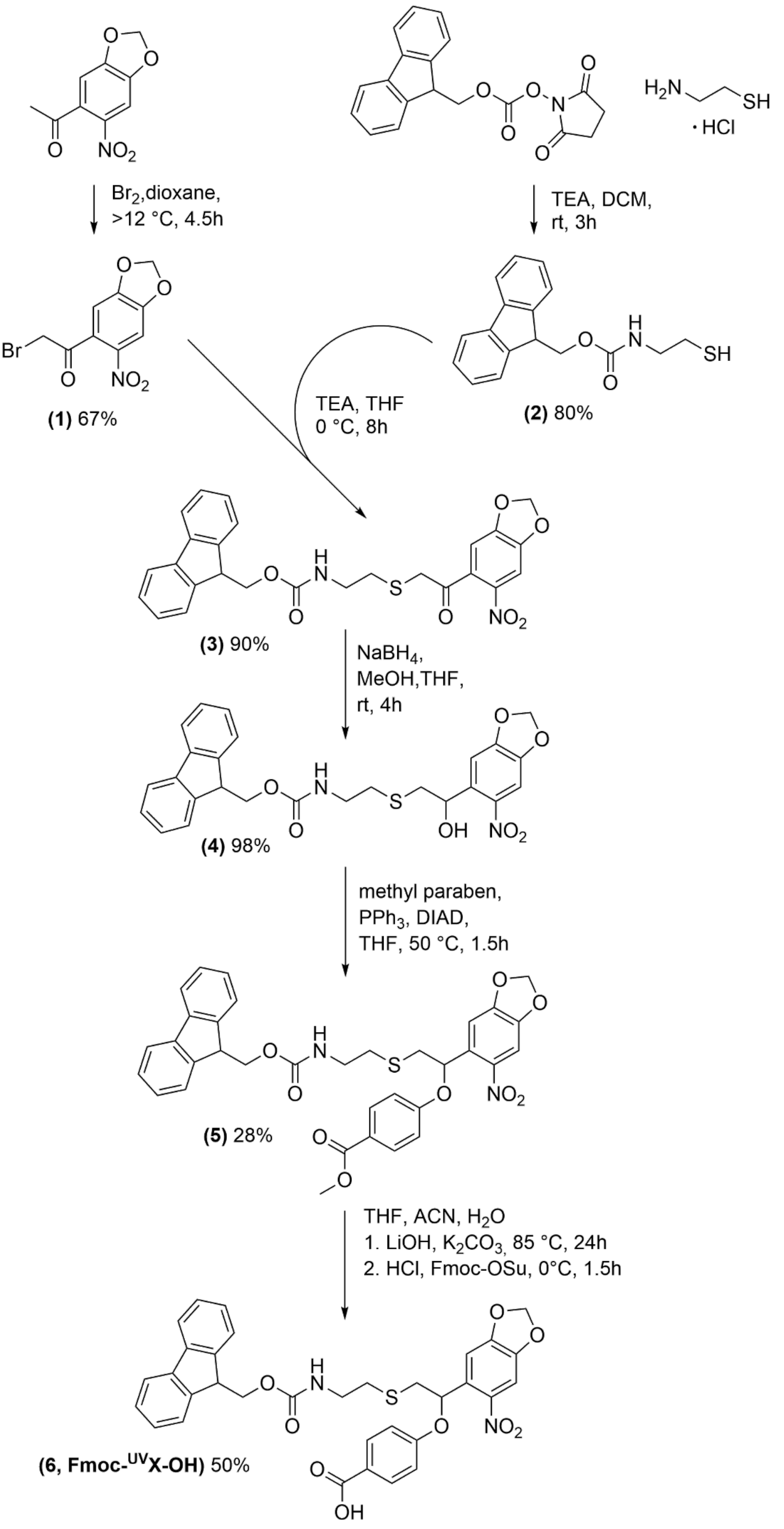
O
O
NO2
H2N
SH
· HCl
Br2,dioxane,
>12 °C, 4.5h
TEA, DCM,
rt, 3h
Br
NO2
(1) 67%
TEA, THF
0 °C, 8h
H
N
SH
(2) 80%
S
NO2
(3) 90%
NaBH4,
MeOH,THF,
rt, 4h
OH
(4) 98%
methyl paraben,
PPh3, DIAD,
THF, 50 °C, 1.5h
(5) 28%
THF, ACN, H2O
1. LiOH, K2CO3, 85 °C, 24h
2. HCl, Fmoc-OSu, 0°C, 1.5h
(6, Fmoc-UVX-OH) 50%

2-Bromo-1-(6-nitrobenzo[d][1,3]dioxol-5-yl)ethan-1-one (1)

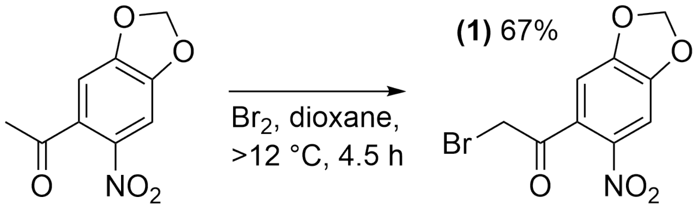

A solution of 4',5'-methylenedioxy-2'-nitroacetophenone (4 g, 19.12 mmol, 1.0 eq) in dioxane (12 ml) was stirred on ice and a solution of bromine (1.1 ml, 21.03 mmol, 1.1 eq) in dioxane (44 ml) was added dropwise. The reaction was stirred for 4.5 h with occasional cooling. The solvent was evaporated, and the residue was solved in Et_2O, washed with sodium thiosulfate and brine, and dried over Na_2SO_4. The solvent was removed in vacuo and the crude product was crystallized from cyclohexane (110 ml) and DCM (45 ml). Evaporation of the solvents resulted in 3.8 g (67%) of **1** as a yellow solid.

R_f (CH/Et_2O, 7:5) = 0.21

^{1}H-NMR (250 MHz, $CDCl_3$) δ [ppm] = 7.63 (s, 1H), 6.83 (s, 1H), 6.22 (s, 2H), 4.24 (s, 2H).

^{13}C-NMR (63 MHz, $CDCl_3$) δ [ppm] = 193.5, 153.0, 149.5, 131.7, 108.2, 104.7, 140.0, 97.8, 33.8.

Analytical HPLC of **1** after crystallization

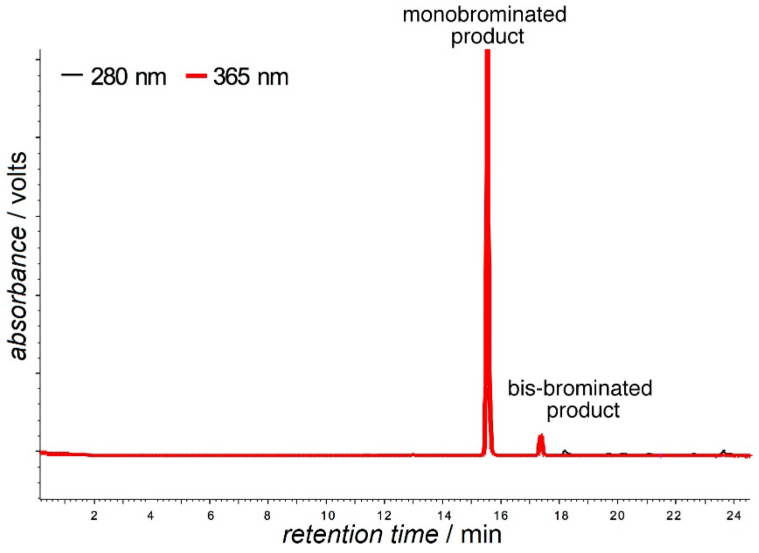

^{1}H-NMR (250 MHz, $CDCl_3$) of **1**

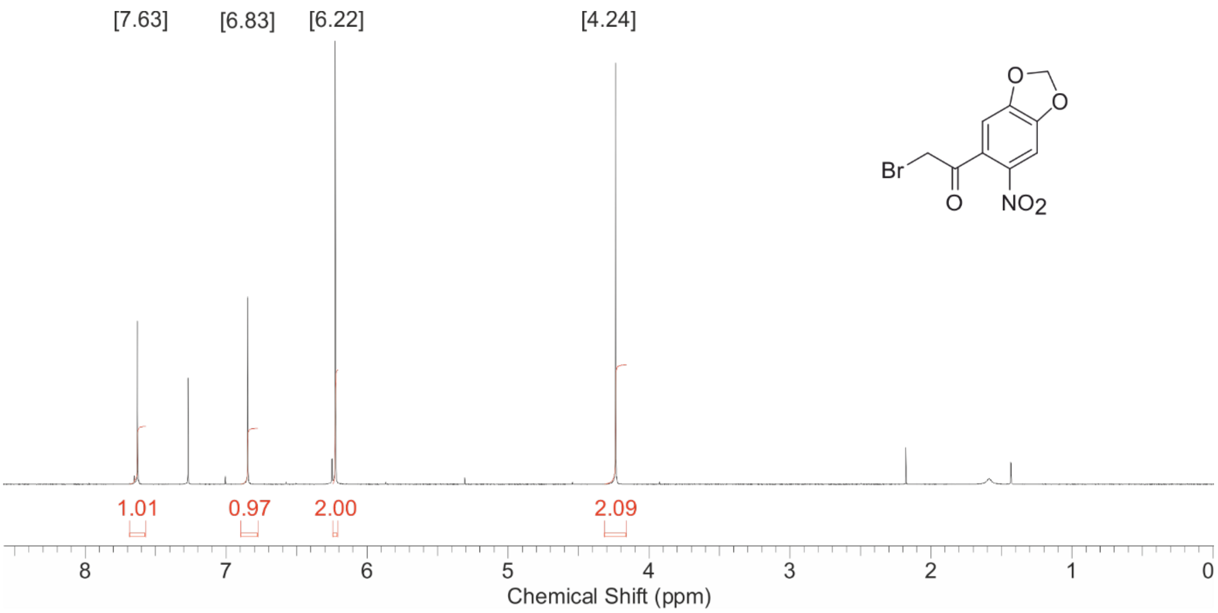

^{13}C-NMR (63 MHz, $CDCl_3$) of **1**

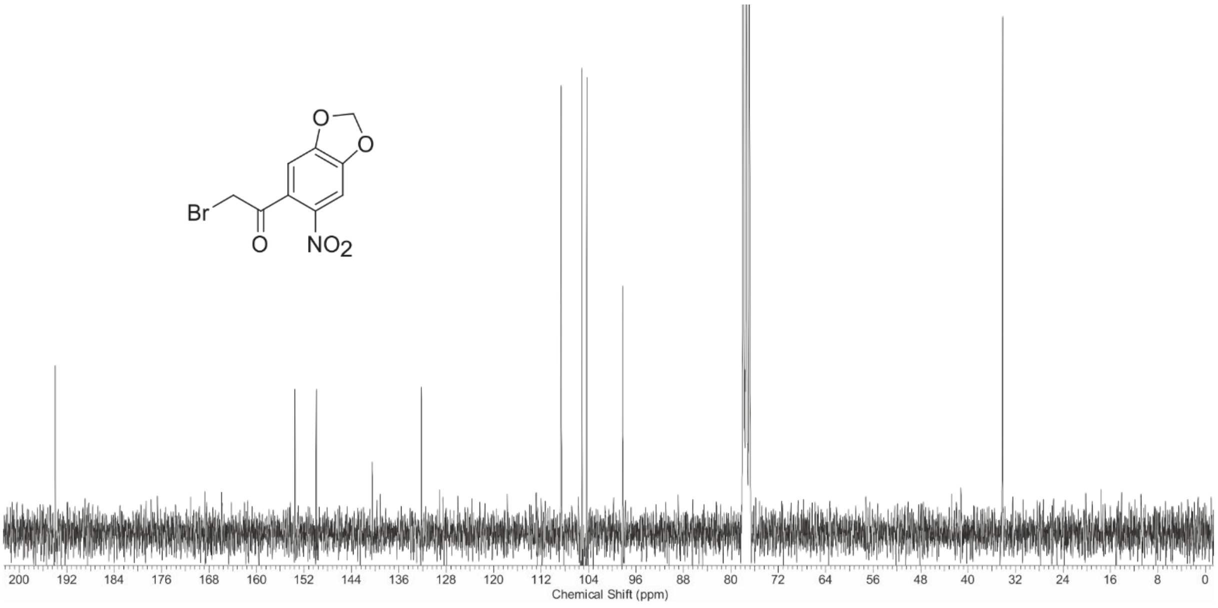

(9H-Fluoren-9-yl)methyl (2-mercaptoethyl)carbamate (2)

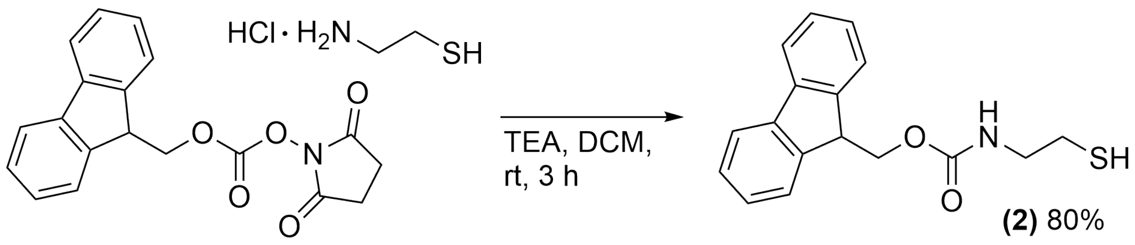

(2) 80%

Fmoc-OSu (10.0 g, 29.6 mmol, 1.0 eq) was solved in DCM (40 ml) and stirred on ice under an argon atmosphere. Cysteamine hydrochloride (3.34 g, 29.4 mmol, 1.0 eq) in DCM (40 ml) and TEA (8.15 ml, 58.8 mmol, 2.0 eq) were added to the reaction mixture. After 3 h, the solution was washed with 0.1 M HCl and brine and the organic phase was dried over Na_2SO_4. After evaporation of the solvent, 7.08 g (80%) of a white solid were obtained. The product showed little impurities in HPLC and was used for the next reactions without further purification.

R_f (CH/EA, 2:1) = 0.53

^{1}H-NMR (250 MHz, $CDCl_3$) δ [ppm] = 7.79 (d, *J* = 7.1 Hz, 2H), 7.61 (d, *J* = 7.3 Hz, 2H), 7.38 (m, 4H), 5.14 (br s, 1H), 4.45 (d, *J* = 6.6 Hz, 2H), 4.24 (t, *J* = 6.6 Hz, 1H), 3.38 (q, *J* = 6.0 Hz, 2H), 2.66 (q, *J* = 7.2 Hz, 2H), 1.34 (t, *J* = 8.5 Hz,1H).

Analytical HPLC of crude product of **2**

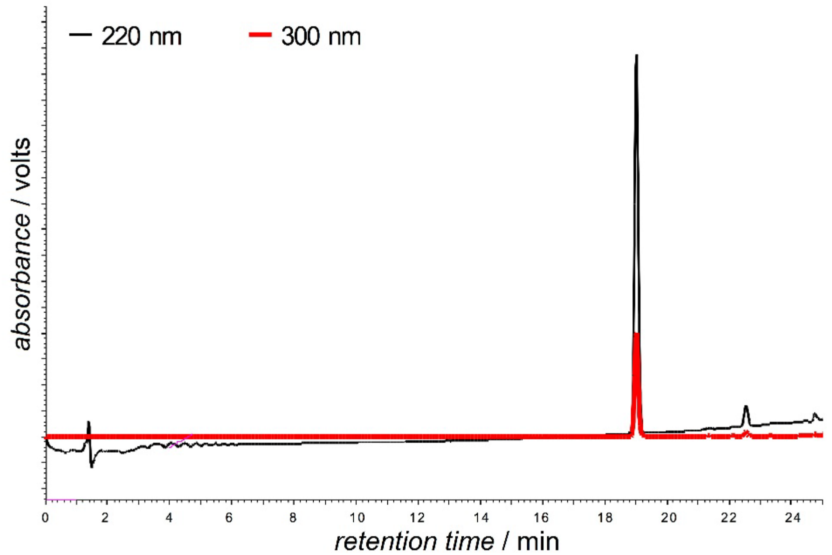

^{1}H-NMR (250 MHz, $CDCl_3$) of **2**

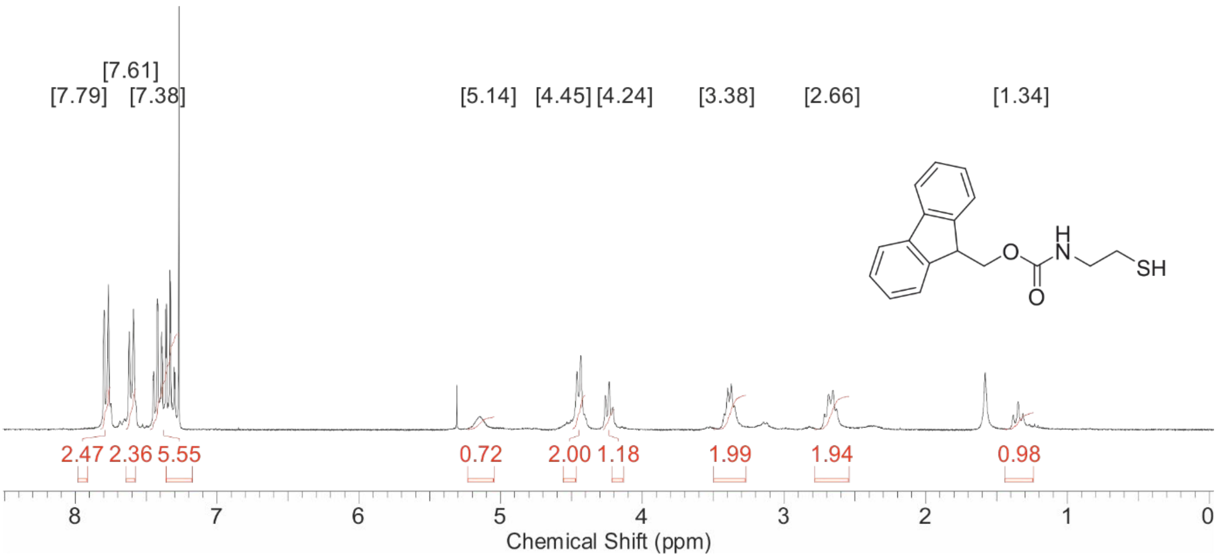

(9H-Fluoren-9-yl)methyl (2-((2-(6-nitrobenzo[d][1,3]dioxol-5-yl)-2-oxoethyl)-thio)ethyl)carbamate (3)

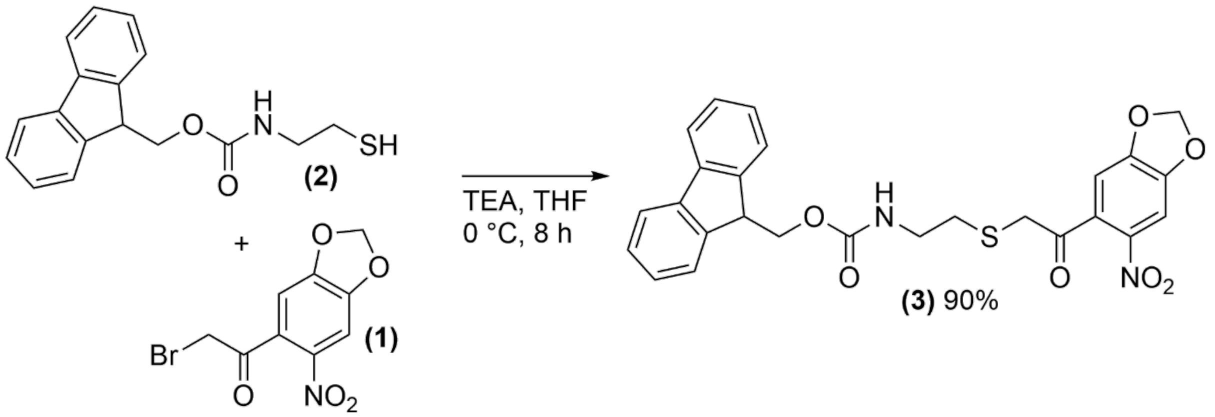

Compound **2** (3.9 g, 13.0 mmol, 1.0 eq) was solved in THF (100 ml) under argon atmosphere and TEA (2 ml, 14.3 mmol, 1.1 eq) was added dropwise while cooling the reaction on ice. Afterwards the mixture was added to a solution of **1** (3.75 g, 13.0 mmol, 1.0 eq) in THF (200 ml). After 4 h, another 0.5 eq of **1** (1.88 g, 6.5 mmol) and 0.5 eq of TEA (0.91 ml, 6.5 mmol) were added and the reaction was stirred for another 4 h. The solvent was removed in vacuo and the residue dissolved in EA, washed with 0.1 M HCl and brine, and dried over Na_2SO_4. The removal of the solvent yielded 10.2 g brownish raw product, which was purified by flash column chromatography (CH/EA, 2:1–1:2). 6.7 g (90%) of a yellow-orange solid were obtained.

R_f (CH/EA, 2:1) = 0.15

^{1}H-NMR (250 MHz, $CDCl_3$) δ [ppm] = 7.78 (d, J = 7.6 Hz, 2H), 7.60 (d, J = 7.6 Hz, 2H), 7.60 (s, 1H), 7.38 (m, 4H), 6.85 (s, 1H), 6.17 (s, 2H), 5.22 (br s, 1H), 4.45 (d, J = 7.1 Hz, 2H), 4.25 (t, J = 6.8 Hz, 1H), 3.65 (s, 2H), 3.46 (q, J = 5.9 Hz, 2H), 2.77 (t, J = 6.1 Hz, 2H).

^{13}C-NMR (63 MHz, $CDCl_3$) δ [ppm] = 195.9, 156.3, 152.9, 149.3, 143.9, 141.3, 127.6, 126.8, 125.1, 120.0, 108.0, 104.6, 103.8, 66.9, 60.5, 47.3, 40.7, 32.3, 21.1, 14.2.

HR-ESI-MS: $[M+Na]^{+}_{(theor.)}$ = 529.10399, $[M+Na]^{+}_{(meas.)}$ = 529.10439.

^{1}H-NMR (250 MHz, $CDCl_3$) of **3**

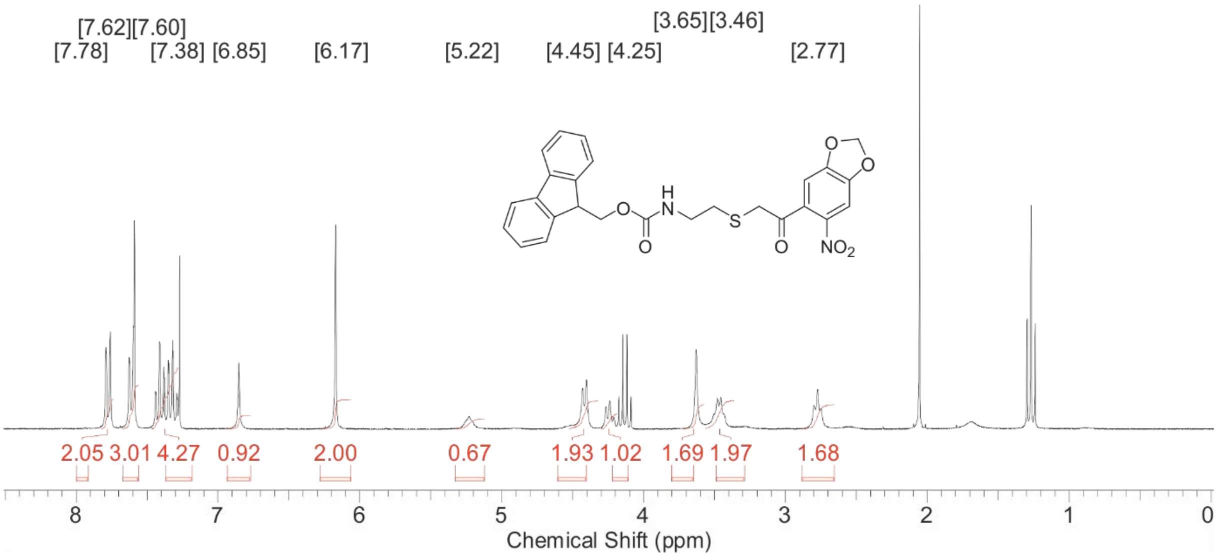

^{13}C-NMR (63 MHz, $CDCl_3$) of **3**

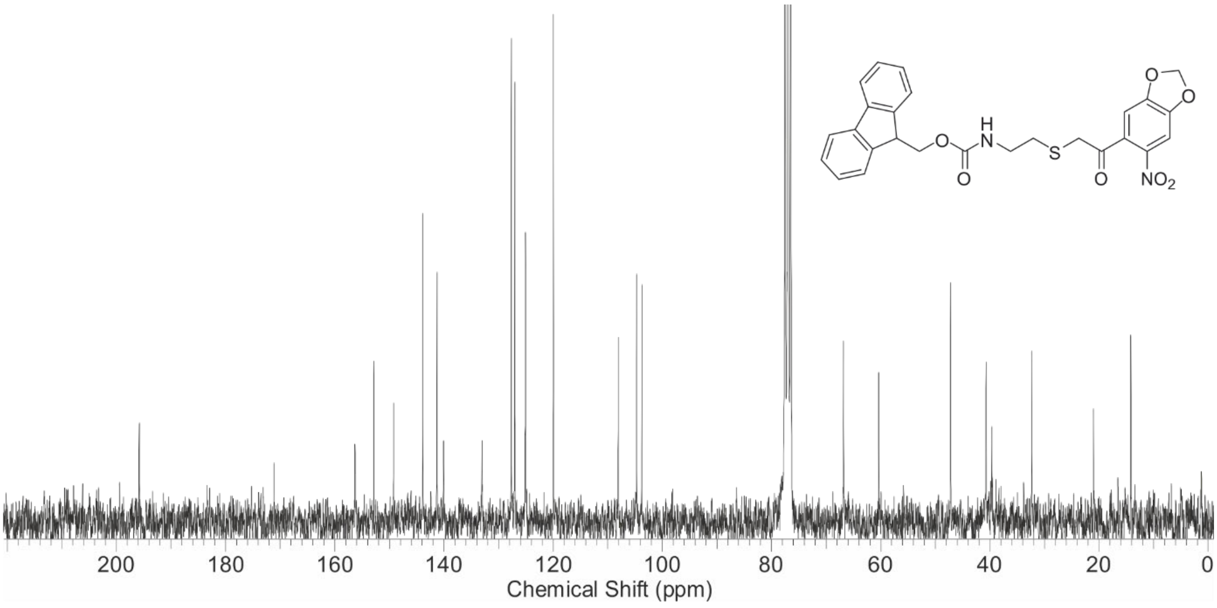

((9H-Fluoren-9-yl)methyl (2-((2-hydroxy-2-(6-nitrobenzo[d][1,3]dioxol-5-yl)ethyl)thio)ethyl)carbamate (4)

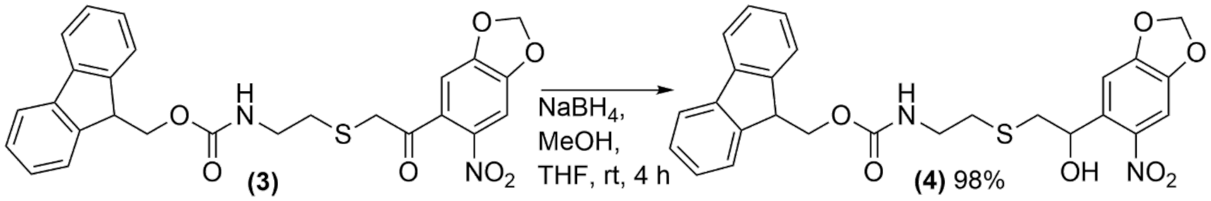

3 (6.5 g, 12.8 mmol, 1.0 eq) was solved in MeOH (350 ml), THF (50 ml) and $NaBH_4$ (0.49 g, 12.8 mmol, 1.0 eq) and the reaction was stirred for 4 hours at room temperature. The solvation was facilitated by sonication. Subsequently, the reaction was quenched with a saturated solution of NH_4Cl and the product extracted with EA. The organic phase was washed with brine and dried over Na_2SO_4. After removal of the solvent, 6.4 g (98%) of yellow-brown product were obtained which was used in the following reactions without further purification.

R_f (CH/EA, 2:1) = 0.23

^{1}H-NMR (400 MHz, $CDCl_3$) δ [ppm] = 7.77 (d, J = 7.6 Hz, 2H), 7.60 (d, J = 7.3 Hz, 2H), 7.51 (s, 1H), 7.36 (m, 4H), 7.36 (s, 1H), 6.11 (d, J = 6.1 Hz, 2H), 5.43 (d, J = 8.8 Hz, 1H), 5.22 (t, J = 4.0 Hz, 1H), 4.46 (d, J = 6.3 Hz, 2H), 4.23 (t, J = 6.8 Hz, 1H), 3.51 (m, 2H), 3.19 (d, J = 14.2 Hz, 1H), 2.84 (m, 2H), 2.53 (m, 2H).

^{13}C-NMR (101 MHz, $CDCl_3$) δ [ppm] = 156.6, 152.7, 147.2, 143.9, 141.3, 136.0, 127.6, 127.1, 125.0, 119.9, 106.7, 105.2, 103.0, 67.7, 66.8, 53.5, 47.3, 40.8, 40.5, 32.1.

HR-ESI-MS: $[M+Na]^+_{(theor.)}$ = 531.11964, $[M+Na]^+_{(meas.)}$ = 531.11969.

^{1}H-NMR (400 MHz, $CDCl_3$) of **4**

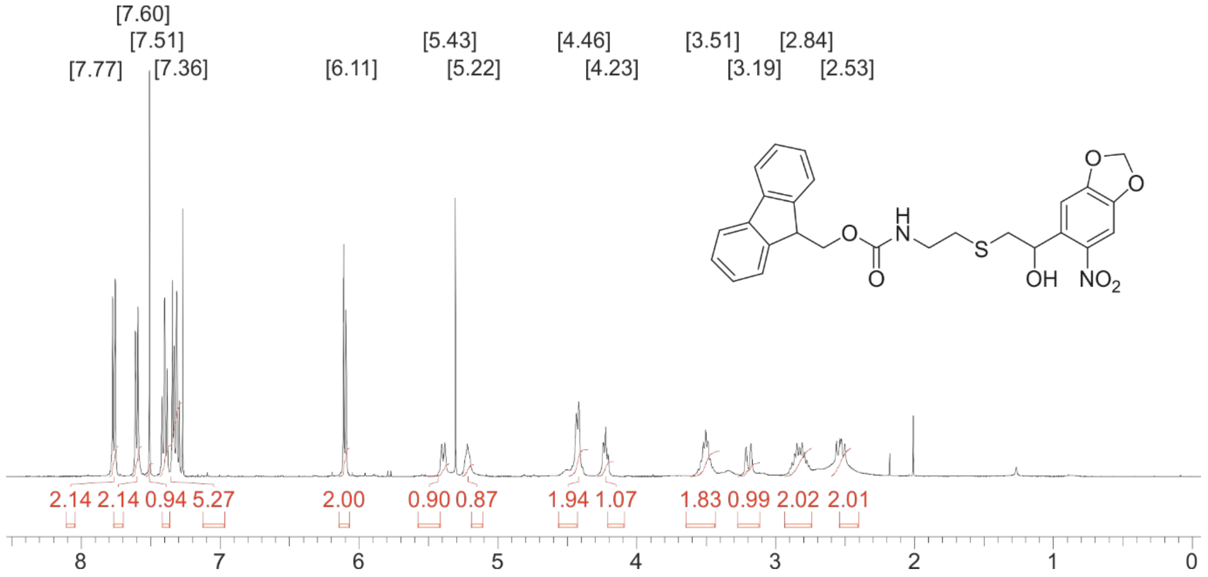

^{13}C-NMR (101 MHz, $CDCl_3$) of **4**

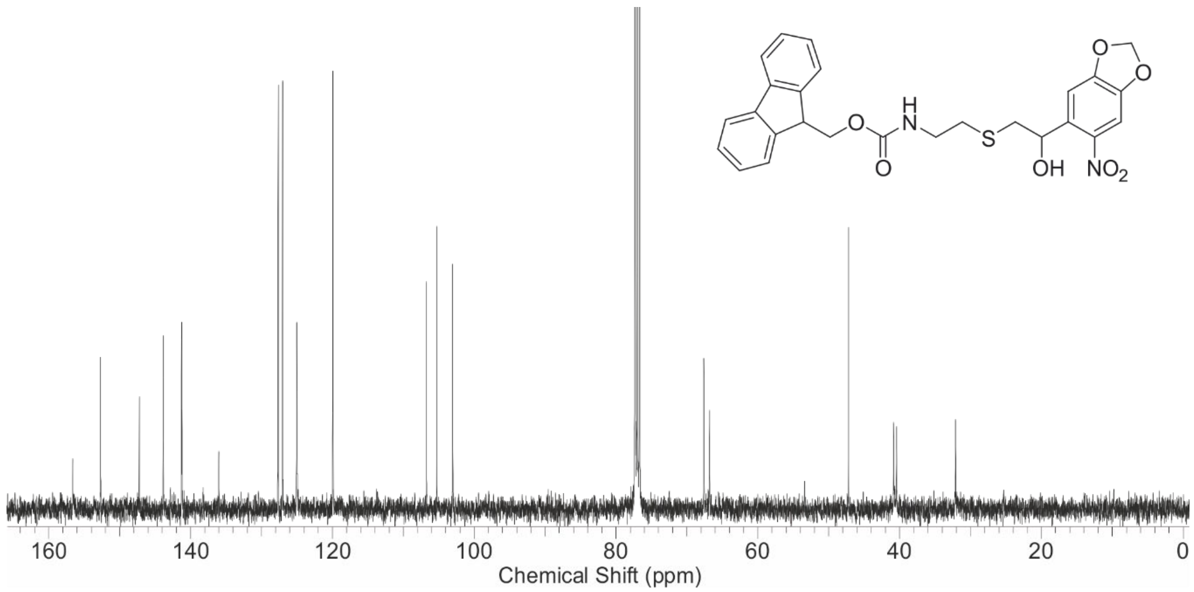

2D-NMR ^{1}H,^{1}H-COSY of **4**

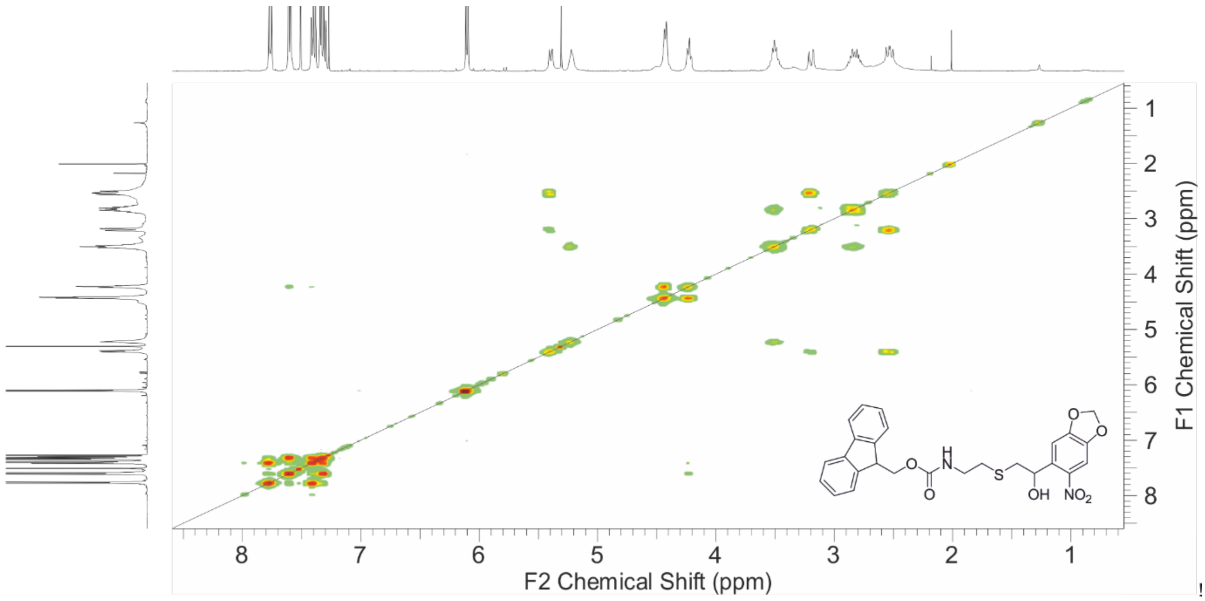

2D-NMR ^{1}H,^{13}C-HSQC of **4**

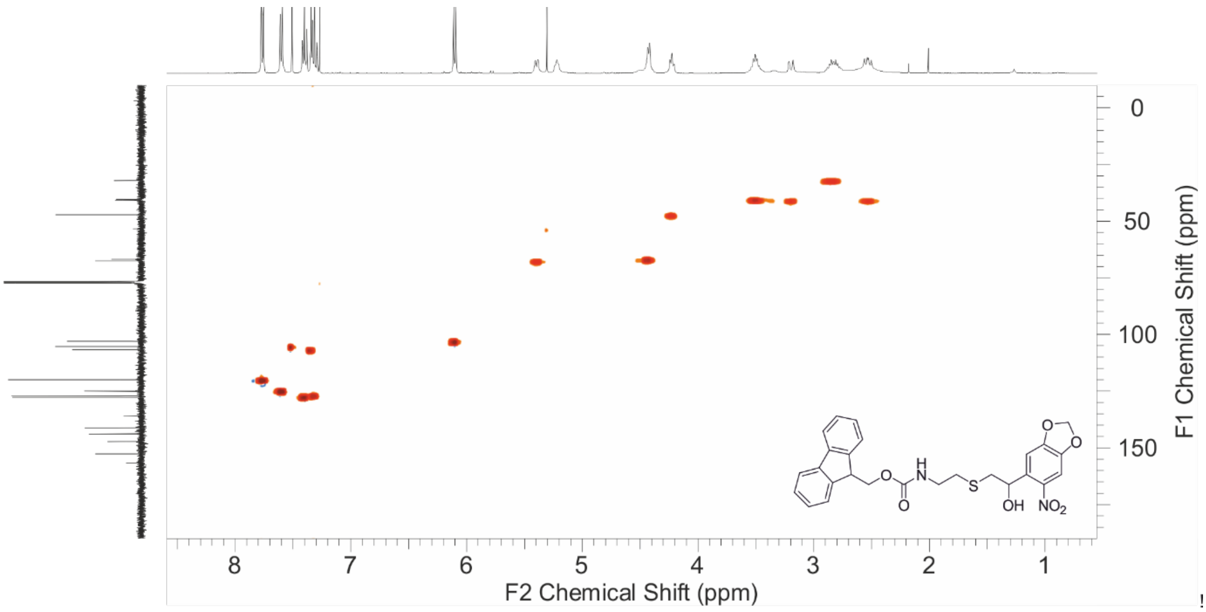

Methyl 4-(2-((2-((((9H-fluoren-9-yl)methoxy)carbonyl)amino)ethyl)thio)-1-(6-nitrobenzo[d][1,3]dioxol-5-yl)ethoxy)benzoate (5)

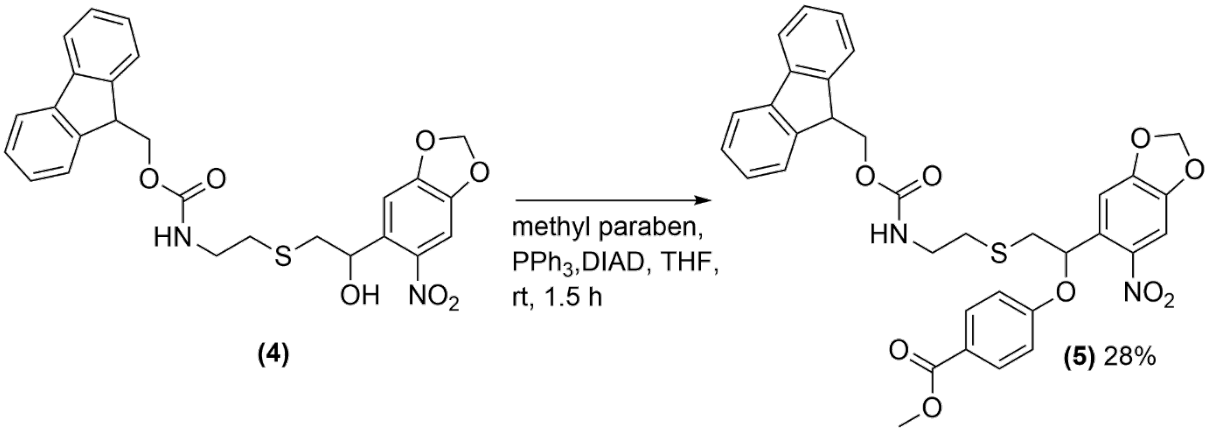

4 was coupled to methyl paraben in a Mitsunobu reaction. Due to the UV light sensitivity of the product the reaction vessel was protected from light with aluminium foil. Compound **4** (5.05 g, 9.9 mmol, 1.0 eq), methyl paraben (1.59 g, 10.4 mmol, 1.05 eq) and PPh_3 (2.74 g, 10.4 mmol, 1.05 eq) were dissolved in THF (4 ml) under argon atmosphere and sonicated in an ultrasonic bath (Bandelin electronic water bath sonicator, 320 W, 35 KHz) for three minutes, resulting in a brown viscous solution. DIAD (2.14 ml, 10.9 mmol, 1.1 eq) was added and the reaction mixture was sonicated for 1.5 h allowing the ultrasonic bath to heat up to 50 °C. The raw product was purified by flash column chromatography twice. First purification with CH/EA 2:1 yielded 7.7 g of a yellow-brown solid which was applied again to flash column chromatography using DCM/MeOH 100:1 as eluent. 1.12 g (18%) of a clean yellow solid were obtained and 5.19 g of an impure brownish. Repeated purification yielded further 630 mg (10%) of clean product.

R_f (CH/EA, 2:1) = 0.21

^{1}H-NMR (400 MHz, $CDCl_3$) δ [ppm] = 7.91 (d, J = 9.1 Hz, 2H), 7.76 (d, J = 7.6 Hz, 2H), 7.59 (d, J = 7.3 Hz, 2H), 7.59 (s, 1H), 7.40 (m, 2H), 7.31 (m, 2H), 7.09 (s, 1H), 6.83 (d, J = 8.8 Hz, 2H), 6.15 (dd, J = 6.7, 3.8 Hz, 1H), 6.08 (d, J = 8.3 Hz, 2H), 5.17 (m, 1H), 4.38 (d, J = 7.0 Hz, 2H), 4.20 (t, J = 7.0 Hz, 1H), 3.85 (s, 3H), 3. 46 (q, J = 5.9 Hz, 2H), 3.09 (m, 2H), 2.84 (m, 2H).

^{13}C-NMR (101 MHz, $CDCl_3$) δ [ppm] = 166.5, 160.7, 156.3, 153.0, 147.9, 143.8, 141.7, 141.3, 133.1, 131.7, 127.6, 127.0, 125.1, 123.7, 120.0, 115.2, 106.4, 105.7, 103.3, 75.9, 66.7, 51.9, 47.2, 40.2, 38.5, 33.1.

HR-ESI-MS: $[M+Na]^+_{(theor.)}$ = 665.15642, $[M+Na]^+_{(meas.)}$ = 665.15657.

^{1}H-NMR (400 MHz, $CDCl_3$) of **5**

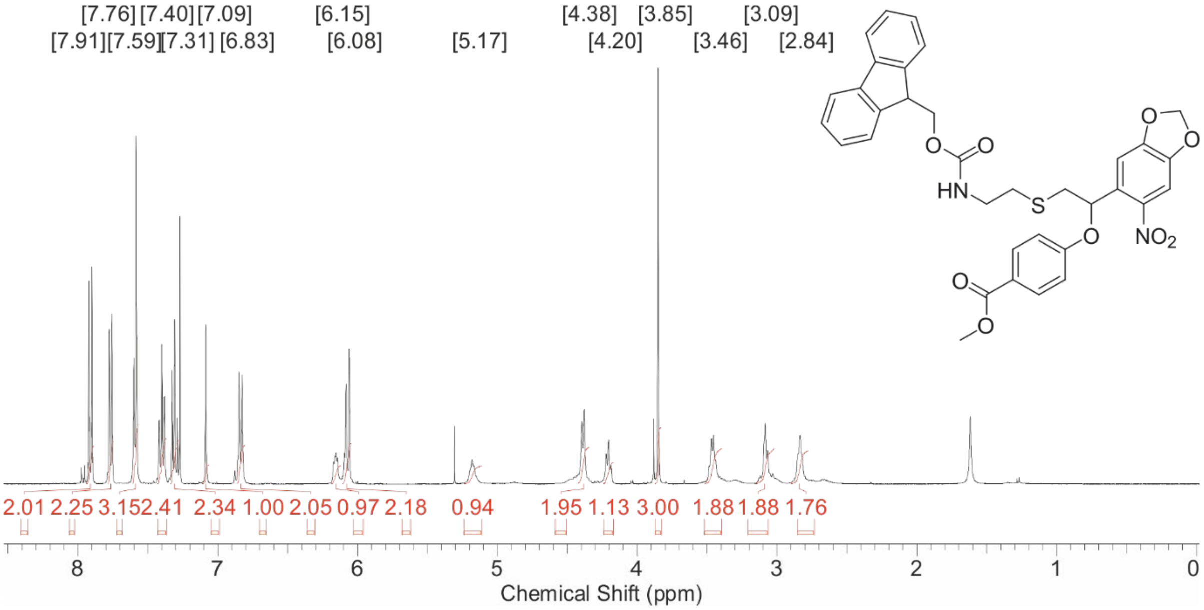

^{13}C-NMR (101 MHz, $CDCl_3$) of **5**

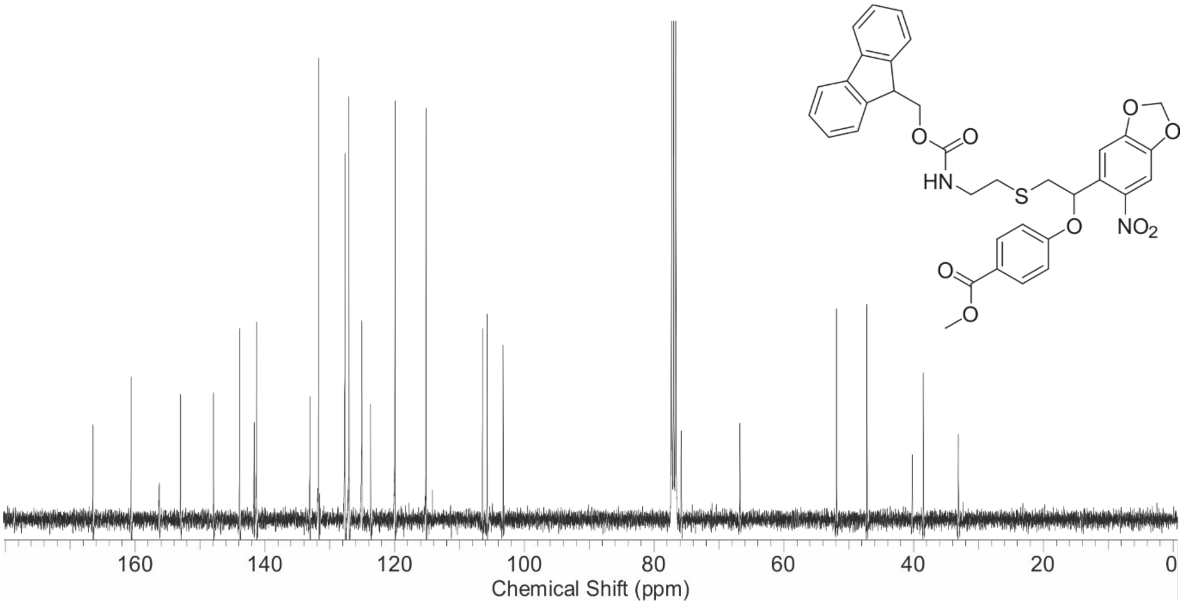

2D-NMR $^{1}H,^{1}H$-COSY of **5**

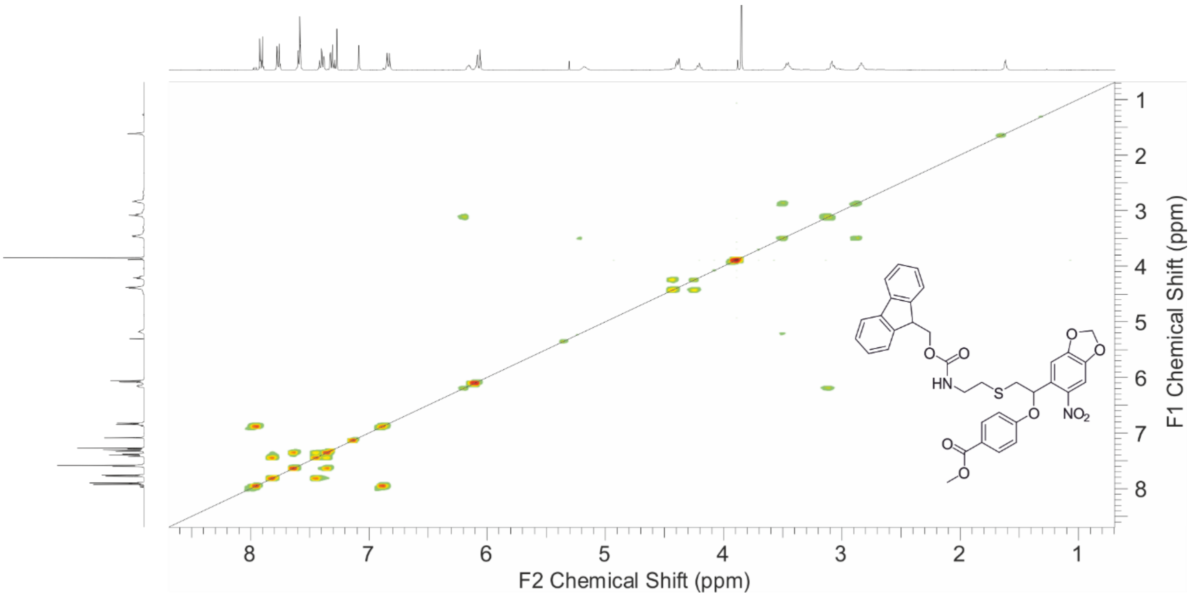

2D-NMR $^{1}H,^{13}C$-HSQC of **5**

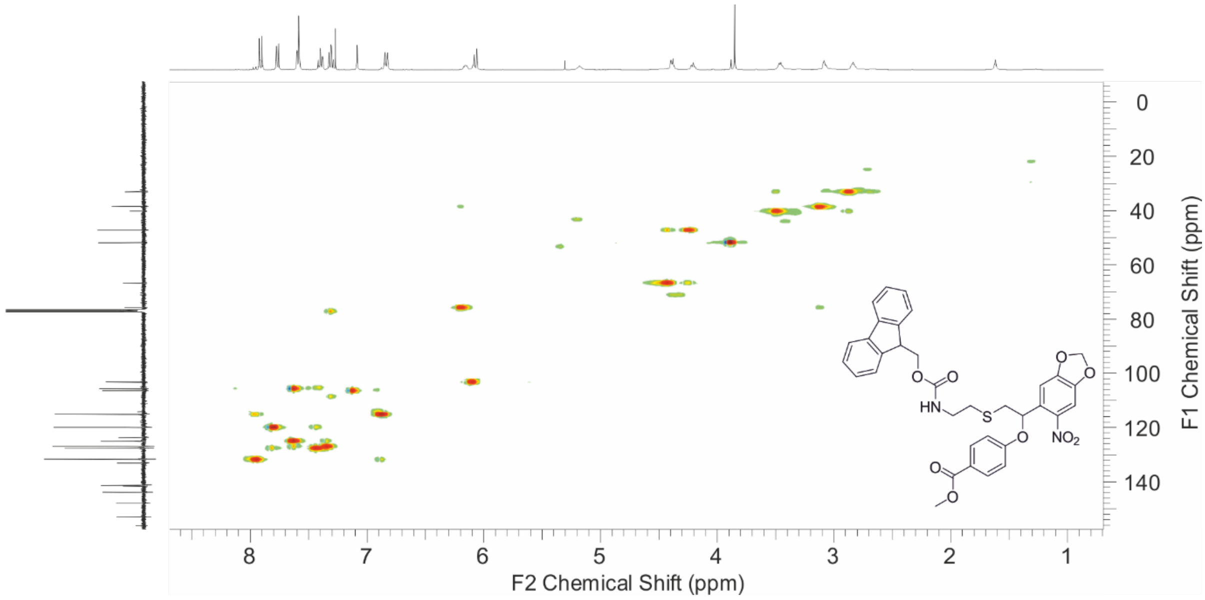

4-(2-((2-((((9H-Fluoren-9-yl)methoxy)carbonyl)amino)ethyl)thio)-1-(6-nitrobenzo[d][1,3]dioxol-5-yl)ethoxy)benzoic acid (6)

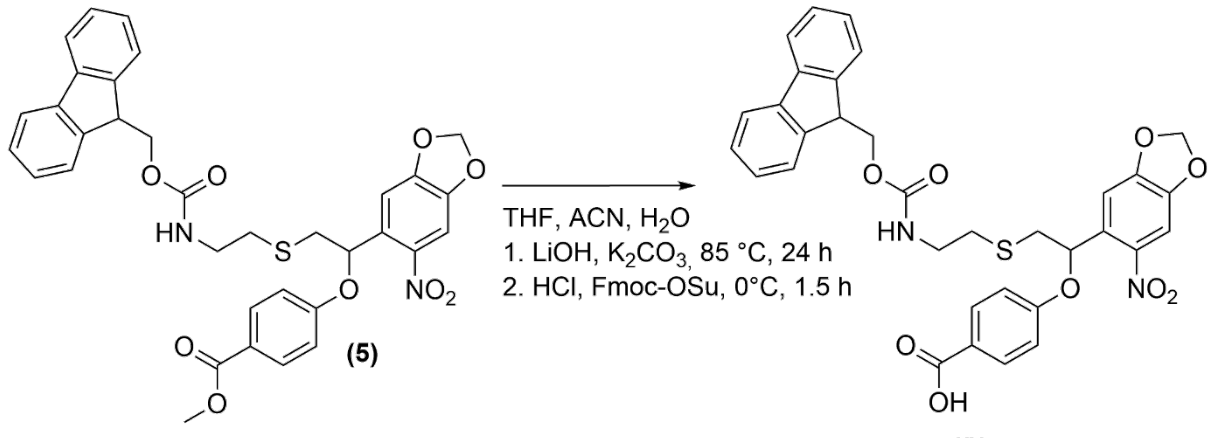

(6, Fmoc-^{UV}X-OH) 50%

5 (0.93 g, 1.5 mmol, 1.0 eq) was suspended in THF (40 ml) and stirred under argon atmosphere. LiOH (0.07 g, 5.8 mmol, 4.0 eq) in water (5.8 ml), K_2CO_3 (0.2 g, 2.9 mmol, 2.0 eq) and ACN (40 ml) were added, and the mixture was heated to 85 °C (reflux, 24 h). The saponification was stopped by adjusting the pH value to 8–9 with 1 M HCl under ice cooling. Since the Fmoc-group was cleaved under basic conditions, Fmoc-OSu (0.49 g, 1.5 mmol, 1.0 eq) was added at 0 °C. After stirring for 1.5 h at rt, the solvent was removed in vacuo. EA was added, and the organic layer was washed with brine and dried over Na_2SO_4. After evaporation of the solvent, the crude product was purified by flash column chromatography (CH/EA 2:3 + 0.1% AcOH) to yield 0.45 g (50%) clean product.

R_f (CH/EA, 2:3 + 0.1% AcOH) = 0.37

^{1}H-NMR (400 MHz, $CDCl_3$) δ [ppm] = 7.96 (d, *J* = 8.8 Hz, 2H), 7.76 (d, *J* = 7.6 Hz, 2H), 7.59 (d, *J* = 7.3 Hz, 2H), 7.59 (s, 1H), 7.40 (m, 2H), 7.31 (m, 2H), 7.08 (s, 1H), 6.85 (d, *J* = 8.7 Hz, 2H), 6.17 (dd, *J* = 6.6, 3.9 Hz, 1H), 6.08 (d, *J* = 8.2 Hz, 2H), 5,17 (m, 1H), 4.39 (d, *J* = 7.0 Hz, 2H), 4.20 (t, *J* = 6.9 Hz, 1H), 3. 46 (q, *J* = 5.9 Hz, 2H), 3.08 (m, 2H), 2.83 (m, 2H).

^{13}C-NMR (101 MHz, $CDCl_3$) δ [ppm] = 167.0, 161.2, 153.0, 147.9, 143.9, 141.6, 141.2, 133.0, 132.5, 127.7, 127.0, 125.1, 122.7, 120.0, 115.3, 106.4, 105.8, 103.4, 81.4, 75.9, 66.8, 47.2, 40.2, 38.4, 33.1.

HR-ESI-MS: $[M+Na]^+_{(theor.)}$ = 651.14077, $[M+Na]^+_{(meas.)}$ = 651.14100.

^{1}H-NMR (400 MHz, $CDCl_3$) of **6**

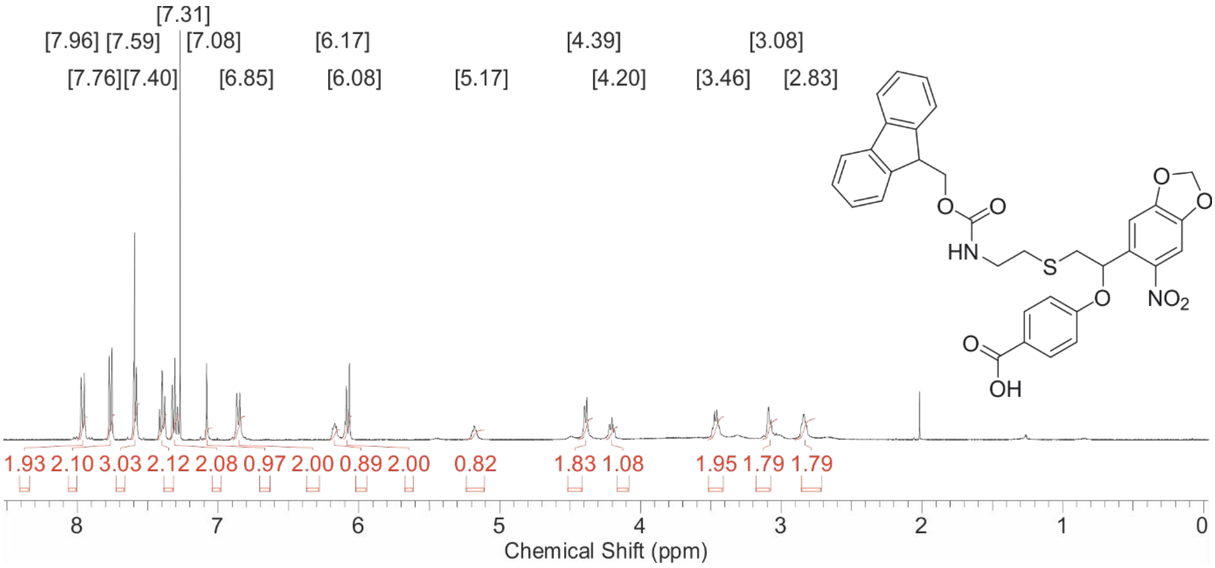

^{13}C-NMR (101 MHz, $CDCl_3$) of **6**

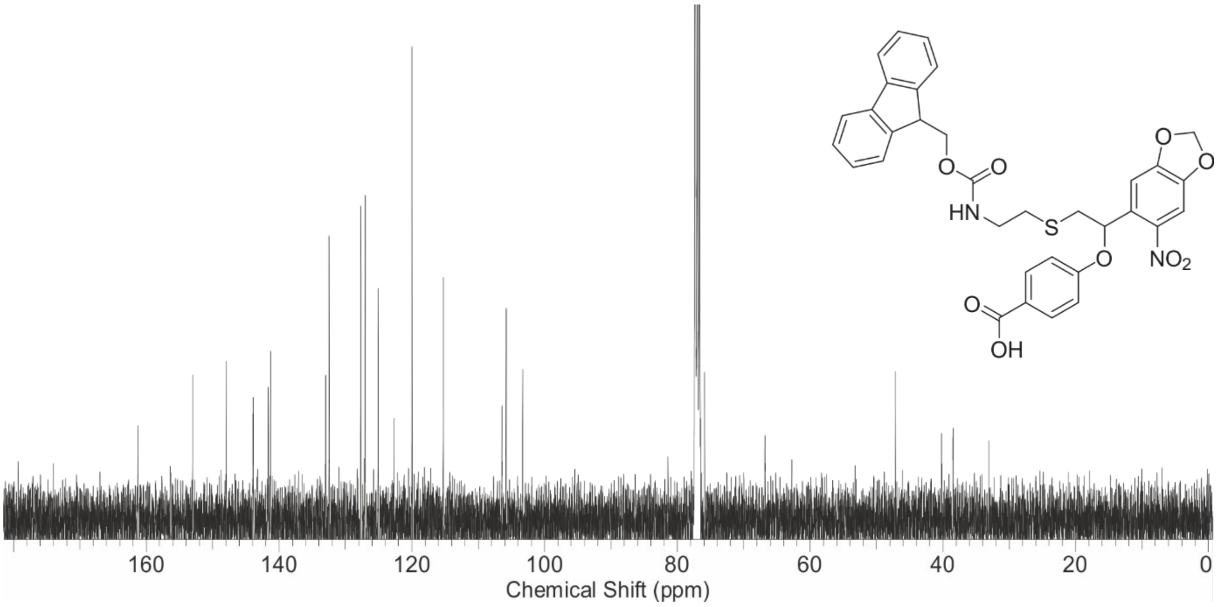

2D-NMR $^{1}H,^{1}H$-COSY of **6**

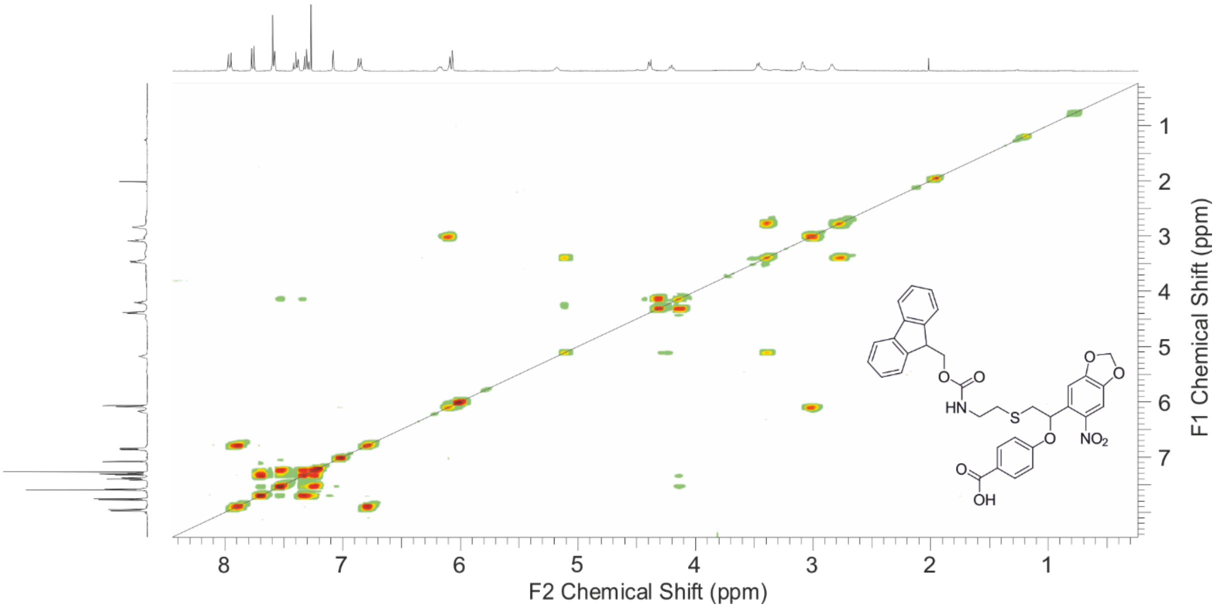

2D-NMR $^{1}H,^{13}C$-HSQC of **6**

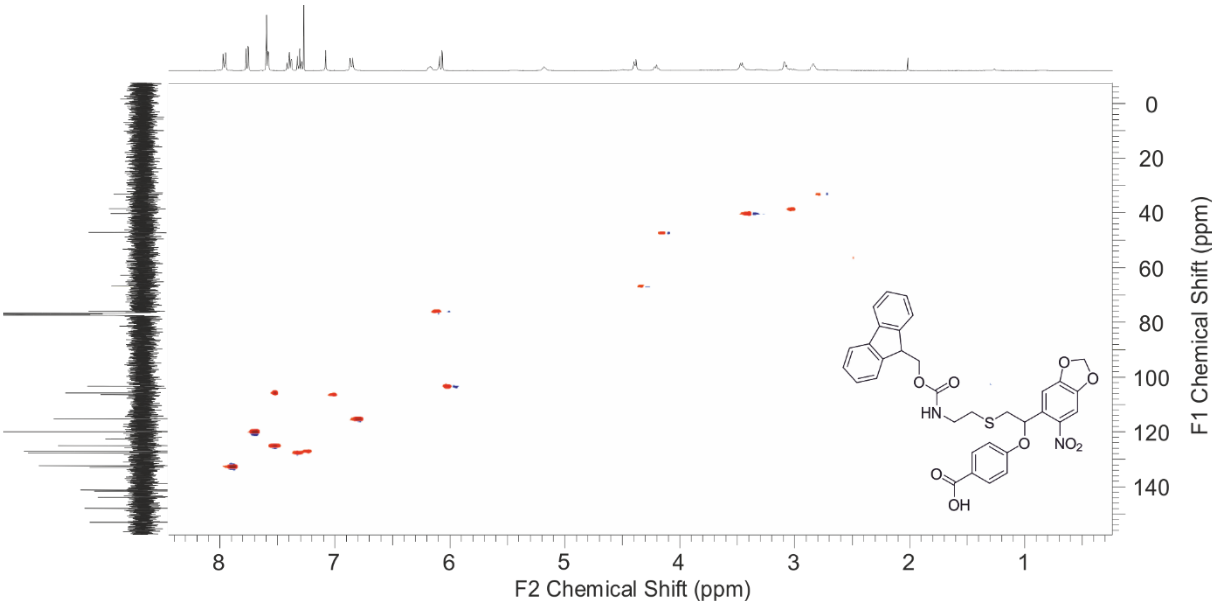

Solid-phase peptide synthesis (SPPS)

The general SPPS setup used here was recently described in detail. In short manual synthesis was performed in polypropylene/polyethylene syringes equipped with a polyether sulfone frit. Incubation was carried out on a VIBRAX VXR basic (IKA) shaker (1500 rpm) at room temperature. If not indicated otherwise the resin was washed after each coupling, capping or deprotection step with NMP/DCM 1:1 (3 times 5 ml), DCM (3 times 5 ml) and NMP (3 times 5 ml). If not indicated otherwise all amino acids were pre-activated with HBTU/HOBt for 5-15 min at room temperature before being added to the resin.

BG-OH and $^{N7\text{-}Npom}$BG-OH were synthesized as previously described.

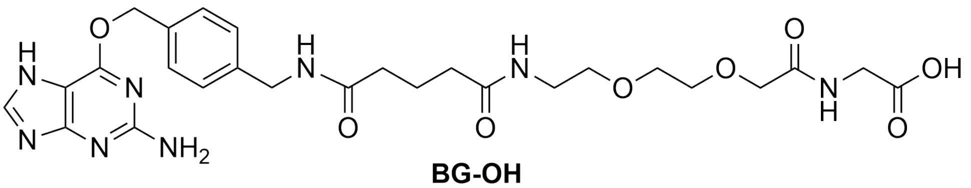

BG-OH

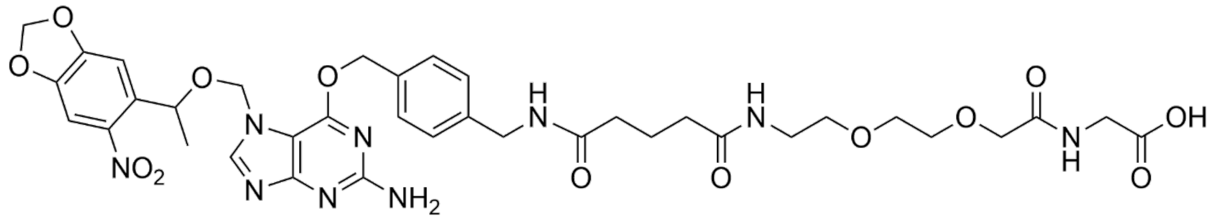

$^{N7\text{-}Npom}$BG-OH

BC-OH (7)

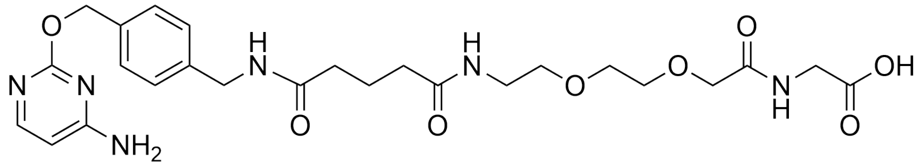

(7, BC-OH) 65%

BC-OH was obtained via solid-phase peptide synthesis as described for BG-OH starting from 413 mg (260 µmol, 1 eq) H-Gly-2-Cl-Trt resin and using 20 mg (87.0 µmol, 0.33 eq) BC-NH_2.[2] The resulting crude product was dissolved in 20 % buffer B, filtered, and purified via preparative HPLC, which yielded 31 mg (58.8 µmol, 65 %) BC-OH after lyophilization.

HR-ESI-MS: $[M+H]^+_{(theor.)}$ = 547.25109 $[M+H]^+_{(meas.)}$ = 547.25159.

BG-^{UV}X-OH (8)

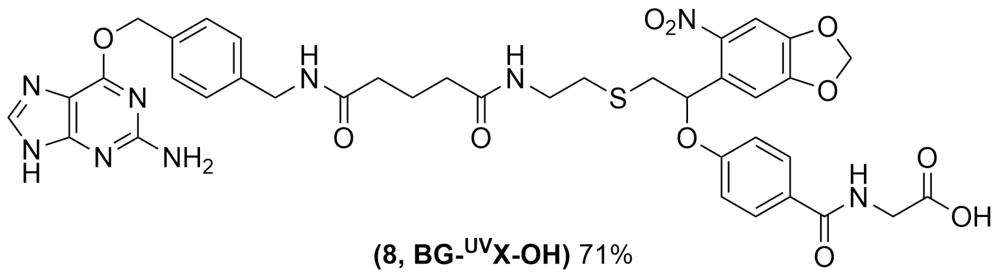

(8, BG-^{UV}X-OH) 71%

Fmoc-^{UV}X-OH (**6**, 140 mg, 170 µmol, 1.7 eq) was coupled to the H-Gly-2-Cl-Trt resin (Merck, 171 mg, 100 µmol, 1eq) with HBTU (57 mg, 150 µmol, 1.5 eq) and HOBt (31 mg, 200 µmol, 2 eq) in 1,4 ml NMP and 120 µl DIPEA for 1 hour. After washing the resin was capped two times with 6 ml acetic anhydride/DIPEA/NMP 1:1:10 for 10 min. The resin was deprotected using NMP/piperidine 5:1 (3 times 3 ml for 7 min). Glutaric anhydride (114 mg, 1 mmol, 10 eq) in 1,5 ml NMP and 150 µl DIPEA was coupled for 20 minutes. To activate the terminal carboxyl group, the linker was incubated with HBTU (380 mg, 1 mmol, 10 eq) and HOBt (230mg, 1.5 mmol, 1.5 eq) in 4.3 ml NMP and 0.7 ml DIPEA for 10 minutes. The resin was washed with dried NMP only (4 times). BG-NH_2 (20 mg, 75 µmol, 0.75 eq) in 3 ml DMF/DMSO 1:3 with 50 µl DIPEA was added and reacted for 3 hours. Afterwards the resin was washed with DCM/NMP, DCM, NMP and Et_2O (each 3 times, 5 ml). After swelling in DCM, the resin was cleaved in 15 ml DCM/HFIP/TFA 9:1:0.05 under continuous flow. The cleavage solution was dried in vacuo resulting in 126 mg of a yellow solid. The crude product was solved in eluent A and B. Preparative HPLC yielded 42.5 mg (51.8 µmol, 71%) BG-^{UV}X-OH.

HR-ESI-MS: $[M+H]^+_{(theor.)}$ = 830.25625 $[M+H]^+_{(meas.)}$ = 830.25666

$[M-H]^-_{(theor.)}$ = 828.24170 $[M-H]^-_{(meas.)}$ = 828.24108.

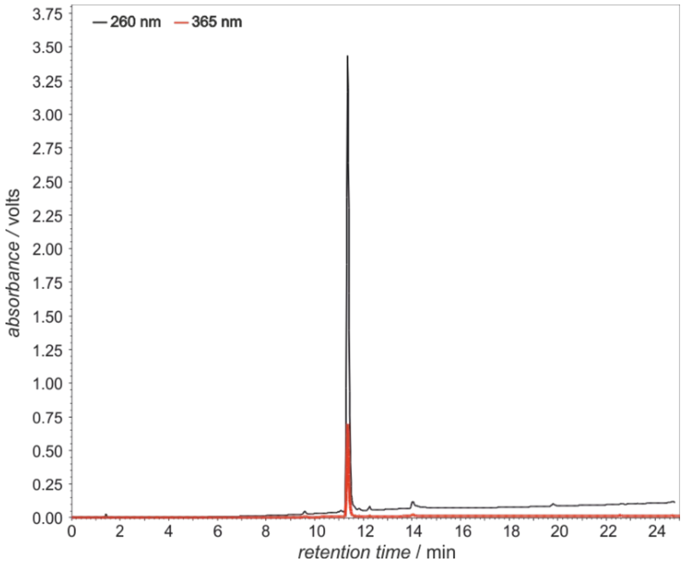

BC-^{UV}X-OH (9)

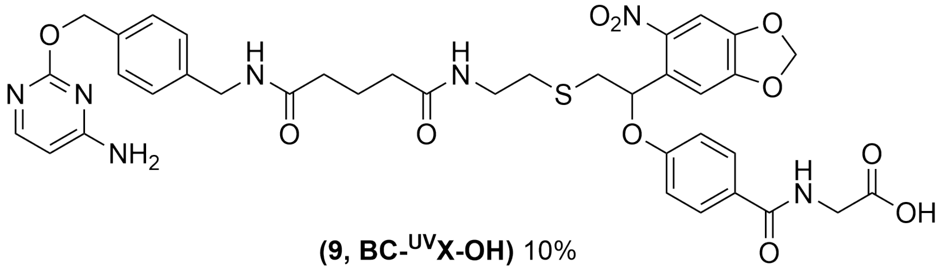

(9, BC-^{UV}X-OH) 10%

Fmoc-^{UV}X-OH (**6**, 70 mg, 85 µmol, 1.7 eq) was coupled to the H-Gly-2-Cl-Trt resin (Merck, 86.2 mg, 50 µmol, 1 eq) with HBTU (28.5 mg, 75 µmol, 1.5 eq) and HOBt (15.5 mg, 100 µmol, 2 eq) in 700 µl NMP and 60 µl DIPEA for 1 hour. The resin was deprotected using NMP/piperidine 5:1 (3 times 5 ml for 10 min). Next, glutaric anhydride (57 mg, 0.5 mmol, 10 eq) in 750 µl NMP and 75 µL DIPEA was coupled for 20 minutes. To activate the terminal carboxyl group, the linker was incubated with HBTU (190 mg, 0.5 mmol, 10 eq) and HOBt (115 mg, 0.75 mmol, 1.5 eq) in 2.2 ml NMP and 350 µl DIPEA for 10 minutes. The resin was washed with dried NMP only (4 times). BC-NH_2 (8.6 mg, 37.5 µmol, 0.75 eq) in 500 µl NMP and 125 µl DIPEA was added and reacted for 4 hours. Test cleavage showed a low coupling efficiency. Therefore, the resin was activated again with EDCI (500 µmol, 10 eq) and NHS (750 µmol, 15 eq) in NMP/DIPEA 17:3. BC-NH_2 (8.6 mg, 37.5 µmol, 0.75 eq) was added and incubated for another 3 hours. Afterwards the resin was washed with DCM/NMP, DCM, NMP and Et_2O (each 3 times, 5 ml). After swelling in DCM, the resin was cleaved with 30 ml DCM/HFIP/TFA 9:1:0.05 under continuous flow. The crude product was dried in vacuo and solved in eluent A and B for HPLC. Preparative HPLC yielded 3.0 mg (3.8 µmol, 10%) BC-^{UV}X-OH.

HR-ESI-MS: $[M+H]^+_{(theor.)}$ = 790.25010 $[M+H]^+_{(meas.)}$ = 790.24965.

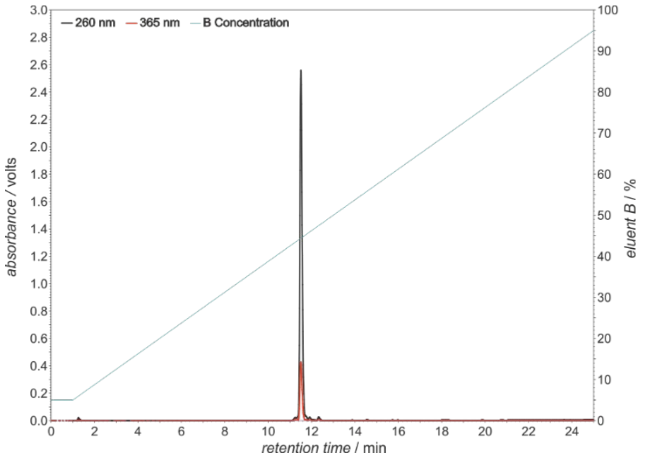

Note: We have recently published an optimized protocol for the synthesis of $^{N7\text{-}Npom}$BG-OH and BG-OH linker.[5] There we recommend an activation of the glutaric anhydride on the resin using pentafluorphenyl-trifluoracetat. To our experience this yields higher coupling efficiency and allows to store the activated resin for at least 3 months at -20°C. However, we do not recommend applying this protocol for linker containing the Fmoc-^{UV}X-OH building block since it results in strong formation of a side product with an oxidized sulfur (data not shown).

(4-Hydroxybenzoyl)glycine (10)

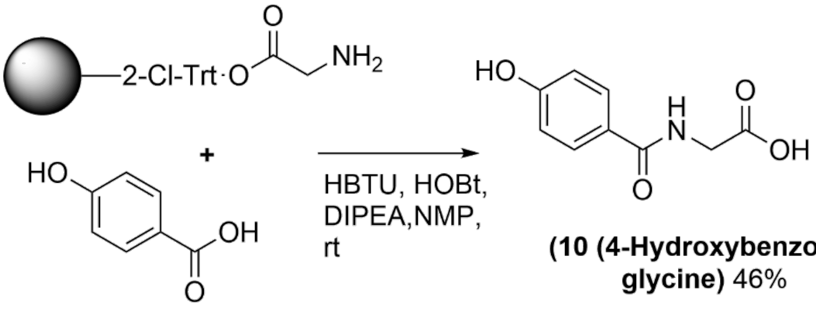

(10 (4-Hydroxybenzoyl)-glycine) 46%

H-Gly-2-Cl-Trt resin (Merck, 71 mg, 45 µmol, 1.0 eq) was swelled in NMP (2 ml) for 10 min. 4-hydroxybenzoic acid (25 mg, 0.18 mmol, 4.0 eq), was preactivated with HBTU (83 mg, 0.18 mmol, 4.0 eq) and HOBt (33 mg, 0.18 mmol, 4.0 eq) in 2 ml NMP/DIPEA 8:1 for 5 min. The preactivation was added to the resin and shaken for 30 min. The resin was washed with DCM/NMP 1:1 (4x), DCM (4x), NMP (4x) and DCM (4x). Cleavage was performed with DCM + 0.5% TFA (15 ml). The cleavage solution was evaporated, and the crude product applied to preparative HPLC. 4 mg (46%) of a white solid were obtained.

^{1}H-NMR (250 MHz, DMSO-d$_6$) δ [ppm] = 9.97 (br s, 1H), 8.54 (t, *J* = 5.9 Hz, 1H), 7.73 (d, *J* = 8.8 Hz, 2H), 6.79 (d, *J* = 8.8 Hz, 2H), 3.89 (d, *J* = 5.9 Hz, 2H).

LC-MS: $[M+H]^+$ = 196.1, $[2M+H]^+$ = 391.1, $[M-H]^-$ = 194.1, $[2M-H]^-$ = 389.1.

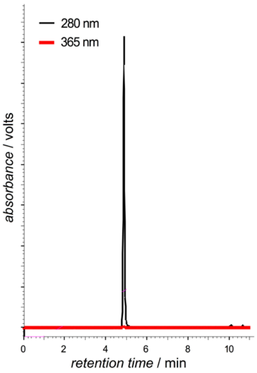

1H-NMR (250 MHz, DMSO-d$_6$) of **10**

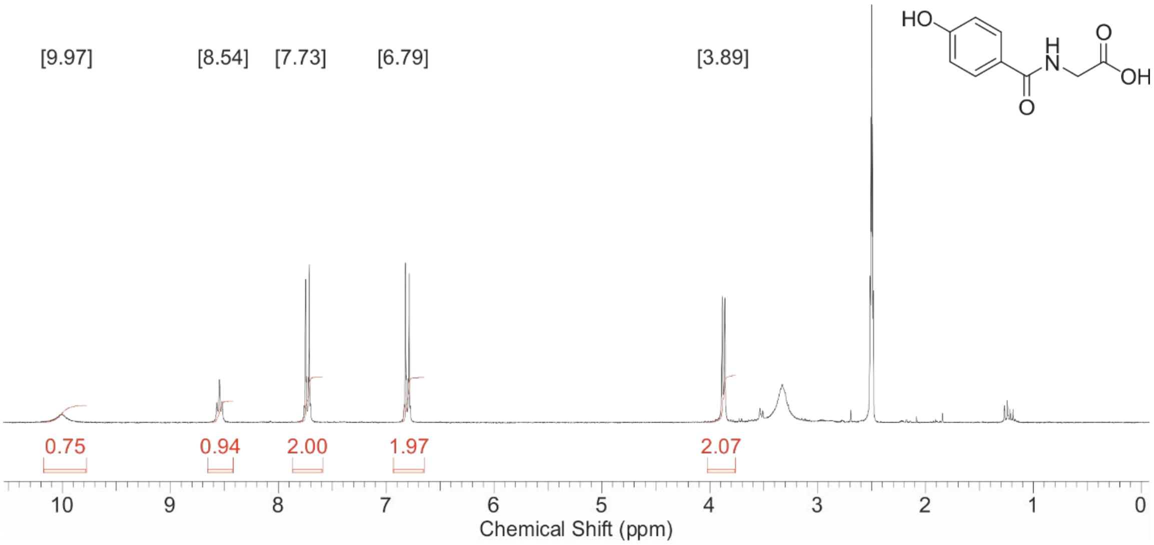

BG-L-Lys(NH_2)-OH and $^{N7\text{-}Npom}$BG-L-Lys(NH_2)-OH (11,12)

The SPPS of the linker for BG-Lys(NH_2)-OH and $^{N7\text{-}Npom}$BG-Lys(NH_2)-OH was carried out in parallel until the final coupling step. Of 0.71 mg of H-Gly-2-Cl-Trt-resin (450 µmol) initially used, one quarter (112.5 µmol) was used for the synthesis BG-L-Lys(NH_2)-OH and one quarter (112.5 µmol) was used for the synthesis $^{N7\text{-}Npom}$BG-L-Lys(NH_2)-OH. The remaining resin was stored at -20°C after pentafluorphenyl activation and used for later syntheses.

Fmoc-L-Lys(Mtt)-OH (845 mg, 1.35 mmol, 3 eq) was coupled to the H-Gly-2-Cl-Trt resin (Merck, 705 mg, 450 µmol, 1 eq) with HBTU (455 mg, 1.2 mmol, 2.7 eq) and HOBt (182 mg, 1.35 mmol, 3 eq) in 6 ml NMP and 1.5 ml DIPEA for 1 hour. After washing the resin was capped two times with 12 ml acetic anhydride/DIPEA/NMP 1:1:10 for 10 min. The resin was deprotected using NMP/piperidine 5:1 (3 times 5 ml for 10 min) and Fmoc-AEEA-OH (520 mg, 1.35 mmol, 3 eq) was coupled with HBTU (455 mg, 1.2 mmol, 2.7 eq) and HOBt (182 mg, 1.35 mmol, 1 eq) in 6 ml DMSO and 1.5 ml DIPEA for 1 hour. The resin was capped and deprotected as described above and glutaric anhydride (500 mg, 4.4 mmol, 10 eq) in 10 ml NMP/DIPEA 5:1 was coupled for 30 min. An additional washing step with 0.1% NaOH in Dioxan/H_2O 1:1 was performed, and the resin was washed again to remove remaining H_2O. To activate the terminal carboxyl group, pentafluorphenyl-trifluoracetat (Pfp-TFA, 373 µL, 2.2 mmol, 5 eq) in 5 ml pyridine/DCM 1:1 was incubated with the resin two times (10 min + 20 min). The resin was washed as described above and followed by additional washing with DCM (3 times). The resin was dried in vacuo and split into 4 equal portions according to weight.

BG-L-Lys(NH_2)-OH (11)

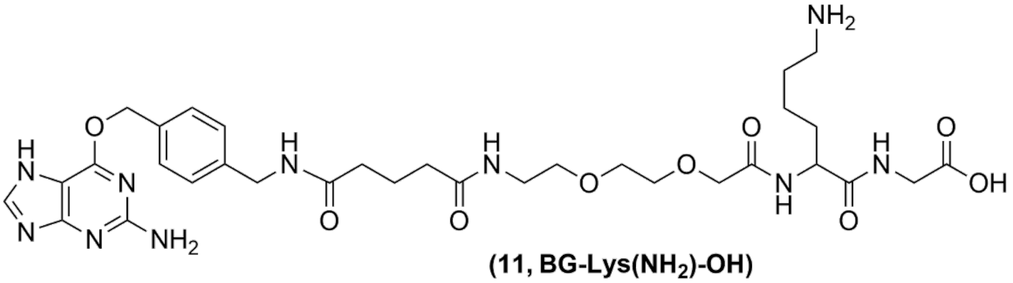

(11, BG-Lys(NH_2)-OH)

For synthesis of BG-L-Lys(NH_2)-OH, BG-NH_2 (10 mg, 37.5 µmol, 0.33 eq) was solved in 3 ml NMP/DMSO 1:3 with 50 µl DIPEA and incubated with Pfp-glutaric anhydride-AEEA-L-Lys(Mtt)-Gly-2-Cl-Trt resin (112.5 µmol, 1 eq) for 18 hours at room temperature. The resin was washed with DCM/NMP, DCM, NMP and Et_2O (each 3 times, 5 ml). After swelling in DCM, the resin was cleaved with 30 ml DCM/HFIP/TIPS/TFA 9:1:0.05:0.1 under continuous flow. The crude product was dried in vacuo and purified by preparative HPLC.

HR-ESI-MS: $[M+H]^+_{(theor.)}$ = 715.35220, $[M+H]^+_{(meas.)}$ = 715.35235.

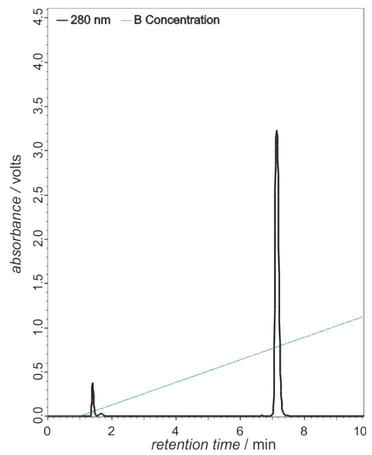

$^{N7\text{-}Npom}$BG-L-Lys(NH_2)-OH (12)

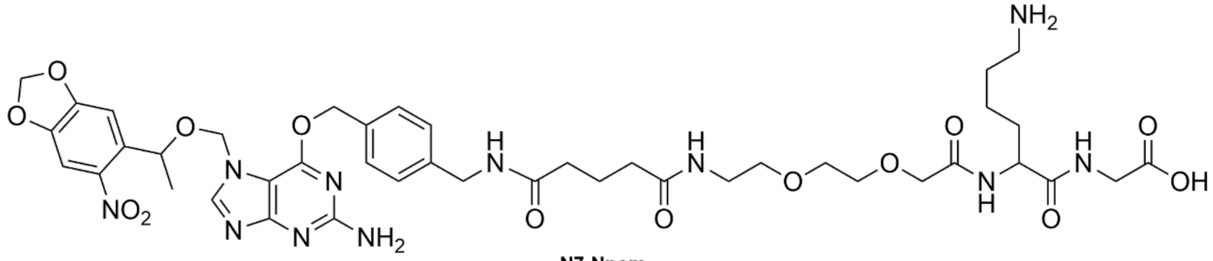

(12, $^{N7\text{-}Npom}$BG-Lys(NH_2)-OH) 50%

For synthesis of $^{N7\text{-}Npom}$BG-L-Lys(NH_2)-OH, $^{N7\text{-}Npom}$BG-NH_2 (16 mg, 33 µmol, 0.3 eq) was solved in 1.5 ml NMP/DMSO/DIPEA 100:15:8 and incubated with Pfp-glutaric anhydride-AEEA-L-Lys(Mtt)-Gly-2-Cl-Trt resin (112.5 µmol) for 18 hours at room temperature. The resin was washed with DCM/NMP, DCM, NMP and Et_2O (each 3 times, 5 ml). After swelling in DCM, the resin was cleaved with 30 ml DCM/HFIP/TIPS/TFA 9:1:0.05:0.1 under continuous flow. The crude product was dried in vacuo and purified by preparative HPLC, yielding 8.5 mg (**12**, 9 µmol, 27%).

HR-ESI-MS: $[M+H]^+_{(theor.)}$ = 938.40027, $[M+H]^+_{(meas.)}$ = 938.39942.

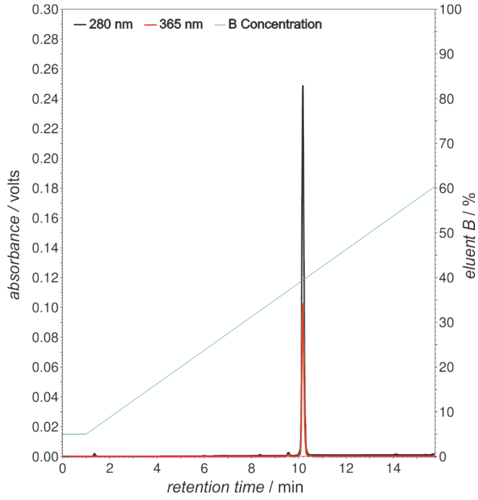

Synthesis of bifunctional linker

For the synthesis of the bifunctional linkers BC-OH or BC-^{UV}X-OH were activated with N,N'-diisopropylcarbodiimide (DIC) and N-hydroxysuccinimide (NHS) to form the corresponding NHS ester. The NHS esters were used in situ to react either with BG-Lys(NH_2)-OH or $^{N7\text{-}Npom}$Bg-Lys(NH_2)-OH.

For preactivation the following solutions were freshly prepared and used within a day:

- DIC in DMSO (34 mg/ml, 270 mM)
- NHS in DMSO (53.1 mg/ml, 450 mM)
- DIPEA/DMSO 1:20
- All linker molecules were solved in DMSO to a concentration of 60 mM

The amount of BC-OH or BC-^{UV}X-OH linker activated for each synthesis is listed for the respective molecule below. Equal volumes of the above mentioned four solutions were mixed and the reaction was carried out for 15-18 hours at 40°C on a shaker at 900 rpm. The solution was then freeze dried overnight using a Christ Alpha 2-4 LDplus lyophilizer to remove solvent and excess DIC. The resulting oil was solved in 140 µl DMSO and 100 µl $NaHCO_3$ (0.2 M in H_2O), and added to the indicated amount of BG-Lys(NH_2)-OH or $^{N7\text{-}Npom}$Bg-Lys(NH_2)-OH linker.

BG/BC-OH linker (13)

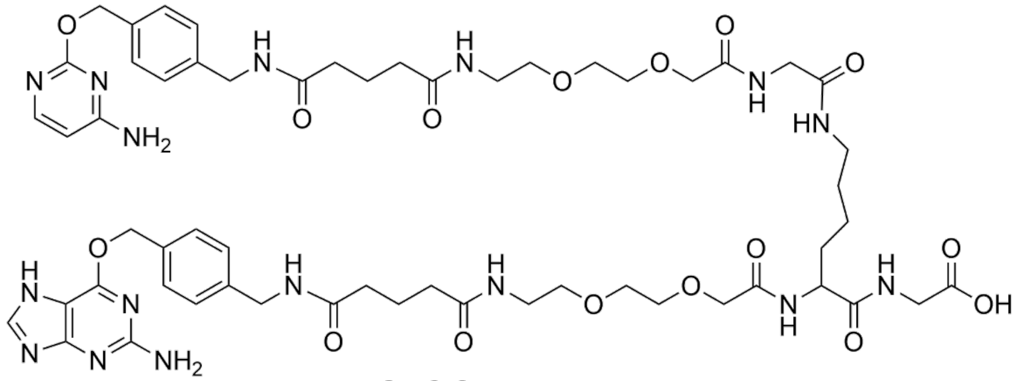

(13, BG/BC-OH) 33%

BC-OH linker (**7**, 60 mM in DMSO, 60 µl, 3.6 µmol, 1 eq) was preactivated and reacted with BG-L-Lys(NH_2)-OH linker (**11**, 60 mM in DMSO, 90 µl, 5.4 µmol, 1.5 eq) for 35 min. The reaction was stopped by the addition of 2.5 ml of HPLC eluent A/B 5:1. The linker was purified by preparative HPLC, yielding 1.5 mg BG/BC-OH linker (1.2 µmol, 33%).

LC-MS: $[M+2H]^{2+}_{(theor.)}$ = 621.29 $[M+2H]^{2+}_{(meas.)}$ = 622.40, $[M+3H]^{3+}_{(meas.)}$ = 415.25, $[M+4H]^{4+}_{(meas.)}$ = 311.65.

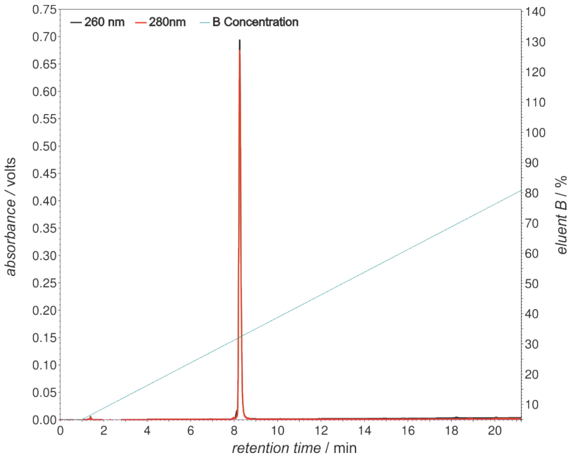

$^{N7\text{-}Npom}$BG/BC-OH linker (14)

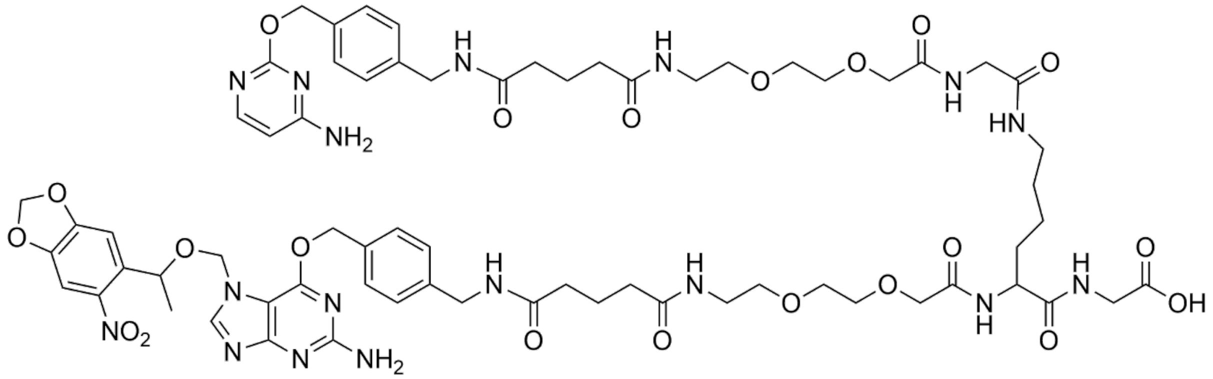

(14, $^{N7\text{-}Npom}$BG/BC-OH) 31%

BC-OH linker (**7**, 60 mM in DMSO, 60 µl, 3.6 µmol, 1 eq) was preactivated and reacted with $^{N7\text{-}Npom}$BG-L-Lys(NH_2)-OH linker (**12**, 60 mM in DMSO, 65 µl, 3.9 µmol, 1.08 eq) for 30 min. The reaction was stopped by the addition of 2.5 ml of HPLC eluent A/B 5:1. The linker was purified by preparative HPLC, yielding 1.6 mg $^{N7\text{-}Npom}$BG/BC-OH linker (1.1 µmol, 31%).

HR-ESI-MS: $[M+H]^+_{(theor.)}$ = 1466.63352, $[M+H]^+_{(meas.)}$ = 1466.63192
HR-ESI-MS: $[M+2H]^{2+}_{(theor.)}$ = 733.82040, $[M+2H]^{2+}_{(meas.)}$ = 733.82055.

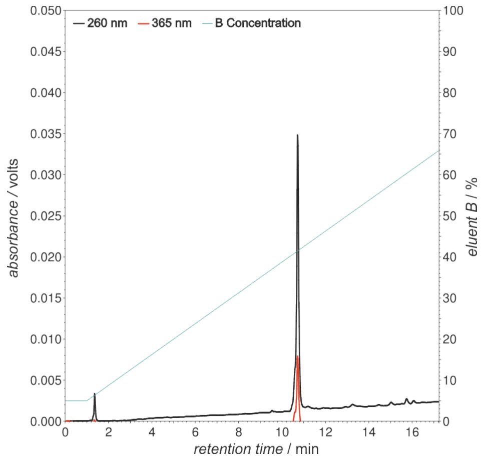

$^{N7\text{-}Npom}$BG/BC-^{UV}X-OH linker (15)

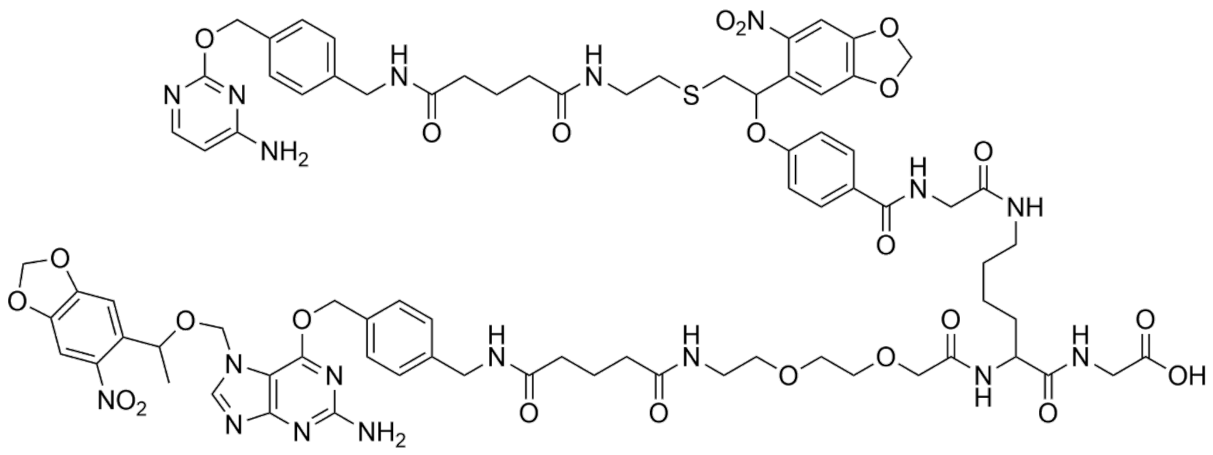

(15, $^{N7\text{-}Npom}$BG/BC-^{UV}X-OH) 11%

BC-^{UV}X-OH linker (**9**, 60 mM in DMSO, 60 µl, 3.6 µmol, 1 eq) was preactivated and reacted with $^{N7\text{-}Npom}$BG-L-Lys(NH_2)-OH linker (**12**, 60 mM in DMSO, 65 µl, 3.9 µmol, 1.08 eq) for 30 min. The reaction was stopped by the addition of 2.5 ml of HPLC eluent A/B 5:1. The linker was purified by preparative HPLC, yielding 0.7 mg $^{N7\text{-}Npom}$BG/BC-^{UV}X-OH linker (0.41 µmol, 11%).

LC-MS: $[M+2H]^{2+}_{(theor.)}$ = 854.31 $[M+2H]^{2+}$ = 855.75, $[M+3H]^{3+}$ = 570.80, $[M+4H]^{4+}$ = 428.25.

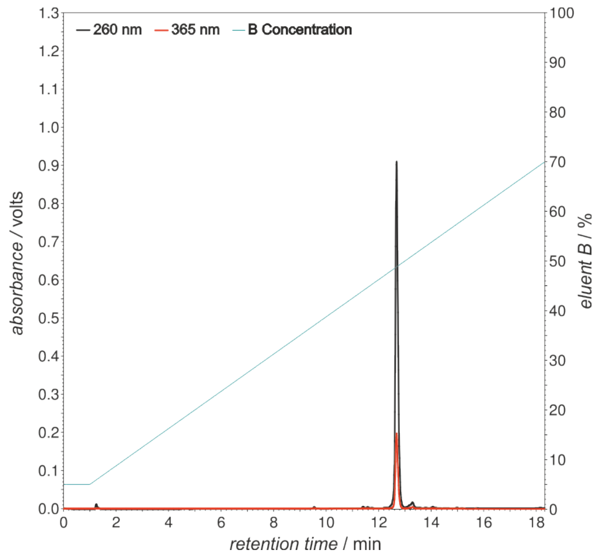

Synthesis of BG-Atto488 and BG-^{UV}X-Atto488

For the synthesis of the fluorophore conjugates BG-OH and BG-^{UV}X-OH were activated with 1-ethyl-3-(3-dimethylaminopropyl)carbodiimide (EDCI) and N-hydroxysuccinimide (NHS) to form the corresponding NHS ester. The NHS esters were used in situ to react with Atto488-amine (Atto-tec GmbH, Germany).

For preactivation the following solutions were freshly prepared and used within a day:
- EDCI·HCl in DMSO (52.2 mg/ml, 270 mM)
- NHS in DMSO (53.1 mg/ml, 460 mM)
- DIPEA/DMSO 1:20
- All linker molecules were solved in DMSO to a concentration of 60 mM

The amount of BG-OH and BG-^{UV}X-OH linker activated for each synthesis is listed for the respective molecule below. Equal volumes of the above mentioned four solutions were mixed and the reaction was carried out for 15-18 hours at 37°C on a shaker (900 rpm).

BG-Atto488 (16)

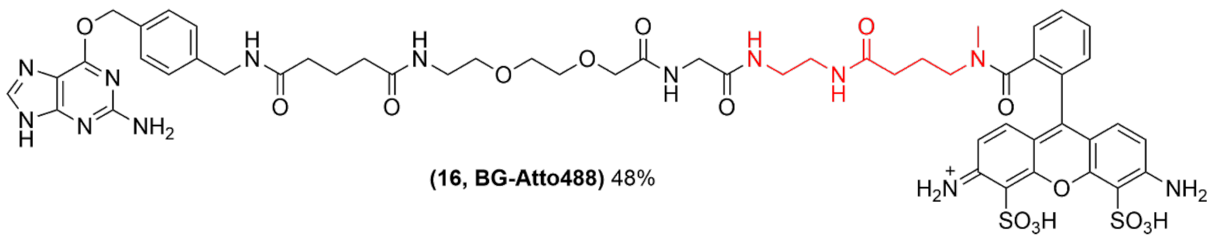

(16, BG-Atto488) 48%

BG-OH linker (60 mM in DMSO, 20 µl, 1.2 µmol, 4 eq) was preactivated and reacted with Atto488-amine (11.6 mM in DMSO, 25 µl, 0.29 µmol, 1 eq) for 60 min at 37°C on a shaker at 900 rpm. The reaction was stopped by the addition of 2.5 ml of HPLC eluent A and followed by HPLC purification yielding 170 µg BG-Atto488 (0.14 µmol, 48 %). The yield was determined by solving the product in DMSO, dilution in phosphate buffered saline (PBS) and measurement of the absorbance at 500 nm ($\varepsilon_{500\ nm}$ = 90.000 $M^{-1}cm^{-1}$). *Note: the full structure of Atto488-amine is not available from the supplier. The substructure marked in red within the structural formula is therefore not confirmed but fits to the HR-MS analysis.*

HR-ESI-MS: $[M+2H]^{2+}_{(theor.)}$ = 600.69731, $[M+2H]^{2+}_{(meas.)}$ = 600.69664.

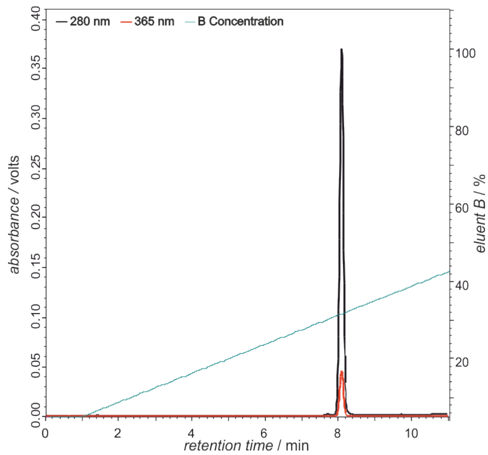

BG-^{UV}X-Atto488 (17)

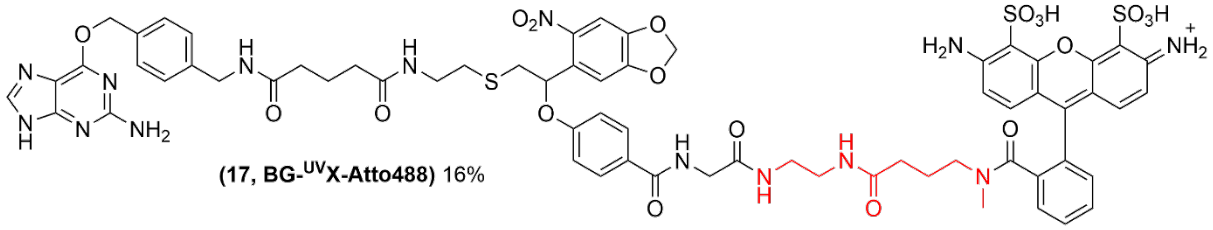

BG-^{UV}X-OH linker (**8**, 60 mM in DMSO, 30 µl, 1.8 µmol, 4.4 eq) was preactivated and reacted with Atto488-amine (11.6 mM in DMSO, 35 µl, 0.41 µmol, 1 eq) for 60 min at 37˚C on a shaker at 900 rpm. The reaction was stopped by the addition of 2.5 ml of HPLC eluent A and followed by HPLC purification yielding 91 µg BG-^{UV}X-Atto488 (63 nmol, 16%). The yield was determined by solving the product in DMSO, dilution in phosphate buffered saline (PBS) and measurement of the absorbance at 500 nm ($\varepsilon_{500\ nm}$ = 90.000 $M^{-1}cm^{-1}$). *Note: the full structure of Atto488-amine is not available from the supplier. The substructure marked in red within the structural formula is therefore not confirmed but fits to the HR-MS analysis.*

HR-ESI-MS: $[M+2H]^{2+}_{(theor.)}$ = 722.19682, $[M+2H]^{2+}_{(meas.)}$ = 722.19613.

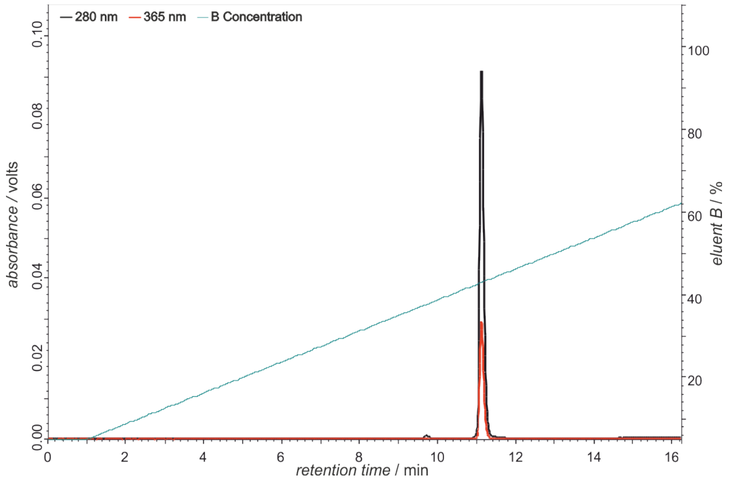

guideRNA synthesis

NH-Stop-66 guideRNA was obtained from Eurofins in HPLC-purified quality carrying a 5´-C6-aminolinker. NH-Y701C and NH-T288A were obtained from Biospring (Frankfurt, Germany) in HPLC-purified quality carrying a 5´-C6-aminolinker. OMe modified poly(U) was obtained from Sigma Aldrich in desalted quality carrying a 5´-C6-aminolinker and OMe/LNA modified poly(U) was obtained from Eurogentec in desalted quality carrying a 5´-C6-aminolinker and a 3'-C7-aminolinker. NH-Y701C and NH-T288A guideRNAs were solved in RNase free water to a concentration of 6 µg/µl. NH-Stop66 and NH-poly(U) were precipitated with 0.1 volumes of 3 M NaCl and 3 volumes of 100% EtOH, washed with 70% EtOH and dissolved in RNase free water (6 µg/µl) prior to coupling. We recently described the guideRNA synthesis in detail. In short the respective linker (BG-OH, BC-OH, $^{N7\text{-}Npom}$BG-OH, BG-^{UV}X-OH, BC-^{UV}X-OH, BG/BC-

OH, $^{N7\text{-}Npom}$BG/BC-OH or $^{N7\text{-}Npom}$BG/BC-^{UV}X-OH) was activated with DIC, NHS and DIPEA in DMSO according to the protocol for the synthesis of bifunctional linker described above. For all linker containing photo-sensitive moieties the reaction was performed protected from light. The resulting NHS ester was then reacted with the corresponding NH_2-guideRNA in DMSO/H_2O/DIPEA 66:33:1 and purified on a 20% UREA-PAGE. For all linker containing photo-sensitive moieties, the UREA-PAGE was performed in the dark. While the main part of the gel was protected with aluminum foil one lane containing ca. 10% of the crude guideRNA was analyzed on a TLC plate under 254 nm UV light. The migration of NH_2-guideRNA and modified gRNA were noted, and the analyzed lane was discarded. The region corresponding to migration of the modified gRNA was cut out from the remaining lanes and was transferred to an amber reaction tube. guideRNAs were extracted from the gel slices by shaking overnight at 4°C with nuclease free water and precipitated with 0.1 volumes sodium acetate (NaOAc, 3 M) and 3 volumes of EtOH 100% (incubation at -20°C for >4 h). After centrifugation (17,000 g, -4°C, >45 min) the guideRNA pellet was washed with 70% EtOH and dissolved in nuclease free water. Concentrations were determined photometrically by absorption at 260 nm. The extinction coefficients were estimated from the $\varepsilon_{260\ nm}$ provided from the commercial supplier and the sum of the following moieties incorporated into the terminal modification: BG ($\varepsilon_{260\ nm}$ ~ 2.5 $mM^{-1}cm^{-1}$), BC ($\varepsilon_{260\ nm}$ ~ 4.2 $mM^{-1}cm^{-1}$), $^{N7\text{-}Npom}$BG ($\varepsilon_{260\ nm}$ ~6.5 $mM^{-1}cm^{-1}$) and ^{UV}X ($\varepsilon_{260\ nm}$ ~ 23 $mM^{-1}cm^{-1}$).

The synthesis BG-guideRNAs and $^{N7\text{-}Npom}$BG-guideRNAs has been well established in previous publications. By our experience the same protocol also achieves good yields of BG-^{UV}X and bifunctional guideRNAs. Shown below on the left panel is the image of an exemplary preparative UREA-PAGE of the crude BG-^{UV}X-guideRNA (BG-^{UV}X-Stop66 used in figure 1d). Two guideRNAs-containing bands are observed with the faster migrating band corresponding to unreacted NH_2-guideRNA followed by the BG-^{UV}X-guideRNA. Scalpel cuts indicate the areas of the UREA- PAGE that were excised for purification and analysis. Excess of BG-^{UV}X-OH can be easily identified by its absorbance spectrum after extraction (see figure S1a). On the right panel, the MALDI-MS spectrum of the pure BG-^{UV}X-Stop66 guideRNA obtained from this synthesis is shown. The two highest peaks correlate to the expected single ionized ($[M–H]^{-}$ = 7360) and double ionized ($[M–2H]^{2-}$ = 3680) product. No signal corresponding to NH-guideRNA (Mw = 6550 g/mol) was observed.

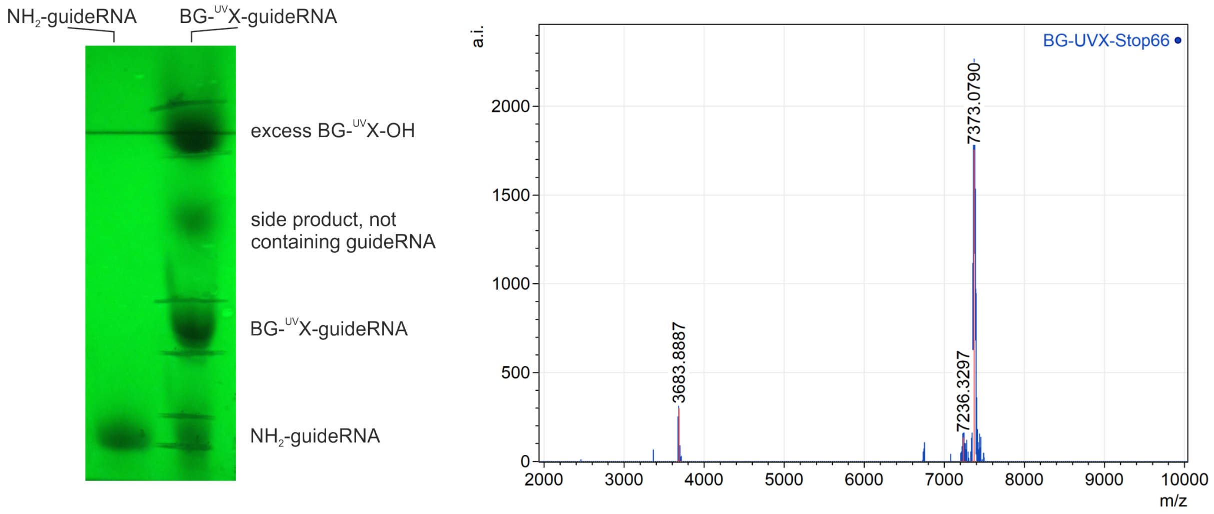

Sequences and modification patterns of guideRNAs used. 2'-OMe modified nucleotides are shown in italic, unmodified RNA bases are shown in bold. LNA bases are underlined. Phosphorothioate linkages have been marked with a star (*). All 5' modifications have been attached via a 6-carbon atom linker at the 5'-terminus.

terminal modification /guideRNA	sequence	Used in Figure
NH-Stop66	5´-**UCGGAACACCCCAGCACAGA**-3´	1d
BG-Stop66		1d
BG-^{UV}X-Stop66		1d
NH-Y701C	5´-A*G*UGUCUUGAU **ACA** UCCAGUU*C*C*U*T-3´	2d
BG-Y701C		2a,2d
$^{N7\text{-}Npom}$BG-Y701C		2b,
BG-^{UV}X-Y701C		2a, 2d
NH-T288A	5´-G*U*AGUTCGUAG **GCG** UAUUUCU*G*T*U*C-3´	2d
BG-T288A		2d
$^{N7\text{-}Npom}$BG-T288A		2d
BG-poly(U)	5´-UUUUUUUUUUUUUUUUUUUUUUUUUUUUUU -3´	3b, 3d
BC-poly(U)		3b, 3d
BG-BC-poly(U)		3d
$^{N7\text{-}Npom}$BG-BC-poly(U)		3e
$^{N7\text{-}Npom}$BG-BC-^{UV}X-poly(U)	5´-UTUUTUUTUUUTUUTUUUTUUTUUUTU -3´	3f

ADAR SDS-PAGE Shift Assay

Expression and purification of SNAP-ADAR3 was performed as previously described. SNAP-ADAR 3 (0.5 µM) and guideRNAs (2 µM) were diluted in 8 µl reaction buffer (10 mM Tris-HCl, 100 mM NaCl, 5% glycerol at pH 8.0). The reaction was carried out in PCR tubes which were wrapped in aluminum foil for light protection. After incubating for 30 min at 30 °C, the samples were irradiated on an UV table (UVP high performance UV transilluminator, 365 nm). 4 µl 4x SDS loading buffer (SDS (8% w/v), glycerol (40% v/v) and bromophenol blue (0,015% w/v) was added, and the samples were applied to SDS-PAGE gel electrophoresis (4% stacking gel, 12% loading gel, gel was run in the dark). GE Healthcare LMW protein marker was applied as size marker and the proteins were visualized by Coomassie staining. The staining solution was composed of Coomassie Brilliant Blue G-250 (0.02% w/v), $Al_2(SO_4)_3$ (5% w/v), EtOH (10% v/v) and phosphoric acid (2% v/v).

Cloning

The full sequence of all previously unpublished plasmids used in this study can be found in the appendix of the supplementary information and plasmid maps are found below.

eBFP-G3BP1 was created by amplifying the coding sequence of G3BP1 from the cDNA of Flp-In 293T-REx cells and fusing it with eBFP2 (obtained from Addgene, Cat.No.54595, https://www.addgene.org/54595/) by primer extension and restriction cloning. SNAP-$(eGFP)_3$ and NLS-SNAP-$(eGFP)_3$ were created by primer extension PCR of the mammalian cell optimized SNAPf-tag (New England Biolabs) and fusion to three copies of eGFP by restriction cloning. NLS-CLIP-$(mCherry)_3$ was created by primer extension PCR of the CLIPf tag (New England Biolabs) and fusion to three copies of the red fluorescent protein mCherry by restriction cloning.

For generating stable HeLa cell lines expressing two transgenes we used the PiggyBac transposase approach. The carrier plasmid PB-CA was obtained from Addgene (Cat.No.20960, https://www.addgene.org/20960/) and the CA resistance was exchanged with a puromycin resistance gene (PuroR). The CMV promoter was replaced by an expression cassette containing a bidirectional tetracycline responsive promoter. Subcloning of eBFP-G3BP1 and SNAP-$(eGFP)_3$ followed by a SV40 polyadenylation signal resulted in PiggyBac-Puro_SNAP-$(eGFP)_3$_eBFP-G3BP1 (used in fig.3b). Subcloning of NLS-CLIP-$(mCherry)_3$ and NLS-SNAP-$(eGFP)_3$ followed by a SV40 polyadenylation signal resulted in PiggyBac-Puro_NLS-SNAP-$(eGFP)_3$_NLS-CLIP-$(mCherry)_3$ (used in fig.3d and 3e). Subcloning of NLS-CLIP-$(mCherry)_3$ and SNAP-$(eGFP)_3$ followed by a SV40 polyadenylation signal resulted in PiggyBac-Puro_SNAP-$(eGFP)_3$_NLS-CLIP-$(mCherry)_3$ (used in fig.3f).

Note: The expression of the transgenes by the bidirectional promoter can be strongly enhanced in the presence of reverse tetracycline-controlled transactivator (rtTA) protein and doxycycline (Tet-On System). However, we found the basal expression of the minimal CMV promoters completely sufficient for the expression of (NLS)-SNAP-$(eGFP)_3$ and NLS-CLIP-$(mCherry)_3$. The expression of eBFP-G3BP1 is relatively low and showed more variation between cells. Since we observed that transient overexpression of eBFP-G3BP1 can trigger stress granules even in the absence of oxidative stress (data not shown) we also proceeded the experiments with the low basal expression of the minimal CMV.

For generating a stable, doxycycline-inducible HeLa cell line expressing the SNAP and CLIP tag on the plasma membrane surface, we used the previously described all-in-one, Tet-On 3G inducible PiggyBac plasmid XLone. XLone-GFP was obtained from Addgene (Cat.No. 96930, https://www.addgene.org/96930/) and the ORF of the blasticidin resistance gene (Bsd) was replaced with a puromycin resistance gene (PuroR) by restriction cloning. Afterwards GFP was entirely replaced by the SNAP-CLIP-display fusion protein which was a kind gift from Kai Johnssons laboratory (now Heidelberg), resulting in the XLone-Puro_SNAP-CLIP-display plasmid.

A mammalian codon-optimized PiggyBac-transposase (mPB) gene was subcloned into the commercial pcDNA3.1 backbone resulting in the pcDNA3.1_mPB plasmid.

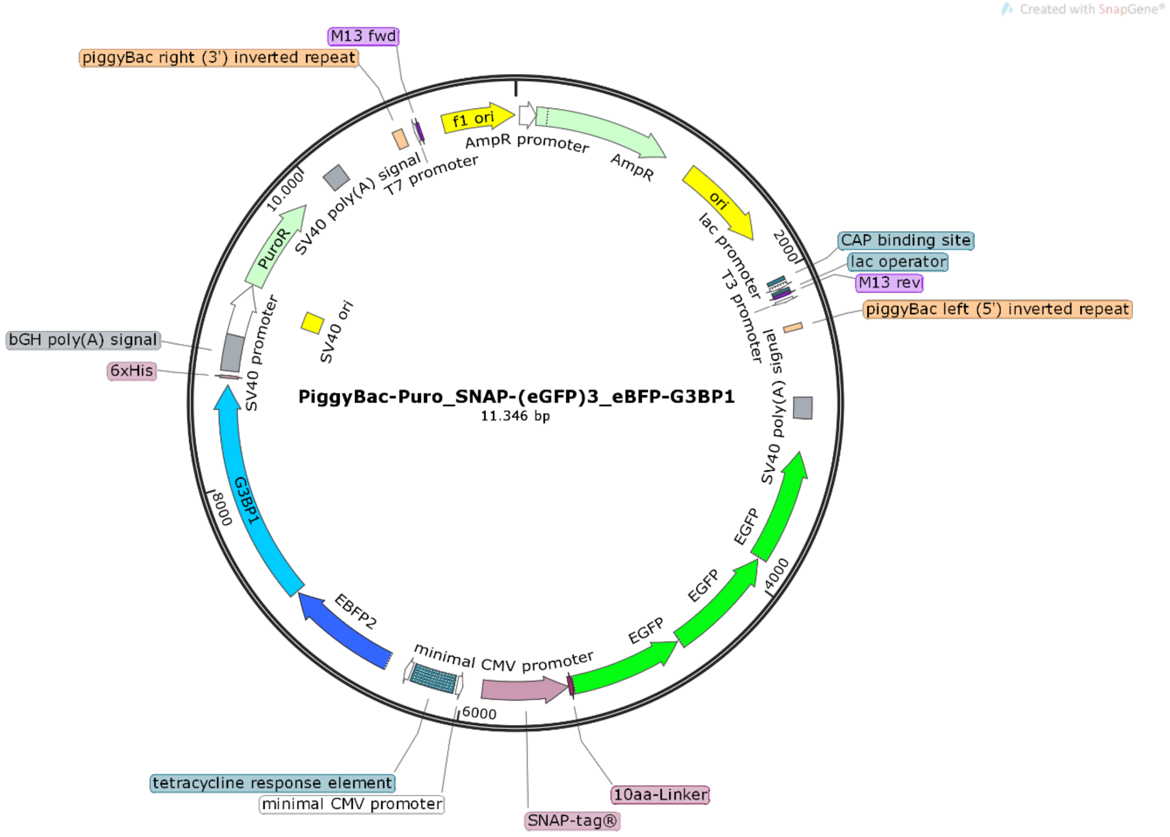
Created with SnapGene®
PiggyBac-Puro_SNAP-(eGFP)3_eBFP-G3BP1
11.346 bp
M13 fwd
piggyBac right (3') inverted repeat
f1 ori
AmpR promoter
AmpR
T7 promoter
SV40 poly(A) signal
10.000
ori
2000
lac promoter
T3 promoter
CAP binding site
lac operator
M13 rev
piggyBac left (5') inverted repeat
PuroR
SV40 ori
SV40 promoter
bGH poly(A) signal
6xHis
SV40 poly(A) signal
G3BP1
8000
EGFP
4000
EGFP
EBFP2
EGFP
minimal CMV promoter
6000
tetracycline response element
minimal CMV promoter
SNAP-tag®
10aa-Linker

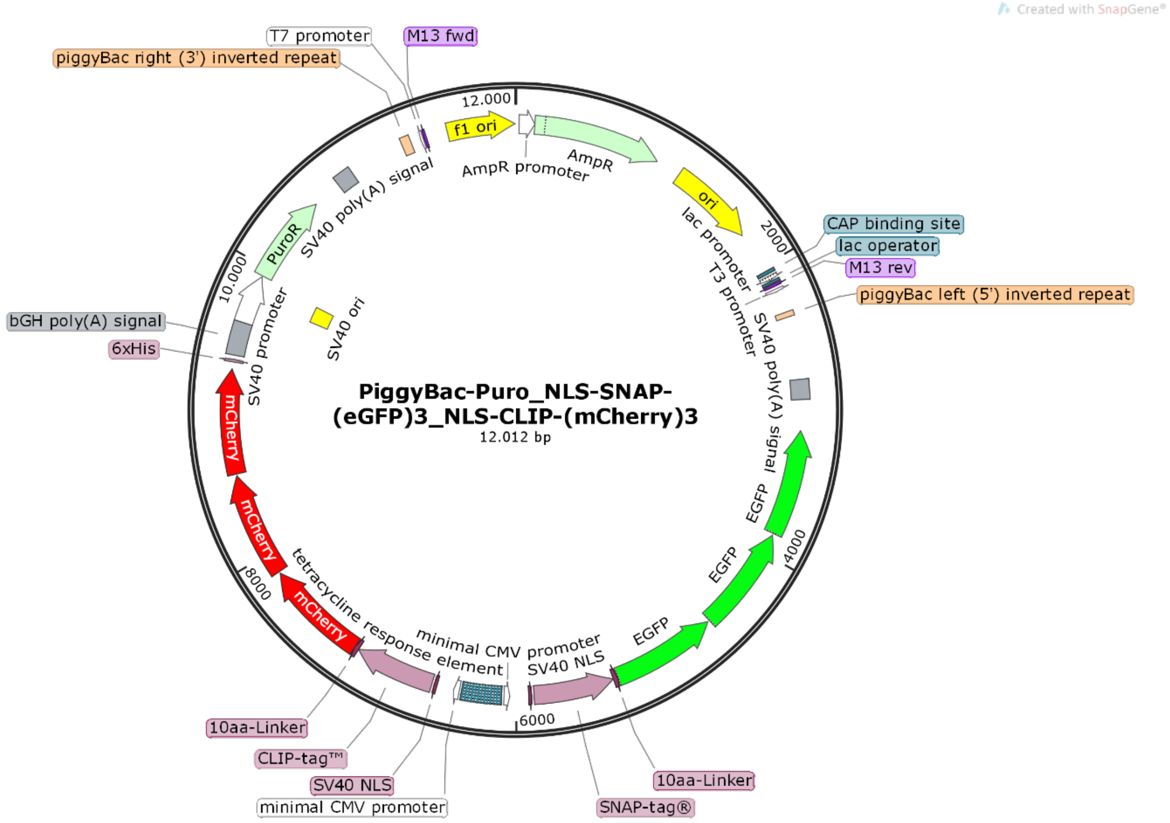
Created with SnapGene®
PiggyBac-Puro_NLS-SNAP-(eGFP)3_NLS-CLIP-(mCherry)3
12.012 bp
T7 promoter
M13 fwd
piggyBac right (3') inverted repeat
12.000
f1 ori
AmpR
AmpR promoter
SV40 poly(A) signal
ori
2000
lac promoter
T3 promoter
CAP binding site
lac operator
M13 rev
piggyBac left (5') inverted repeat
PuroR
10.000
SV40 ori
SV40 promoter
bGH poly(A) signal
6xHis
SV40 poly(A) signal
mCherry
mCherry
mCherry
8000
EGFP
4000
EGFP
EGFP
tetracycline response element
minimal CMV promoter
SV40 NLS
6000
10aa-Linker
CLIP-tag™
SV40 NLS
minimal CMV promoter
SNAP-tag®
10aa-Linker

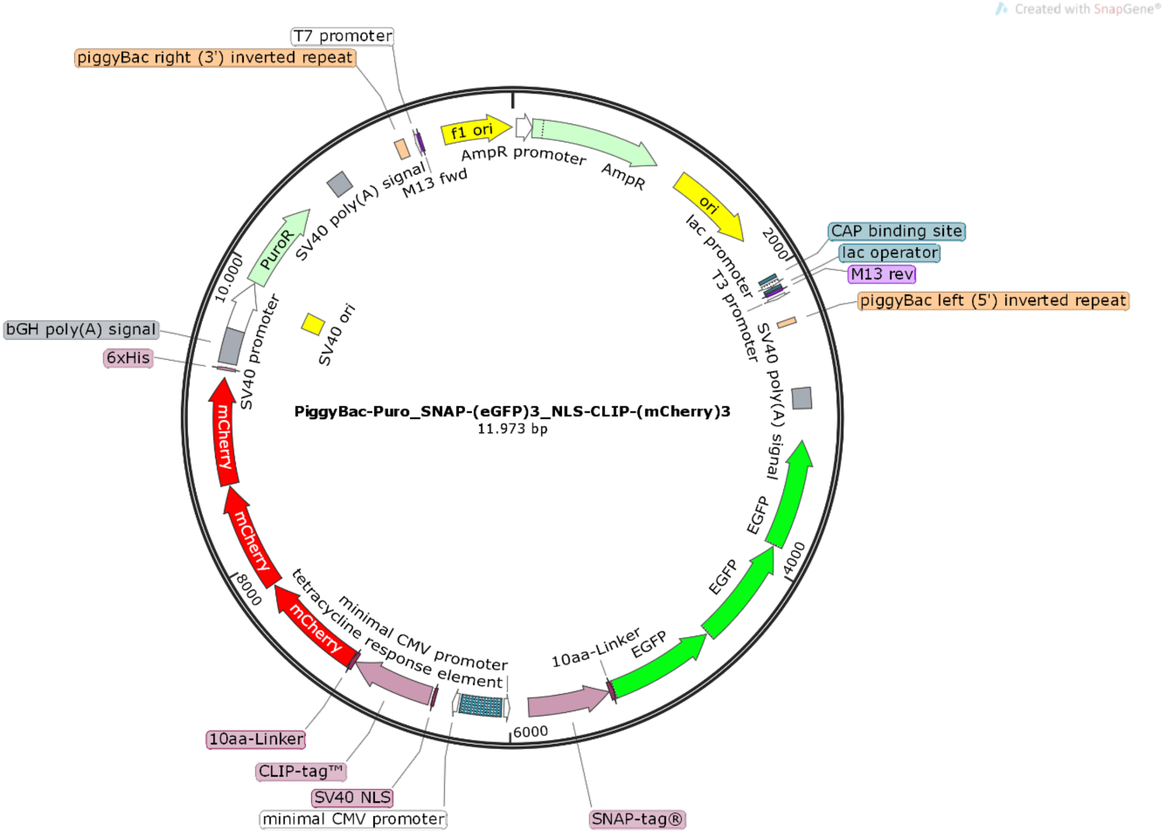
Created with SnapGene®
PiggyBac-Puro_SNAP-(eGFP)3_NLS-CLIP-(mCherry)3
11.973 bp
T7 promoter
piggyBac right (3') inverted repeat
f1 ori
AmpR promoter
AmpR
ori
M13 fwd
SV40 poly(A) signal
PuroR
CAP binding site
lac operator
M13 rev
piggyBac left (5') inverted repeat
lac promoter
T3 promoter
SV40 poly(A) signal
SV40 ori
SV40 promoter
bGH poly(A) signal
6xHis
mCherry
mCherry
mCherry
EGFP
EGFP
EGFP
10aa-Linker
tetracycline response element
minimal CMV promoter
10aa-Linker
CLIP-tag™
SV40 NLS
minimal CMV promoter
SNAP-tag®
2000
4000
6000
8000
10.000

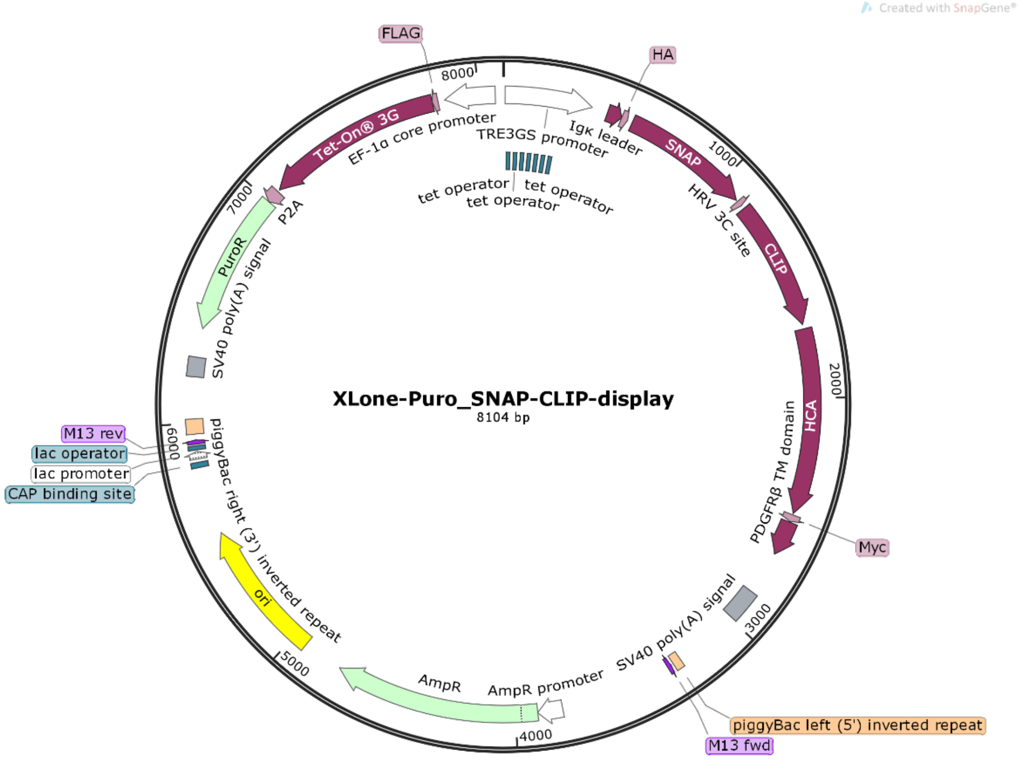
Created with SnapGene®
XLone-Puro_SNAP-CLIP-display
8104 bp
FLAG
HA
Tet-On® 3G
EF-1α core promoter
TRE3GS promoter
Igκ leader
SNAP
HRV 3C site
CLIP
tet operator
tet operator
tet operator
P2A
PuroR
SV40 poly(A) signal
HCA
PDGFRβ TM domain
Myc
M13 rev
lac operator
lac promoter
CAP binding site
piggyBac right (3') inverted repeat
ori
AmpR
AmpR promoter
SV40 poly(A) signal
piggyBac left (5') inverted repeat
M13 fwd
1000
2000
3000
4000
5000
6000
7000
8000

Cell culture

If not stated otherwise, cells were cultivated in Dulbecco's Modified Eagle Medium (DMEM, Thermo Fisher Scientific) supplemented with 10 % fetal bovine serum (FBS, Thermo Fisher Scientific) at 37 °C with 5 % CO_2 in a water saturated steam atmosphere. For sub cultivation and seeding, cells were washed with phosphate-buffered saline (PBS), detached with 0.25 % trypsin/EDTA (Sigma Aldrich), and resuspended in fresh DMEM/FBS. Sub cultivation was performed every 3-5 days. For editing experiments Flp-In 293T-REx cells, stably expressing SNAP-ADAR1Q were used. The creation and sub cultivation of this cell line has been previously described.

Creation of HeLa PiggyBac cells

We used the previously described mPB PiggyBac transposase for generating stable transgenic HeLa cell lines. For this, 1×10^5 HeLa cells (DSMZ, Braunschweig, Germany, no: ACC 57) were seeded in 0.5 ml DMEM/FBS in a 24-well plate. After 24 h, medium was replaced with 0.4 ml fresh DMEM/FBS, and 800 ng of the respective PiggyBac carrier plasmid and 200 ng pcDNA3.1_mPB were transfected with 3 µl Fugene6 (Promega) in 100 µl OptiMEM according to the manual. After 24 h, the cells were transferred to two wells of a 6 well plate. 48 h later, the medium was replaced with DMEM/10% FBS/5 µg/ml puromycin. After 12 days of selection, the medium was replaced by DMEM/FBS. Cells were used without monoclonal selection and subsequently cultured in DMEM/FBS.

RNA editing experiments

Induction, transfection, and irradiation

Transgene expression in Flp-In T-REx SNAP-ADAR1Q cells was induced by seeding 1.5 x 10^6 cells in a 6 well in 2.5 ml DMEM/FBS +15 µg/ml blasticidin +100 µg/ml hygromycin (DMEM/FBS/B/H) containing 10 ng/ml doxycycline. After 24 hours cells were washed with PBS, trypsinated, and taken up in fresh DMEM/FBS. Cells were pelleted (300 g, 5 min) and resuspended to a concentration of 800,000 cells/ml in DMEM/FBS+HEPES without phenol red (Thermo Fisher Scientific) containing 15 ng/ml doxycycline.

Per 96-well 0.5 µl Lipofectamine 2000 (Thermo Fisher Scientific) was diluted in 25 µl OptiMEM (Thermo Fisher Scientific). After 5 min of incubation the solution was added to a predilution of guideRNA (0.5 pmol for Y701C guideRNAs and 2 pmol for T288A guideRNAs) in 25 µl OptiMEM and the mix was incubated another 15 min. 50 µl guideRNA/Lipofectamine mix was transferred to a well of a 96-well plate and 100 µl cell suspension were added for a final amount of 80,000 cells per well. For every sample, two wells were used and processed in parallel. The cells were kept in the incubator and protected from light until harvest.

For UV irradiation, the plate was sealed with parafilm and placed on a High-Performance UV Transilluminator (UVP, setting = high, 7.9±0.2 mW/cm^2 light with 365 nm wavelength) for 2 or 3 min at the indicated time point.

Note: If guideRNA was irradiated before transfection, the guideRNA dilution in OptiMEM was transferred to a 96-well plate and irradiated as described above and afterwards transferred back to a reaction tube for incubation with transfection reagent.

Cell harvest, RNA isolation, RT-PCR and sequencing

Cells were harvested at the indicated time point by discarding the medium and adding 50 µl RLT lysis buffer (Qiagen) per well. Lysate from two wells with the same treatment was pooled and RNA isolation was performed with Monarch RNA Cleanup Kit (New England Biolabs) according to the manual. Total RNA was eluted with 30 µl RNase free water and concentration was determined by measuring the absorbance at 260 nm.

STAT1 Y701C editing site was amplified using the following primer set:
fw = GCTTCATCAGCAAGGAGCGAGAGCG, rev = CTTCAGACACAGAAATCAACTCATTC.

STAT1 T228A editing site was amplified using the following primer set:
fw = GAATGTCACTGAACTTACCCAGAATGC, rev = CACCAACAGTCTCAACTTCACAGTG.

If both sites were detected from one sample two separate RT-PCR reactions were setup. Reverse transcription and PCR were performed using the One-Step RT PCR Kit (biotech-rabbit GmbH). Typically 500 ng RNA were diluted with nuclease free water to 9.25 µl. 1 µl reverse primer (10 µM) was added and the reaction mix was incubated for 1 min at 90 °C. 1 µl forward primer (10 µM), 12.5 µl One Step Mix (2x) and 1.25 µl RT-RI Blend (20x) were added. RT-PCR was carried out in a PCR cycler starting with reverse transcription at 50°C for 30 min and an initial denaturation step at 95°C. 35 cycles of denaturation (95°C, 15 s), annealing (51°C, 30 s) and elongation (72°C, 40 s) were performed, followed by a final extension step (68°C, 10 min).

The resulting PCR fragments (~400 bp) were loaded on a 1.4% TAE-agarose gel and purified using the NucleoSpin Gel and PCR Cleanup Set (Macherey Nagel). 120 ng DNA were sent to sequencing (Eurofins Genomics, primer STAT1 Y701C: GGCTGCTGAGAA-TATTCCTGAGAATC, primer STAT1 T288A: GAATGTCACTGAACTTACCCAGAATGC). A-to-I editing yields were determined by dividing the peak height for guanosine by the sum of the peak heights for both adenosine and guanosine.

Stress granule imaging experiments

50,000 Hela PiggyBac cells were reverse transfected with 25 pmol guideRNA and 0.25 µl Lipofectamine 2000 (Thermo Fisher Scientific) in 50 µl OptiMem (Thermo Fisher Scientific) into a 96-well cell culture-treated plate. Cell concentration prior transfection was adjusted to 500,000 cells per ml in DMEM/FBS. After 4 hours, cells were washed with PBS, trypsinated and re-suspended in 300 µl DMEM/FBS. 3x100 µl were transferred into three wells of a 96-well imaging plate with a 170 µm glass bottom (Eppendorf). The cells were kept in the incubator and protected from light for 24 hours. Stress granule formation was induced by changing the medium to DMEM/FBS containing 50 µM As_2O_3 for 30 min. For imaging the medium was changed to 100 µl DMEM/FBS+HEPES without phenol red (Thermo Fisher Scientific). Irradiation was performed on a Nikon Elipse Ti2-E microscope equipped with a lumencor® AuraII light engine and a 60X oil objective (numerical aperture 1.4). 390 nm light (source intensity 100%) and a quad band excitation filter (Chroma 89402x) were used. Irradiation time was typically 100 or 300 ms.

Note: Performing reverse transfection directly in glass bottom plates resulted in lower viability of the cells (data not shown). Furthermore, a low density of cells is required for imaging (~10-15.000 cells per 96-well). However, reverse transfection at such low cell density resulted in low viablity. Performing transfection in a regular cell culture-treated plastic well plate and at high concentration (50.000 cells per 96-well) increased the viability of the cells. The additional trypsination step that is required to transfer them to the imaging plate later, also reduces the number of liposomes seen in microscopy.

Surface labeling experiments

10,000 HeLa XLone-Puro SNAP-CLIP-display cells were seeded into a 96-well imaging plate with a 170 µm glass bottom (Eppendorf) in 100 µl DMEM/FBS containing 500 ng/µl doxycycline to induce transgene expression. After 16 h, 50 µl of DMEM/FBS were added, containing 1.5% NucBlue (Thermo Fisher Scientific),15 µM BC-Atto594, and 15 µM BG-Atto488 or BG-^{UV}X-Atto488. The cells were kept in the incubator and protected from light for 15-30 min and washed twice with 100 µl DMEM/FBS+HEPES without phenol red (Thermo Fisher Scientific). Irradiation was performed as described above for stress granule imaging experiments.

Microscopy

Microscopy was performed on a Nikon Elipse Ti2-E inverted fluorescent microscope equipped with a photometrics® Prime 95B camera and a lumencor® AuraII light engine. All pictures were recorded using a 60X oil objective (numerical aperture 1.4) and Olympus IMMOIL-F30CC immersion oil. The excitation wavelengths and corresponding filter sets used to record each channel are specified in the following table. Typically, a z-stack covering 6 µm (0.2 µm steps) was recorded. The images were deconvoluted using Nikon NIS-Elements software and a single layer (z resolution ~0.6 µM) is displayed. Assignment of lookup tables, contrast settings and cropping were performed in FIJI ImageJ. The same acquisition settings were chosen for every channel within one subfigure.

Fluorophore	Excitation	Filter
eGFP	475 nM	Excitation: Chroma 89402x<br>Beamsplitter: Chroma 89402bs<br>Emission: Chroma 89402m
mCherry	575 nM	F66-016NXL CFP/YFP/mCherry/Cy7 Quad filter (AHF, Germany)
Atto488	475 nM	Excitation: Chroma ET500/20x<br>Beamsplitter: Chroma T515lp<br>Emission: Chroma ET535/30m
Atto594	575 nM	Excitation: Semrock 585/29 BrightLine HC<br>Beamsplitter: Chroma T610LPXR<br>Emission: Semrock 650/60 BrightLine HC
eBFP, NucBlue, Irradiation	390 nM	Excitation: Chroma 89402x<br>Beamsplitter: Chroma 89402bs<br>Emission: Chroma 89402m

Quantification of fluorescent signal in stress granules

Quantification of the red and green signal in stress granules was performed in Nikon NIS-Elements software using a maximum projection in z. The experiments were performed with the NLS-SNAP-(eGFP)$_3$+NLS-CLIP-(mCherry)$_3$ cell line, due to the low background signal in the cytoplasm. Since the NLS-CLIP-(mCherry)$_3$ signal served as reference, we manually chose cells with clearly visible stress granules in the red channel for quantification. The cytoplasm was defined as region of interest (ROI) by manually outlining the cells. The nucleus was detected in the red channel and excluded using the bright spot detection function of Nikon NIS-Elements software (minimal ⌀ set to 15 µM, example seen below in **a**). Background was subtracted using rolling ball correction (ball size = 1.5 µM, example seen below in **b**) and stress granules were detected using bright spot detection in NIS-Elements software (settings: growing 400-600, example seen below in **c**). The mean intensity of eGFP and mCherry signal for each spot was measured and the ratio calculated. For each cell the mean of the ratios of all quantified granules is shown (figure 3d and figure 3e). The values for all individual granules quantified are shown below in figure S5b. Significance was calculated in GraphPad Prism 8 (version 8.4.3) using an unpaired Welch's t test.

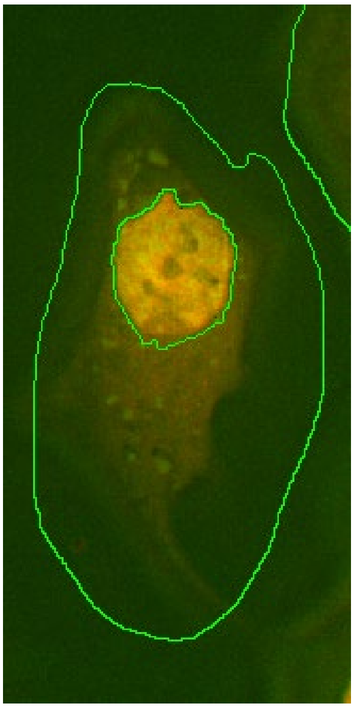

a)

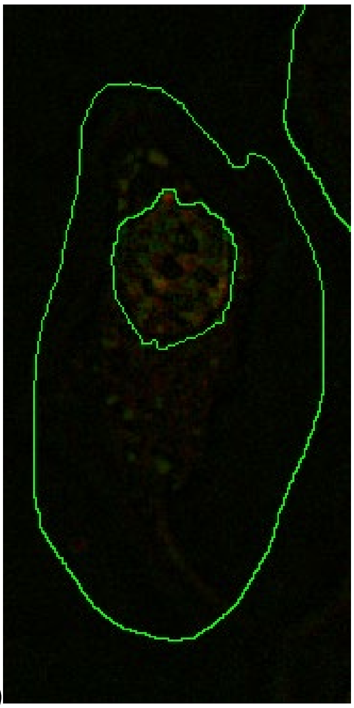

b)

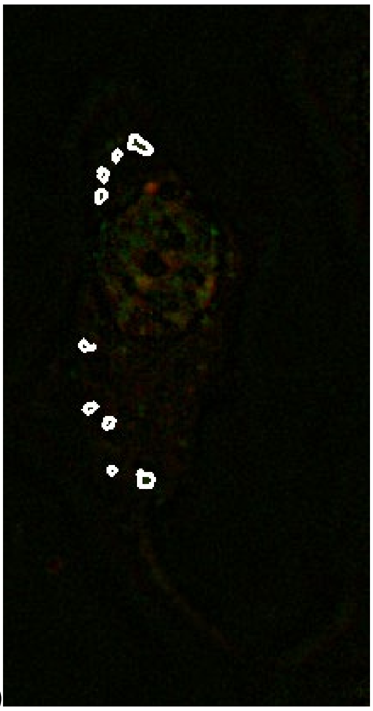

c)

The same quantification could not be applied to the correlation of SNAP-(eGFP)$_3$ and G3BP1 since the expression levels of eBFP-G3BP1 varied stronger and the transfection efficiency influenced the amount of SNAP-(eGFP)$_3$ in the granules relative to eBFP-G3BP1. Instead, the local correlation of eBFP-G3BP1 and SNAP-(eGFP)$_3$ was determined (figure 3b).

Since the eBFP-G3BP1 signal served as reference, we manually chose cells with clearly visible stress granules in the blue channel for quantification. The cells were manually outlined and defined as region of interest (ROI). An example is shown below in **d**. Stress granules were selected using bright spot detection in NIS-Elements software (1-1.2 µM size) and a circular selection (⌀ 15px) around each granule was analyzed (example shown below in **e**). Pearson correlation was determined for the eGFP and G3BP1 channel within each of the selections (figure S5a) and averaged for all stress granules of one cell (figure 3b). Significance was calculated in GraphPad Prism 8 (version 8.4.3) using an unpaired Welch's t test.
Note: If the Pearson correlation is determined for the whole cell it will mainly reflect whether both signals are cytoplasmatic or if one of them is nuclear. Determining the Pearson correlation within a defined radius surrounding each granule circumvents this problem. However, it is important to ensure that the area analyzed is bigger than the average granule size).

d) 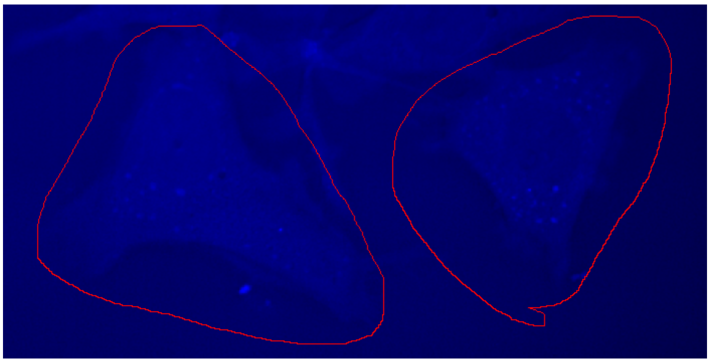

e) 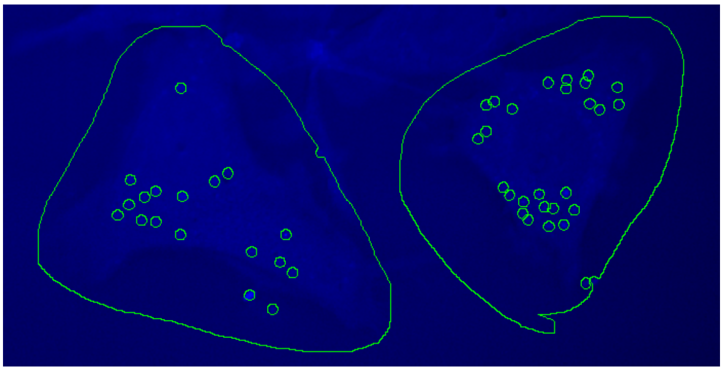

Appendix

Unedited image of SDS-PAGE, Fig. 1d

Marker NH BG BG-^{UV}X-guideRNA

- 5 s 15 s 30 s 60 s UV

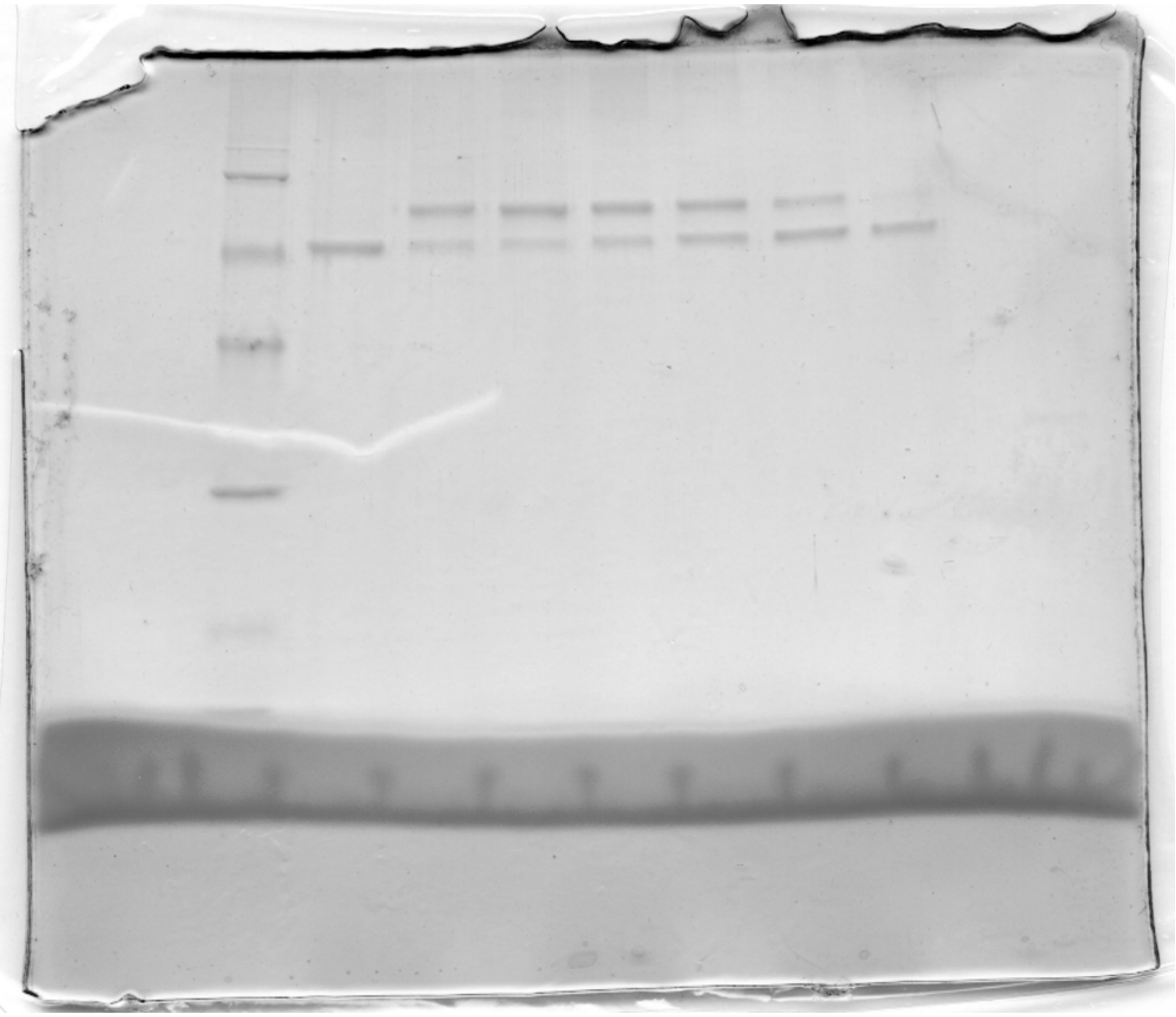

Full sequences of plasmids used in this study

PiggyBac-Puro_SNAPf-3xeGFP+BFP-G3BP

CAGGTGGCACTTTTCGGGGAAATGTGCGCGGAACCCCTATTTGTTTATTTTTCTAAATACATTCAAAT
ATGTATCCGCTCATGAGACAATAACCCTGATAAATGCTTCAATAATATTGAAAAAGGAAGAGTATGAG
TATTCAACATTTCCGTGTCGCCCTTATTCCCTTTTTTGCGGCATTTTGCCTTCCTGTTTTTGCTCACC
CAGAAACGCTGGTGAAAGTAAAAGATGCTGAAGATCAGTTGGGTGCACGAGTGGGTTACATCGAACTG
GATCTCAACAGCGGTAAGATCCTTGAGAGTTTTCGCCCCGAAGAACGTTTTCCAATGATGAGCACTTT
TAAAGTTCTGCTATGTGGCGCGGTATTATCCCGTATTGACGCCGGGCAAGAGCAACTCGGTCGCCGCA
TACACTATTCTCAGAATGACTTGGTTGAGTACTCACCAGTCACAGAAAAGCATCTTACGGATGGCATG
ACAGTAAGAGAATTATGCAGTGCTGCCATAACCATGAGTGATAACACTGCGGCCAACTTACTTCTGAC
AACGATCGGAGGACCGAAGGAGCTAACCGCTTTTTTGCACAACATGGGGGATCATGTAACTCGCCTTG
ATCGTTGGGAACCGGAGCTGAATGAAGCCATACCAAACGACGAGCGTGACACCACGATGCCTGTAGCA
ATGGCAACAACGTTGCGCAAACTATTAACTGGCGAACTACTTACTCTAGCTTCCCGGCAACAATTAAT
AGACTGGATGGAGGCGGATAAAGTTGCAGGACCACTTCTGCGCTCGGCCCTTCCGGCTGGCTGGTTTA
TTGCTGATAAATCTGGAGCCGGTGAGCGTGGGTCTCGCGGTATCATTGCAGCACTGGGGCCAGATGGT
AAGCCCTCCCGTATCGTAGTTATCTACACGACGGGGAGTCAGGCAACTATGGATGAACGAAATAGACA
GATCGCTGAGATAGGTGCCTCACTGATTAAGCATTGGTAACTGTCAGACCAAGTTTACTCATATATAC
TTTAGATTGATTTAAAACTTCATTTTTAATTTAAAAGGATCTAGGTGAAGATCCTTTTTGATAATCTC
ATGACCAAAATCCCTTAACGTGAGTTTTCGTTCCACTGAGCGTCAGACCCCGTAGAAAAGATCAAAGG
ATCTTCTTGAGATCCTTTTTTTCTGCGCGTAATCTGCTGCTTGCAAACAAAAAAACCACCGCTACCAG
CGGTGGTTTGTTTGCCGGATCAAGAGCTACCAACTCTTTTTCCGAAGGTAACTGGCTTCAGCAGAGCG
CAGATACCAAATACTGTCCTTCTAGTGTAGCCGTAGTTAGGCCACCACTTCAAGAACTCTGTAGCACC
GCCTACATACCTCGCTCTGCTAATCCTGTTACCAGTGGCTGCTGCCAGTGGCGATAAGTCGTGTCTTA
CCGGGTTGGACTCAAGACGATAGTTACCGGATAAGGCGCAGCGGTCGGGCTGAACGGGGGGTTCGTGC
ACACAGCCCAGCTTGGAGCGAACGACCTACACCGAACTGAGATACCTACAGCGTGAGCTATGAGAAAG
CGCCACGCTTCCCGAAGGGAGAAAGGCGGACAGGTATCCGGTAAGCGGCAGGGTCGGAACAGGAGAGC
GCACGAGGGAGCTTCCAGGGGGAAACGCCTGGTATCTTTATAGTCCTGTCGGGTTTCGCCACCTCTGA
CTTGAGCGTCGATTTTTGTGATGCTCGTCAGGGGGGCGGAGCCTATGGAAAAACGCCAGCAACGCGGC
CTTTTTACGGTTCCTGGCCTTTTGCTGGCCTTTTGCTCACATGTTCTTTCCTGCGTTATCCCCTGATT
CTGTGGATAACCGTATTACCGCCTTTGAGTGAGCTGATACCGCTCGCCGCAGCCGAACGACCGAGCGC
AGCGAGTCAGTGAGCGAGGAAGCGGAAGAGCGCCCAATACGCAAACCGCCTCTCCCCGCGCGTTGGCC
GATTCATTAATGCAGCTGGCACGACAGGTTTCCCGACTGGAAAGCGGGCAGTGAGCGCAACGCAATTA
ATGTGAGTTAGCTCACTCATTAGGCACCCCAGGCTTTACACTTTATGCTTCCGGCTCGTATGTTGTGT
GGAATTGTGAGCGGATAACAATTTCACACAGGAAACAGCTATGACCATGATTACGCCAAGCTCGGAAT
TAACCCTCACTAAAGGGAACAAAAGCTGGGTACCTCGCGCGACTTGGTTTGCCATTCTTTAGCGCGCG
TCGCGTCACACAGCTTGGCCACAATGTGGTTTTTGTCAAACGAAGATTCTATGACGTGTTTAAAGTTT
AGGTCGAGTAAAGCGCAAATCTTTTTTAACCCTAGAAAGATAGTCTGCGTAAAATTGACGCATGCATT
CTTGAAATATTGCTCTCTCTTTCTAAATAGCGCGAATCCGTCGCTGTGCATTTAGGACATCTCAGTCG
CCGCTTGGAGCTCCCGTGAGGCGTGCTTGTCAATGCGGTAAGTGTCACTGATTTTGAACTATAACGAC
CGCGTGAGTCAAAATGACGCATGATTATCTTTTACGTGACTTTTAAGATTTAACTCATACGATAATTA
TATTGTTATTTCATGTTCTACTTACGTGATAACTTATTATATATATATTTTCTTGTTATAGATATCGT
GACTAATATATAATAAAATGGGTAGTTCTTTAGACGATGAGCATATCCTCTCTGCTCTTCTGCAAAGC
GATGACGAGCTTGTTGGCTAGCGCGCTGCTTCGCGATGTACGGGCCAGATATACGCGTTGACATTGAT
TATTGACTAGTAACTTGTTTATTGCAGCTTATAATGGTTACAAATAAGGCAATAGCATCACAAATTTC
ACAAATAAGGCATTTTTTTCACTGCATTCTAGTTTTGGTTTGTCCAAACTCATCAATGTATCTTATCA
TGTCTGGATCTCAAATCCCTCGGAAGCTGCGCCTGTCTTAGGTTGGAGTGATACATTTTTATCACTTT
TACCCGTCTTTGGATTAGGCAGTAGCTCTGACGGCCCTCCTGTCTTAGGTTAGTGAAAAATGTCACTC
TCTTACCCGTCATTGGCTGTCCAGCTTAGCTCGCAGGGGAGGTGGTCTGGGCCCATCGATTCTAGAAT
TAACCGGTGACCGGCTTGTACAGCTCGTCCATGCCGAGAGTGATCCCGGCGGCGGTCACGAACTCCAG
CAGGACCATGTGATCGCGCTTCTCGTTGGGGTCTTTGCTCAGGGCGGACTGGGTGCTCAGGTAGTGGT

TGTCGGGCAGCAGCACGGGGCCGTCGCCGATGGGGGTGTTCTGCTGGTAGTGGTCGGCGAGCTGCACG
CTGCCGTCCTCGATGTTGTGGCGGATCTTGAAGTTCACCTTGATGCCGTTCTTCTGCTTGTCGGCCAT
GATATAGACGTTGTGGCTGTTGTAGTTGTACTCCAGCTTGTGCCCCAGGATGTTGCCGTCCTCCTTGA
AGTCGATGCCCTTCAGCTCGATGCGGTTCACCAGGGTGTCGCCCTCGAACTTCACCTCGGCGCGGGTC
TTGTAGTTGCCGTCGTCCTTGAAGAAGATGGTGCGCTCCTGGACGTAGCCTTCGGGCATGGCGGACTT
GAAGAAGTCGTGCTGCTTCATGTGGTCGGGGTAGCGGCTGAAGCACTGCACGCCGTAGGTCAGGGTGG
TCACGAGGGTGGGCCAGGGCACGGGCAGCTTGCCGGTGGTGCAGATGAACTTCAGGGTCAGCTTGCCG
TAGGTGGCATCGCCCTCGCCCTCGCCGGACACGCTGAACTTGTGGCCGTTTACGTCGCCGTCCAGCTC
GACCAGGATGGGCACCACCCCGGTGAACAGCTCCTCGCCCTTGCTCACGCCGGTGACCGGCTTGTACA
GCTCGTCCATGCCGAGAGTGATCCCGGCGGCGGTCACGAACTCCAGCAGGACCATGTGATCGCGCTTC
TCGTTGGGGTCTTTGCTCAGGGCGGACTGGGTGCTCAGGTAGTGGTTGTCGGGCAGCAGCACGGGGCC
GTCGCCGATGGGGGTGTTCTGCTGGTAGTGGTCGGCGAGCTGCACGCTGCCGTCCTCGATGTTGTGGC
GGATCTTGAAGTTCACCTTGATGCCGTTCTTCTGCTTGTCGGCCATGATATAGACGTTGTGGCTGTTG
TAGTTGTACTCCAGCTTGTGCCCCAGGATGTTGCCGTCCTCCTTGAAGTCGATGCCCTTCAGCTCGAT
GCGGTTCACCAGGGTGTCGCCCTCGAACTTCACCTCGGCGCGGGTCTTGTAGTTGCCGTCGTCCTTGA
AGAAGATGGTGCGCTCCTGGACGTAGCCTTCGGGCATGGCGGACTTGAAGAAGTCGTGCTGCTTCATG
TGGTCGGGGTAGCGGCTGAAGCACTGCACGCCGTAGGTCAGGGTGGTCACGAGGGTGGGCCAGGGCAC
GGGCAGCTTGCCGGTGGTGCAGATGAACTTCAGGGTCAGCTTGCCGTAGGTGGCATCGCCCTCGCCCT
CGCCGGACACGCTGAACTTGTGGCCGTTTACGTCGCCGTCCAGCTCGACCAGGATGGGCACCACCCCG
GTGAACAGCTCCTCGCCCTTGCTCACGCCGGTGACCGGCTTGTACAGCTCGTCCATGCCGAGAGTGAT
CCCGGCGGCGGTCACGAACTCCAGCAGGACCATGTGATCGCGCTTCTCGTTGGGGTCTTTGCTCAGGG
CGGACTGGGTGCTCAGGTAGTGGTTGTCGGGCAGCAGCACGGGGCCGTCGCCGATGGGGGTGTTCTGC
TGGTAGTGGTCGGCGAGCTGCACGCTGCCGTCCTCGATGTTGTGGCGGATCTTGAAGTTCACCTTGAT
GCCGTTCTTCTGCTTGTCGGCCATGATATAGACGTTGTGGCTGTTGTAGTTGTACTCCAGCTTGTGCC
CCAGGATGTTGCCGTCCTCCTTGAAGTCGATGCCCTTCAGCTCGATGCGGTTCACCAGGGTGTCGCCC
TCGAACTTCACCTCGGCGCGGGTCTTGTAGTTGCCGTCGTCCTTGAAGAAGATGGTGCGCTCCTGGAC
GTAGCCTTCGGGCATGGCGGACTTGAAGAAGTCGTGCTGCTTCATGTGGTCGGGGTAGCGGCTGAAGC
ACTGCACGCCGTAGGTCAGGGTGGTCACGAGGGTGGGCCAGGGCACGGGCAGCTTGCCGGTGGTGCAG
ATGAACTTCAGGGTCAGCTTGCCGTAGGTGGCATCGCCCTCGCCCTCGCCGGACACGCTGAACTTGTG
GCCGTTTACGTCGCCGTCCAGCTCGACCAGGATGGGCACCACCCCGGTGAACAGCTCCTCGCCCTTGC
TCACGCCGGTCCCTGGCGCGCCTCCGCCTGCAGGACCCAGCCCAGGCTTGCCCAGTCTGTGGCCCTCG
TGGGCCAGCAGCCACTCTTTCACGGCGAGCCCGCCCTCGTAGCCCCCCACGTCCAGGTCGCCCTGCAC
CACCCGGTGGCAGGGGATCAGAATGGGCACGGGATTTCCGCTCAGGGCGGTTTTCACGGCGGCGGTGG
CGGCGGGATTGCCGGCCAGGGCGGCCAGGTGGCTGTAGCTGATGACCTCTCCGAACTTCACCACTTTC
AGCAGTTTCCACAGCACCTGGCGGGTAAAGCTCTCCTGCTGGAACACTGGGTGGTGCAGGGCTGGCAC
AGGGAACTCCTCGATGGCCTCAGGCTGGTGAAAGTAGGCGTTGAGCCAGGCGGTGGCCTGCATCAGTG
GCTCTGGTCCGCCCAGCACGGCGGCTGGGGCAGGCACTTCCACGGCGTCGGCGGCAGATGTTCCTTTG
CCCAGGAAGATGATACGGTGCAGGCCCTGTTCGCACCCAGACAGTTCCAGCTTGCCCAGAGGGCTATC
CAGGGTGGTGCGCTTCATTTCGCAGTCTTTGTCGCCGGCCATGCGGCCGCCGCGTCCTAGGTTTCGAC
CGCGGAGGCTGGATCGGTCCCGGTGTCTTCTATGGAGGTCAAAACAGCGTGGATGGCGTCTCCAGGCG
ATCTGACGGTTCACTAAACGAGCTCTGCTTATATAGGCCTCCCACCGTACACGCCTACAAGCTTCTTT
CACTTTTCTCTATCACTGATAGGGAGTGGTAAACTCGACTTTCACTTTTCTCTATCACTGATAGGGAG
TGGTAAACTCGACTTTCACTTTTCTCTATCACTGATAGGGAGTGGTAAACTCGACTTTCACTTTTCTC
TATCACTGATAGGGAGTGGTAAACTCGACTTTCACTTTTCTCTATCACTGATAGGGAGTGGTAAACTC
GACTTTCACTTTTCTCTATCACTGATAGGGAGTGGTAAACTCGACTTTCACTTTTCTCTATCACTGAT
AGGGAGTGGTAAACTCGAGGGTAGGCGTGTACGGTGGGAGGCCTATATAAGCAGAGCTCGTTTAGTGA
ACCGTCAGATCGCCTGGAGACGCCATCCACGCTGTTTTGACCTCCATAGAAGACACCGGGACCGATCC
AGCCTCCGCGGAATTCTTAATAGCGGCCGCATGGCCGGCGTGAGCAAGGGCGAGGAGCTGTTCACCGG
GGTGGTGCCCATCCTGGTCGAGCTGGACGGCGACGTAAACGGCCACAAGTTCAGCGTGAGGGGCGAGG
GCGAGGGCGATGCCACCAACGGCAAGCTGACCCTGAAGTTCATCTGCACCACCGGCAAGCTGCCCGTG
CCCTGGCCCACCCTCGTGACCACCCTGAGCCACGGCGTGCAGTGCTTCGCCCGCTACCCCGACCACAT
GAAGCAGCACGACTTCTTCAAGTCCGCCATGCCCGAAGGCTACGTCCAGGAGCGCACCATCTTCTTCA
AGGACGACGGCACCTACAAGACCCGCGCCGAGGTGAAGTTCGAGGGCGACACCCTGGTGAACCGCATC
GAGCTGAAGGGCGTCGACTTCAAGGAGGACGGCAACATCCTGGGGCACAAGCTGGAGTACAACTTCAA

CAGCCACAACATCTATATCATGGCCGTCAAGCAGAAGAACGGCATCAAGGTGAACTTCAAGATCCGCC
ACAACGTGGAGGACGGCAGCGTGCAGCTCGCCGACCACTACCAGCAGAACACCCCCATCGGCGACGGC
CCCGTGCTGCTGCCCGACAGCCACTACCTGAGCACCCAGTCCGTGCTGAGCAAAGACCCCAACGAGAA
GCGCGATCACATGGTCCTGCTGGAGTTCCGCACCGCCGCCGGGATCACTCTCGGCATGGACGAGCTGT
ACAAGCCGGTCACCGGCGTGATGGAGAAGCCTAGTCCCCTGCTGGTCGGGCGGGAATTTGTGAGACAG
TATTACACACTGCTGAACCAGGCCCCAGACATGCTGCATAGATTTTATGGAAAGAACTCTTCTTATGT
CCATGGGGGATTGGATTCAAATGGAAAGCCAGCAGATGCAGTCTACGGACAGAAAGAAATCCACAGGA
AAGTGATGTCACAAAACTTCACCAACTGCCACACCAAGATTCGCCATGTTGATGCTCATGCCACGCTA
AATGATGGTGTGGTAGTCCAGGTGATGGGGCTTCTCTCTAACAACAACCAGGCTTTGAGGAGATTCAT
GCAAACGTTTGTCCTTGCTCCTGAGGGGTCTGTTGCAAATAAATTCTATGTTCACAATGATATCTTCA
GATACCAAGATGAGGTCTTTGGTGGGTTTGTCACTGAGCCTCAGGAGGAGTCTGAAGAAGAAGTAGAG
GAACCTGAAGAAAGACAGCAAACACCTGAGGTGGTACCTGATGATTCTGGAACTTTCTATGATCAGGC
AGTTGTCAGTAATGACATGGAAGAACATTTAGAGGAGCCTGTTGCTGAACCAGAGCCTGATCCTGAAC
CAGAACCAGAACAAGAACCTGTATCTGAAATCCAAGAGGAAAAGCCTGAGCCAGTATTAGAAGAAACT
GCCCCTGAGGATGCTCAGAAGAGTTCTTCTCCAGCACCTGCAGACATAGCTCAGACAGTACAGGAAGA
CTTGAGGACATTTTCTTGGGCATCTGTGACCAGTAAGAATCTTCCACCCAGTGGAGCTGTTCCAGTTA
CTGGGATACCACCTCATGTTGTTAAAGTACCAGCTTCACAGCCCCGTCCAGAGTCTAAGCCTGAATCT
CAGATTCCACCACAAAGACCTCAGCGGGATCAAAGAGTGCGAGAACAACGAATAAATATTCCTCCCCA
AAGGGGACCCAGACCAATCCGTGAGGCTGGTGAGCAAGGTGACATTGAACCCCGAAGAATGGTGAGAC
ACCCTGACAGTCACCAACTCTTCATTGGCAACCTGCCTCATGAAGTGGACAAATCAGAGCTTAAAGAT
TTCTTTCAAAGTTATGGAAACGTGGTGGAGTTGCGCATTAACAGTGGTGGGAAATTACCCAATTTTGG
TTTTGTTGTGTTTGATGATTCTGAGCCTGTTCAGAAAGTCCTTAGCAACAGGCCCATCATGTTCAGAG
GTGAGGTCCGTCTGAATGTCGAAGAGAAGAAGACTCGAGCTGCCAGGGAAGGCGACCGACGAGATAAT
CGCCTTCGGGGACCTGGAGGCCCTCGAGGTGGGCTGGGTGGTGGAATGAGAGGCCCTCCCCGTGGAGG
CATGGTGCAGAAACCAGGATTTGGAGTGGGAAGGGGGCTTGCGCCACGGCAGTGAACCGGTTAATTCT
AGAATCGATGTAGGAGGTACTAAGCCGGTCATCATCACCATCACCATTGAGTTTAAACCCGCTGATCA
GCCTCGACTGTGCCTTCTAGTTGCCAGCCATCTGTTGTTTGCCCCTCCCCCGTGCCTTCCTTGACCCT
GGAAGGTGCCACTCCCACTGTCCTTTCCTAATAAAATGAGGAAATTGCATCGCATTGTCTGAGTAGGT
GTCATTCTATTCTGGGGGGTGGGGTGGGGCAGGACAGCAAGGGGGAGGATTGGGAAGACAATAGCAGG
CATGCTGGGGATGCGGTGGGCTCTATGGCGTGTGTCAGTTAGGGTGTGGAAAGTCCCCAGGCTCCCCA
GCAGGCAGAAGTATGCAAAGCATGCATCTCAATTAGTCAGCAACCAGGTGTGGAAAGTCCCCAGGCTC
CCCAGCAGGCAGAAGTATGCAAAGCATGCATCTCAATTAGTCAGCAACCATAGTCCCGCCCCTAACTC
CGCCCATCCCGCCCCTAACTCCGCCCAGTTCCGCCCATTCTCCGCCCCATGGCTGACTAATTTTTTTT
ATTTATGCAGAGGCCGAGGCCGCCTCTGCCTCTGAGCTATTCCAGAAGTAGTGAGGAGGCTTTTTTGG
AGGCCTAGGCTTTTGCAAAAAGCTCCCATGACCGAGTACAAGCCCACGGTGCGCCTCGCCACCCGCGA
CGACGTCCCCAGGGCCGTACGCACCCTCGCCGCCGCGTTCGCCGACTACCCCGCCACGCGCCACACCG
TCGATCCGGACCGCCACATCGAGCGGGTCACCGAGCTGCAAGAACTCTTCCTCACGCGCGTCGGGCTC
GACATCGGCAAGGTGTGGGTCGCGGACGACGGCGCCGCGGTGGCGGTCTGGACCACGCCGGAGAGCGT
CGAAGCGGGGGCGGTGTTCGCCGAGATCGGCCCGCGCATGGCCGAGTTGAGCGGTTCCCGGCTGGCCG
CGCAGCAACAGATGGAAGGCCTCCTGGCGCCGCACCGGCCCAAGGAGCCCGCGTGGTTCCTGGCCACC
GTCGGCGTCTCGCCCGACCACCAGGGCAAGGGTCTGGGCAGCGCCGTCGTGCTCCCCGGAGTGGAGGC
GGCCGAGCGCGCCGGGGTGCCCGCCTTCCTGGAGACCTCCGCGCCCCGCAACCTCCCCTTCTACGAGC
GGCTCGGCTTCACCGTCACCGCCGACGTCGAGGTGCCCGAAGGACCGCGCACCTGGTGCATGACCCGC
AAGCCCGGTGCCTGAGCGGGACTCTGGGGTTCGAAATGACCGACCAAGCGACGCCCGAAATGACCGAC
CAAGCGACGCCCAACCTGCCATCACGAGATTTCGATTCCACCGCCGCCTTCTATGAAAGGTTGGGCTT
CGGAATCGTTTTCCGGGACGCCGGCTGGATGATCCTCCAGCGCGGGGATCTCATGCTGGAGTTCTTCG
CCCACCCCAACTTGTTTATTGCAGCTTATAATGGTTACAAATAAAGCAATAGCATCACAAATTTCACA
AATAAAGCATTTTTTTCACTGCATTCTAGTTGTGGTTTGTCCAAACTCATCAATGTATCTTATCATGT
CTGTATACCGTCGACCTCTAGCTAGTCGAGTTAATTAACGAGAGCATAATATTGATATGTGCCAAAGT
TGTTTCTGACTGACTAATAAGTATAATTTGTTTCTATTATGTATAGGTTAAGCTAATTACTTATTTTA
TAATACAACATGACTGTTTTTAAAGTACAAAATAAGTTTATTTTTGTAAAAGAGAGAATGTTTAAAAG
TTTTGTTACTTTATAGAAGAAATTTTGAGTTTTTGTTTTTTTTTAATAAATAAATAAACATAAATAAA
TTGTTTGTTGAATTTATTATTAGTATGTAAGTGTAAATATAATAAAACTTAATATCTATTCAAATTAA
TAAATAAACCTCGATATACAGACCGATAAAACACATGCGTCAATTTTACGCATGATTATCTTTAACGT

ACGTCACAATATGATTATCTTTCTAGGGTTAAATAATAGTTTCTAATTTTTTTATTATTCAGCCTGCT
GTCGTGAATACCGAGCTCCAATTCGCCCTATAGTGAGTCGTATTACAATTCACTGGCCGTCGTTTTAC
AACGTCGTGACTGGGAAAACCCTGGCGTTACCCAACTTAATCGCCTTGCAGCACATCCCCCTTTCGCC
AGCTGGCGTAATAGCGAAGAGGCCCGCACCGATCGCCCTTCCCAACAGTTGCGCAGCCTGAATGGCGA
ATGGCGCGACGCGCCCTGTAGCGGCGCATTAAGCGCGGCGGGTGTGGTGGTTACGCGCAGCGTGACCG
CTACACTTGCCAGCGCCCTAGCGCCCGCTCCTTTCGCTTTCTTCCCTTCCTTTCTCGCCACGTTCGCC
GGCTTTCCCCGTCAAGCTCTAAATCGGGGGCTCCCTTTAGGGTTCCGATTTAGTGCTTTACGGCACCT
CGACCCCAAAAAACTTGATTAGGGTGATGGTTCACGTAGTGGGCCATCGCCCTGATAGACGGTTTTTC
GCCCTTTGACGTTGGAGTCCACGTTCTTTAATAGTGGACTCTTGTTCCAAACTGGAACAACACTCAAC
CCTATCTCGGTCTATTCTTTTGATTTATAAGGGATTTTGCCGATTTCGGCCTATTGGTTAAAAAATGA
GCTGATTTAACAAAAATTTAACGCGAATTTTAACAAAATATTAACGTTTACAATTTCC

PiggyBac-Puro_NLS-SNAPf-3xeGFP+NLS-CLIP-3xmCherry

CAGGTGGCACTTTTCGGGGAAATGTGCGCGGAACCCCTATTTGTTTATTTTTCTAAATACATTCAAAT
ATGTATCCGCTCATGAGACAATAACCCTGATAAATGCTTCAATAATATTGAAAAAGGAAGAGTATGAG
TATTCAACATTTCCGTGTCGCCCTTATTCCCTTTTTTGCGGCATTTTGCCTTCCTGTTTTTGCTCACC
CAGAAACGCTGGTGAAAGTAAAAGATGCTGAAGATCAGTTGGGTGCACGAGTGGGTTACATCGAACTG
GATCTCAACAGCGGTAAGATCCTTGAGAGTTTTCGCCCCGAAGAACGTTTTCCAATGATGAGCACTTT
TAAAGTTCTGCTATGTGGCGCGGTATTATCCCGTATTGACGCCGGGCAAGAGCAACTCGGTCGCCGCA
TACACTATTCTCAGAATGACTTGGTTGAGTACTCACCAGTCACAGAAAAGCATCTTACGGATGGCATG
ACAGTAAGAGAATTATGCAGTGCTGCCATAACCATGAGTGATAACACTGCGGCCAACTTACTTCTGAC
AACGATCGGAGGACCGAAGGAGCTAACCGCTTTTTTGCACAACATGGGGGATCATGTAACTCGCCTTG
ATCGTTGGGAACCGGAGCTGAATGAAGCCATACCAAACGACGAGCGTGACACCACGATGCCTGTAGCA
ATGGCAACAACGTTGCGCAAACTATTAACTGGCGAACTACTTACTCTAGCTTCCCGGCAACAATTAAT
AGACTGGATGGAGGCGGATAAAGTTGCAGGACCACTTCTGCGCTCGGCCCTTCCGGCTGGCTGGTTTA
TTGCTGATAAATCTGGAGCCGGTGAGCGTGGGTCTCGCGGTATCATTGCAGCACTGGGGCCAGATGGT
AAGCCCTCCCGTATCGTAGTTATCTACACGACGGGGAGTCAGGCAACTATGGATGAACGAAATAGACA
GATCGCTGAGATAGGTGCCTCACTGATTAAGCATTGGTAACTGTCAGACCAAGTTTACTCATATATAC
TTTAGATTGATTTAAAACTTCATTTTTAATTTAAAAGGATCTAGGTGAAGATCCTTTTTGATAATCTC
ATGACCAAAATCCCTTAACGTGAGTTTTCGTTCCACTGAGCGTCAGACCCCGTAGAAAAGATCAAAGG
ATCTTCTTGAGATCCTTTTTTTCTGCGCGTAATCTGCTGCTTGCAAACAAAAAAACCACCGCTACCAG
CGGTGGTTTGTTTGCCGGATCAAGAGCTACCAACTCTTTTTCCGAAGGTAACTGGCTTCAGCAGAGCG
CAGATACCAAATACTGTCCTTCTAGTGTAGCCGTAGTTAGGCCACCACTTCAAGAACTCTGTAGCACC
GCCTACATACCTCGCTCTGCTAATCCTGTTACCAGTGGCTGCTGCCAGTGGCGATAAGTCGTGTCTTA
CCGGGTTGGACTCAAGACGATAGTTACCGGATAAGGCGCAGCGGTCGGGCTGAACGGGGGGTTCGTGC
ACACAGCCCAGCTTGGAGCGAACGACCTACACCGAACTGAGATACCTACAGCGTGAGCTATGAGAAAG
CGCCACGCTTCCCGAAGGGAGAAAGGCGGACAGGTATCCGGTAAGCGGCAGGGTCGGAACAGGAGAGC
GCACGAGGGAGCTTCCAGGGGGAAACGCCTGGTATCTTTATAGTCCTGTCGGGTTTCGCCACCTCTGA
CTTGAGCGTCGATTTTTGTGATGCTCGTCAGGGGGGCGGAGCCTATGGAAAAACGCCAGCAACGCGGC
CTTTTTACGGTTCCTGGCCTTTTGCTGGCCTTTTGCTCACATGTTCTTTCCTGCGTTATCCCCTGATT
CTGTGGATAACCGTATTACCGCCTTTGAGTGAGCTGATACCGCTCGCCGCAGCCGAACGACCGAGCGC
AGCGAGTCAGTGAGCGAGGAAGCGGAAGAGCGCCCAATACGCAAACCGCCTCTCCCCGCGCGTTGGCC
GATTCATTAATGCAGCTGGCACGACAGGTTTCCCGACTGGAAAGCGGGCAGTGAGCGCAACGCAATTA
ATGTGAGTTAGCTCACTCATTAGGCACCCCAGGCTTTACACTTTATGCTTCCGGCTCGTATGTTGTGT
GGAATTGTGAGCGGATAACAATTTCACACAGGAAACAGCTATGACCATGATTACGCCAAGCTCGGAAT
TAACCCTCACTAAAGGGAACAAAAGCTGGGTACCTCGCGCGACTTGGTTTGCCATTCTTTAGCGCGCG
TCGCGTCACACAGCTTGGCCACAATGTGGTTTTTGTCAAACGAAGATTCTATGACGTGTTTAAAGTTT
AGGTCGAGTAAAGCGCAAATCTTTTTTAACCCTAGAAAGATAGTCTGCGTAAAATTGACGCATGCATT
CTTGAAATATTGCTCTCTCTTTCTAAATAGCGCGAATCCGTCGCTGTGCATTTAGGACATCTCAGTCG
CCGCTTGGAGCTCCCGTGAGGCGTGCTTGTCAATGCGGTAAGTGTCACTGATTTTGAACTATAACGAC
CGCGTGAGTCAAAATGACGCATGATTATCTTTTACGTGACTTTTAAGATTTAACTCATACGATAATTA
TATTGTTATTTCATGTTCTACTTACGTGATAACTTATTATATATATATTTTCTTGTTATAGATATCGT

GACTAATATATAATAAAATGGGTAGTTCTTTAGACGATGAGCATATCCTCTCTGCTCTTCTGCAAAGC
GATGACGAGCTTGTTGGCTAGCGCGCTGCTTCGCGATGTACGGGCCAGATATACGCGTTGACATTGAT
TATTGACTAGTAACTTGTTTATTGCAGCTTATAATGGTTACAAATAAGGCAATAGCATCACAAATTTC
ACAAATAAGGCATTTTTTTCACTGCATTCTAGTTTTGGTTTGTCCAAACTCATCAATGTATCTTATCA
TGTCTGGATCTCAAATCCCTCGGAAGCTGCGCCTGTCTTAGGTTGGAGTGATACATTTTTATCACTTT
TACCCGTCTTTGGATTAGGCAGTAGCTCTGACGGCCCTCCTGTCTTAGGTTAGTGAAAAATGTCACTC
TCTTACCCGTCATTGGCTGTCCAGCTTAGCTCGCAGGGGAGGTGGTCTGGGCCCATCGATTCTAGAAT
TAACCGGTGACCGGCTTGTACAGCTCGTCCATGCCGAGAGTGATCCCGGCGGCGGTCACGAACTCCAG
CAGGACCATGTGATCGCGCTTCTCGTTGGGGTCTTTGCTCAGGGCGGACTGGGTGCTCAGGTAGTGGT
TGTCGGGCAGCAGCACGGGGCCGTCGCCGATGGGGGTGTTCTGCTGGTAGTGGTCGGCGAGCTGCACG
CTGCCGTCCTCGATGTTGTGGCGGATCTTGAAGTTCACCTTGATGCCGTTCTTCTGCTTGTCGGCCAT
GATATAGACGTTGTGGCTGTTGTAGTTGTACTCCAGCTTGTGCCCCAGGATGTTGCCGTCCTCCTTGA
AGTCGATGCCCTTCAGCTCGATGCGGTTCACCAGGGTGTCGCCCTCGAACTTCACCTCGGCGCGGGTC
TTGTAGTTGCCGTCGTCCTTGAAGAAGATGGTGCGCTCCTGGACGTAGCCTTCGGGCATGGCGGACTT
GAAGAAGTCGTGCTGCTTCATGTGGTCGGGGTAGCGGCTGAAGCACTGCACGCCGTAGGTCAGGGTGG
TCACGAGGGTGGGCCAGGGCACGGGCAGCTTGCCGGTGGTGCAGATGAACTTCAGGGTCAGCTTGCCG
TAGGTGGCATCGCCCTCGCCCTCGCCGGACACGCTGAACTTGTGGCCGTTTACGTCGCCGTCCAGCTC
GACCAGGATGGGCACCACCCCGGTGAACAGCTCCTCGCCCTTGCTCACGCCGGTGACCGGCTTGTACA
GCTCGTCCATGCCGAGAGTGATCCCGGCGGCGGTCACGAACTCCAGCAGGACCATGTGATCGCGCTTC
TCGTTGGGGTCTTTGCTCAGGGCGGACTGGGTGCTCAGGTAGTGGTTGTCGGGCAGCAGCACGGGGCC
GTCGCCGATGGGGGTGTTCTGCTGGTAGTGGTCGGCGAGCTGCACGCTGCCGTCCTCGATGTTGTGGC
GGATCTTGAAGTTCACCTTGATGCCGTTCTTCTGCTTGTCGGCCATGATATAGACGTTGTGGCTGTTG
TAGTTGTACTCCAGCTTGTGCCCCAGGATGTTGCCGTCCTCCTTGAAGTCGATGCCCTTCAGCTCGAT
GCGGTTCACCAGGGTGTCGCCCTCGAACTTCACCTCGGCGCGGGTCTTGTAGTTGCCGTCGTCCTTGA
AGAAGATGGTGCGCTCCTGGACGTAGCCTTCGGGCATGGCGGACTTGAAGAAGTCGTGCTGCTTCATG
TGGTCGGGGTAGCGGCTGAAGCACTGCACGCCGTAGGTCAGGGTGGTCACGAGGGTGGGCCAGGGCAC
GGGCAGCTTGCCGGTGGTGCAGATGAACTTCAGGGTCAGCTTGCCGTAGGTGGCATCGCCCTCGCCCT
CGCCGGACACGCTGAACTTGTGGCCGTTTACGTCGCCGTCCAGCTCGACCAGGATGGGCACCACCCCG
GTGAACAGCTCCTCGCCCTTGCTCACGCCGGTGACCGGCTTGTACAGCTCGTCCATGCCGAGAGTGAT
CCCGGCGGCGGTCACGAACTCCAGCAGGACCATGTGATCGCGCTTCTCGTTGGGGTCTTTGCTCAGGG
CGGACTGGGTGCTCAGGTAGTGGTTGTCGGGCAGCAGCACGGGGCCGTCGCCGATGGGGGTGTTCTGC
TGGTAGTGGTCGGCGAGCTGCACGCTGCCGTCCTCGATGTTGTGGCGGATCTTGAAGTTCACCTTGAT
GCCGTTCTTCTGCTTGTCGGCCATGATATAGACGTTGTGGCTGTTGTAGTTGTACTCCAGCTTGTGCC
CCAGGATGTTGCCGTCCTCCTTGAAGTCGATGCCCTTCAGCTCGATGCGGTTCACCAGGGTGTCGCCC
TCGAACTTCACCTCGGCGCGGGTCTTGTAGTTGCCGTCGTCCTTGAAGAAGATGGTGCGCTCCTGGAC
GTAGCCTTCGGGCATGGCGGACTTGAAGAAGTCGTGCTGCTTCATGTGGTCGGGGTAGCGGCTGAAGC
ACTGCACGCCGTAGGTCAGGGTGGTCACGAGGGTGGGCCAGGGCACGGGCAGCTTGCCGGTGGTGCAG
ATGAACTTCAGGGTCAGCTTGCCGTAGGTGGCATCGCCCTCGCCCTCGCCGGACACGCTGAACTTGTG
GCCGTTTACGTCGCCGTCCAGCTCGACCAGGATGGGCACCACCCCGGTGAACAGCTCCTCGCCCTTGC
TCACGCCGGTCCCTGGCGCGCCTCCGCCTGCAGGACCCAGCCCAGGCTTGCCCAGTCTGTGGCCCTCG
TGGGCCAGCAGCCACTCTTTCACGGCGAGCCCGCCCTCGTAGCCCCCCACGTCCAGGTCGCCCTGCAC
CACCCGGTGGCAGGGGATCAGAATGGGCACGGGATTTCCGCTCAGGGCGGTTTTCACGGCGGCGGTGG
CGGCGGGATTGCCGGCCAGGGCGGCCAGGTGGCTGTAGCTGATGACCTCTCCGAACTTCACCACTTTC
AGCAGTTTCCACAGCACCTGGCGGGTAAAGCTCTCCTGCTGGAACACTGGGTGGTGCAGGGCTGGCAC
AGGGAACTCCTCGATGGCCTCAGGCTGGTGAAAGTAGGCGTTGAGCCAGGCGGTGGCCTGCATCAGTG
GCTCTGGTCCGCCCAGCACGGCGGCTGGGGCAGGCACTTCCACGGCGTCGGCGGCAGATGTTCCTTTG
CCCAGGAAGATGATACGGTGCAGGCCCTGTTCGCACCCAGACAGTTCCAGCTTGCCCAGAGGGCTATC
CAGGGTGGTGCGCTTCATTTCGCAGTCTTTGTCACCTGAGCCTGGAGCAACTTTTCTTTTCTTTTTTG
GTCCCATGCGGCCGCGGATCCCGCGTCCTAGGTTTCGACCGCGGAGGCTGGATCGGTCCCGGTGTCTT
CTATGGAGGTCAAAACAGCGTGGATGGCGTCTCCAGGCGATCTGACGGTTCACTAAACGAGCTCTGCT
TATATAGGCCTCCCACCGTACACGCCTACAAGCTTCTTTCACTTTTCTCTATCACTGATAGGGAGTGG
TAAACTCGACTTTCACTTTTCTCTATCACTGATAGGGAGTGGTAAACTCGACTTTCACTTTTCTCTAT
CACTGATAGGGAGTGGTAAACTCGACTTTCACTTTTCTCTATCACTGATAGGGAGTGGTAAACTCGAC
TTTCACTTTTCTCTATCACTGATAGGGAGTGGTAAACTCGACTTTCACTTTTCTCTATCACTGATAGG

GAGTGGTAAACTCGACTTTCACTTTTCTCTATCACTGATAGGGAGTGGTAAACTCGAGGGTAGGCGTG
TACGGTGGGAGGCCTATATAAGCAGAGCTCGTTTAGTGAACCGTCAGATCGCCTGGAGACGCCATCCA
CGCTGTTTTGACCTCCATAGAAGACACCGGGACCGATCCAGCCTCCGCGGAATTCTTAATAGCGGCCG
CATGGGACCAAAAAAGAAAAGAAAAGTTGCTCCAGGCTCAGGTGACAAAGACTGCGAAATGAAGCGCA
CCACCCTGGATAGCCCTCTGGGCAAGCTGGAACTGTCTGGGTGCGAACAGGGCCTGCACCGTATCATC
TTCCTGGGCAAAGGAACATCTGCCGCCGACGCCGTGGAAGTGCCTGCCCCAGCCGCCGTGCTGGGCGG
ACCAGAGCCACTGATCCAGGCCACCGCCTGGCTCAACGCCTACTTTCACCAGCCTGAGGCCATCGAGG
AGTTCCCTGTGCCAGCCCTGCACCACCCAGTGTTCCAGCAGGAGAGCTTTACCCGCCAGGTGCTGTGG
AAACTGCTGAAAGTGGTGAAGTTCGGAGAGGTCATCAGCGAGAGCCACCTGGCCGCCCTGGTGGGCAA
TCCCGCCGCCACCGCCGCCGTGAACACCGCCCTGGACGGAAATCCCGTGCCCATTCTGATCCCCTGCC
ACCGGGTGGTGCAGGGCGACAGCGACGTGGGGCCCTACCTGGGCGGGCTCGCCGTGAAAGAGTGGCTG
CTGGCCCACGAGGGCCACAGACTGGGCAAGCCTGGGCTGGGTCCTGCAGGCGGAGGCGCGCCAGGGAC
CGGCGTGAGCAAGGGCGAGGAGGATAACATGGCCATCATCAAGGAGTTCATGCGCTTCAAGGTGCACA
TGGAGGGCTCCGTGAACGGCCACGAGTTCGAGATCGAGGGCGAGGGCGAGGGCCGCCCCTACGAGGGC
ACCCAGACCGCCAAGCTGAAGGTGACCAAGGGTGGCCCCCTGCCCTTCGCCTGGGACATCCTGTCCCC
TCAGTTCATGTACGGCTCCAAGGCCTACGTGAAGCACCCCGCCGACATCCCCGACTACTTGAAGCTGT
CCTTCCCCGAGGGCTTCAAGTGGGAGCGCGTGATGAACTTCGAGGACGGCGGCGTGGTGACCGTGACC
CAGGACTCCTCCCTGCAGGACGGCGAGTTCATCTACAAGGTGAAGCTGCGCGGCACCAACTTCCCCTC
CGACGGCCCCGTAATGCAGAAGAAGACCATGGGCTGGGAGGCCTCCTCCGAGCGGATGTACCCCGAGG
ACGGCGCCCTGAAGGGCGAGATCAAGCAGAGGCTGAAGCTGAAGGACGGCGGCCACTACGACGCTGAG
GTCAAGACCACCTACAAGGCCAAGAAGCCCGTGCAGCTGCCCGGCGCCTACAACGTCAACATCAAGTT
GGACATCACCTCCCACAACGAGGACTACACCATCGTGGAACAGTACGAACGCGCCGAGGGCCGCCACT
CCACCGGCGGCATGGACGAGCTGTACAAGCCGGTCACCGGCGTGAGCAAGGGCGAGGAGGATAACATG
GCCATCATCAAGGAGTTCATGCGCTTCAAGGTGCACATGGAGGGCTCCGTGAACGGCCACGAGTTCGA
GATCGAGGGCGAGGGCGAGGGCCGCCCCTACGAGGGCACCCAGACCGCCAAGCTGAAGGTGACCAAGG
GTGGCCCCCTGCCCTTCGCCTGGGACATCCTGTCCCCTCAGTTCATGTACGGCTCCAAGGCCTACGTG
AAGCACCCCGCCGACATCCCCGACTACTTGAAGCTGTCCTTCCCCGAGGGCTTCAAGTGGGAGCGCGT
GATGAACTTCGAGGACGGCGGCGTGGTGACCGTGACCCAGGACTCCTCCCTGCAGGACGGCGAGTTCA
TCTACAAGGTGAAGCTGCGCGGCACCAACTTCCCCTCCGACGGCCCCGTAATGCAGAAGAAGACCATG
GGCTGGGAGGCCTCCTCCGAGCGGATGTACCCCGAGGACGGCGCCCTGAAGGGCGAGATCAAGCAGAG
GCTGAAGCTGAAGGACGGCGGCCACTACGACGCTGAGGTCAAGACCACCTACAAGGCCAAGAAGCCCG
TGCAGCTGCCCGGCGCCTACAACGTCAACATCAAGTTGGACATCACCTCCCACAACGAGGACTACACC
ATCGTGGAACAGTACGAACGCGCCGAGGGCCGCCACTCCACCGGCGGCATGGACGAGCTGTACAAGCC
GGTCACCGGCGTGAGCAAGGGCGAGGAGGATAACATGGCCATCATCAAGGAGTTCATGCGCTTCAAGG
TGCACATGGAGGGCTCCGTGAACGGCCACGAGTTCGAGATCGAGGGCGAGGGCGAGGGCCGCCCCTAC
GAGGGCACCCAGACCGCCAAGCTGAAGGTGACCAAGGGTGGCCCCCTGCCCTTCGCCTGGGACATCCT
GTCCCCTCAGTTCATGTACGGCTCCAAGGCCTACGTGAAGCACCCCGCCGACATCCCCGACTACTTGA
AGCTGTCCTTCCCCGAGGGCTTCAAGTGGGAGCGCGTGATGAACTTCGAGGACGGCGGCGTGGTGACC
GTGACCCAGGACTCCTCCCTGCAGGACGGCGAGTTCATCTACAAGGTGAAGCTGCGCGGCACCAACTT
CCCCTCCGACGGCCCCGTAATGCAGAAGAAGACCATGGGCTGGGAGGCCTCCTCCGAGCGGATGTACC
CCGAGGACGGCGCCCTGAAGGGCGAGATCAAGCAGAGGCTGAAGCTGAAGGACGGCGGCCACTACGAC
GCTGAGGTCAAGACCACCTACAAGGCCAAGAAGCCCGTGCAGCTGCCCGGCGCCTACAACGTCAACAT
CAAGTTGGACATCACCTCCCACAACGAGGACTACACCATCGTGGAACAGTACGAACGCGCCGAGGGCC
GCCACTCCACCGGCGGCATGGACGAGCTGTACAAGCCGGTCACCGGTTAATTCTAGAATCGATGTAGG
AGGTACTAAGCCGGTCATCATCACCATCACCATTGAGTTTAAACCCGCTGATCAGCCTCGACTGTGCC
TTCTAGTTGCCAGCCATCTGTTGTTTGCCCCTCCCCCGTGCCTTCCTTGACCCTGGAAGGTGCCACTC
CCACTGTCCTTTCCTAATAAAATGAGGAAATTGCATCGCATTGTCTGAGTAGGTGTCATTCTATTCTG
GGGGGTGGGGTGGGGCAGGACAGCAAGGGGGAGGATTGGGAAGACAATAGCAGGCATGCTGGGGATGC
GGTGGGCTCTATGGCGTGTGTCAGTTAGGGTGTGGAAAGTCCCCAGGCTCCCCAGCAGGCAGAAGTAT
GCAAAGCATGCATCTCAATTAGTCAGCAACCAGGTGTGGAAAGTCCCCAGGCTCCCCAGCAGGCAGAA
GTATGCAAAGCATGCATCTCAATTAGTCAGCAACCATAGTCCCGCCCCTAACTCCGCCCATCCCGCCC
CTAACTCCGCCCAGTTCCGCCCATTCTCCGCCCCATGGCTGACTAATTTTTTTTATTTATGCAGAGGC
CGAGGCCGCCTCTGCCTCTGAGCTATTCCAGAAGTAGTGAGGAGGCTTTTTTGGAGGCCTAGGCTTTT
GCAAAAAGCTCCCATGACCGAGTACAAGCCCACGGTGCGCCTCGCCACCCGCGACGACGTCCCCAGGG

CCGTACGCACCCTCGCCGCCGCGTTCGCCGACTACCCCGCCACGCGCCACACCGTCGATCCGGACCGC
CACATCGAGCGGGTCACCGAGCTGCAAGAACTCTTCCTCACGCGCGTCGGGCTCGACATCGGCAAGGT
GTGGGTCGCGGACGACGGCGCCGCGGTGGCGGTCTGGACCACGCCGGAGAGCGTCGAAGCGGGGGCGG
TGTTCGCCGAGATCGGCCCGCGCATGGCCGAGTTGAGCGGTTCCCGGCTGGCCGCGCAGCAACAGATG
GAAGGCCTCCTGGCGCCGCACCGGCCCAAGGAGCCCGCGTGGTTCCTGGCCACCGTCGGCGTCTCGCC
CGACCACCAGGGCAAGGGTCTGGGCAGCGCCGTCGTGCTCCCCGGAGTGGAGGCGGCCGAGCGCGCCG
GGGTGCCCGCCTTCCTGGAGACCTCCGCGCCCCGCAACCTCCCCTTCTACGAGCGGCTCGGCTTCACC
GTCACCGCCGACGTCGAGGTGCCCGAAGGACCGCGCACCTGGTGCATGACCCGCAAGCCCGGTGCCTG
AGCGGGACTCTGGGGTTCGAAATGACCGACCAAGCGACGCCCGAAATGACCGACCAAGCGACGCCCAA
CCTGCCATCACGAGATTTCGATTCCACCGCCGCCTTCTATGAAAGGTTGGGCTTCGGAATCGTTTTCC
GGGACGCCGGCTGGATGATCCTCCAGCGCGGGGATCTCATGCTGGAGTTCTTCGCCCACCCCAACTTG
TTTATTGCAGCTTATAATGGTTACAAATAAAGCAATAGCATCACAAATTTCACAAATAAAGCATTTTT
TTCACTGCATTCTAGTTGTGGTTTGTCCAAACTCATCAATGTATCTTATCATGTCTGTATACCGTCGA
CCTCTAGCTAGTCGAGTTAATTAACGAGAGCATAATATTGATATGTGCCAAAGTTGTTTCTGACTGAC
TAATAAGTATAATTTGTTTCTATTATGTATAGGTTAAGCTAATTACTTATTTTATAATACAACATGAC
TGTTTTTAAAGTACAAAATAAGTTTATTTTTGTAAAAGAGAGAATGTTTAAAAGTTTTGTTACTTTAT
AGAAGAAATTTTGAGTTTTTGTTTTTTTTTAATAAATAAATAAACATAAATAAATTGTTTGTTGAATT
TATTATTAGTATGTAAGTGTAAATATAATAAAACTTAATATCTATTCAAATTAATAAATAAACCTCGA
TATACAGACCGATAAAACACATGCGTCAATTTTACGCATGATTATCTTTAACGTACGTCACAATATGA
TTATCTTTCTAGGGTTAAATAATAGTTTCTAATTTTTTTATTATTCAGCCTGCTGTCGTGAATACCGA
GCTCCAATTCGCCCTATAGTGAGTCGTATTACAATTCACTGGCCGTCGTTTTACAACGTCGTGACTGG
GAAAACCCTGGCGTTACCCAACTTAATCGCCTTGCAGCACATCCCCCTTTCGCCAGCTGGCGTAATAG
CGAAGAGGCCCGCACCGATCGCCCTTCCCAACAGTTGCGCAGCCTGAATGGCGAATGGCGCGACGCGC
CCTGTAGCGGCGCATTAAGCGCGGCGGGTGTGGTGGTTACGCGCAGCGTGACCGCTACACTTGCCAGC
GCCCTAGCGCCCGCTCCTTTCGCTTTCTTCCCTTCCTTTCTCGCCACGTTCGCCGGCTTTCCCCGTCA
AGCTCTAAATCGGGGGCTCCCTTTAGGGTTCCGATTTAGTGCTTTACGGCACCTCGACCCCAAAAAAC
TTGATTAGGGTGATGGTTCACGTAGTGGGCCATCGCCCTGATAGACGGTTTTTCGCCCTTTGACGTTG
GAGTCCACGTTCTTTAATAGTGGACTCTTGTTCCAAACTGGAACAACACTCAACCCTATCTCGGTCTA
TTCTTTTGATTTATAAGGGATTTTGCCGATTTCGGCCTATTGGTTAAAAAATGAGCTGATTTAACAAA
AATTTAACGCGAATTTTAACAAAATATTAACGTTTACAATTTCC

PiggyBac-Puro_SNAPf-3xeGFP+NLS-CLIP-3xmCherry

CAGGTGGCACTTTTCGGGGAAATGTGCGCGGAACCCCTATTTGTTTATTTTTCTAAATACATTCAAAT
ATGTATCCGCTCATGAGACAATAACCCTGATAAATGCTTCAATAATATTGAAAAAGGAAGAGTATGAG
TATTCAACATTTCCGTGTCGCCCTTATTCCCTTTTTTGCGGCATTTTGCCTTCCTGTTTTTGCTCACC
CAGAAACGCTGGTGAAAGTAAAAGATGCTGAAGATCAGTTGGGTGCACGAGTGGGTTACATCGAACTG
GATCTCAACAGCGGTAAGATCCTTGAGAGTTTTCGCCCCGAAGAACGTTTTCCAATGATGAGCACTTT
TAAAGTTCTGCTATGTGGCGCGGTATTATCCCGTATTGACGCCGGGCAAGAGCAACTCGGTCGCCGCA
TACACTATTCTCAGAATGACTTGGTTGAGTACTCACCAGTCACAGAAAAGCATCTTACGGATGGCATG
ACAGTAAGAGAATTATGCAGTGCTGCCATAACCATGAGTGATAACACTGCGGCCAACTTACTTCTGAC
AACGATCGGAGGACCGAAGGAGCTAACCGCTTTTTTGCACAACATGGGGGATCATGTAACTCGCCTTG
ATCGTTGGGAACCGGAGCTGAATGAAGCCATACCAAACGACGAGCGTGACACCACGATGCCTGTAGCA
ATGGCAACAACGTTGCGCAAACTATTAACTGGCGAACTACTTACTCTAGCTTCCCGGCAACAATTAAT
AGACTGGATGGAGGCGGATAAAGTTGCAGGACCACTTCTGCGCTCGGCCCTTCCGGCTGGCTGGTTTA
TTGCTGATAAATCTGGAGCCGGTGAGCGTGGGTCTCGCGGTATCATTGCAGCACTGGGGCCAGATGGT
AAGCCCTCCCGTATCGTAGTTATCTACACGACGGGGAGTCAGGCAACTATGGATGAACGAAATAGACA
GATCGCTGAGATAGGTGCCTCACTGATTAAGCATTGGTAACTGTCAGACCAAGTTTACTCATATATAC
TTTAGATTGATTTAAAACTTCATTTTTAATTTAAAAGGATCTAGGTGAAGATCCTTTTTGATAATCTC
ATGACCAAAATCCCTTAACGTGAGTTTTCGTTCCACTGAGCGTCAGACCCCGTAGAAAAGATCAAAGG
ATCTTCTTGAGATCCTTTTTTTCTGCGCGTAATCTGCTGCTTGCAAACAAAAAAACCACCGCTACCAG
CGGTGGTTTGTTTGCCGGATCAAGAGCTACCAACTCTTTTTCCGAAGGTAACTGGCTTCAGCAGAGCG
CAGATACCAAATACTGTCCTTCTAGTGTAGCCGTAGTTAGGCCACCACTTCAAGAACTCTGTAGCACC

GCCTACATACCTCGCTCTGCTAATCCTGTTACCAGTGGCTGCTGCCAGTGGCGATAAGTCGTGTCTTA
CCGGGTTGGACTCAAGACGATAGTTACCGGATAAGGCGCAGCGGTCGGGCTGAACGGGGGGTTCGTGC
ACACAGCCCAGCTTGGAGCGAACGACCTACACCGAACTGAGATACCTACAGCGTGAGCTATGAGAAAG
CGCCACGCTTCCCGAAGGGAGAAAGGCGGACAGGTATCCGGTAAGCGGCAGGGTCGGAACAGGAGAGC
GCACGAGGGAGCTTCCAGGGGGAAACGCCTGGTATCTTTATAGTCCTGTCGGGTTTCGCCACCTCTGA
CTTGAGCGTCGATTTTTGTGATGCTCGTCAGGGGGGCGGAGCCTATGGAAAAACGCCAGCAACGCGGC
CTTTTTACGGTTCCTGGCCTTTTGCTGGCCTTTTGCTCACATGTTCTTTCCTGCGTTATCCCCTGATT
CTGTGGATAACCGTATTACCGCCTTTGAGTGAGCTGATACCGCTCGCCGCAGCCGAACGACCGAGCGC
AGCGAGTCAGTGAGCGAGGAAGCGGAAGAGCGCCCAATACGCAAACCGCCTCTCCCCGCGCGTTGGCC
GATTCATTAATGCAGCTGGCACGACAGGTTTCCCGACTGGAAAGCGGGCAGTGAGCGCAACGCAATTA
ATGTGAGTTAGCTCACTCATTAGGCACCCCAGGCTTTACACTTTATGCTTCCGGCTCGTATGTTGTGT
GGAATTGTGAGCGGATAACAATTTCACACAGGAAACAGCTATGACCATGATTACGCCAAGCTCGGAAT
TAACCCTCACTAAAGGGAACAAAAGCTGGGTACCTCGCGCGACTTGGTTTGCCATTCTTTAGCGCGCG
TCGCGTCACACAGCTTGGCCACAATGTGGTTTTTGTCAAACGAAGATTCTATGACGTGTTTAAAGTTT
AGGTCGAGTAAAGCGCAAATCTTTTTTAACCCTAGAAAGATAGTCTGCGTAAAATTGACGCATGCATT
CTTGAAATATTGCTCTCTCTTTCTAAATAGCGCGAATCCGTCGCTGTGCATTTAGGACATCTCAGTCG
CCGCTTGGAGCTCCCGTGAGGCGTGCTTGTCAATGCGGTAAGTGTCACTGATTTTGAACTATAACGAC
CGCGTGAGTCAAAATGACGCATGATTATCTTTTACGTGACTTTTAAGATTTAACTCATACGATAATTA
TATTGTTATTTCATGTTCTACTTACGTGATAACTTATTATATATATATTTTCTTGTTATAGATATCGT
GACTAATATATAATAAAATGGGTAGTTCTTTAGACGATGAGCATATCCTCTCTGCTCTTCTGCAAAGC
GATGACGAGCTTGTTGGCTAGCGCGCTGCTTCGCGATGTACGGGCCAGATATACGCGTTGACATTGAT
TATTGACTAGTAACTTGTTTATTGCAGCTTATAATGGTTACAAATAAGGCAATAGCATCACAAATTTC
ACAAATAAGGCATTTTTTTCACTGCATTCTAGTTTTGGTTTGTCCAAACTCATCAATGTATCTTATCA
TGTCTGGATCTCAAATCCCTCGGAAGCTGCGCCTGTCTTAGGTTGGAGTGATACATTTTTATCACTTT
TACCCGTCTTTGGATTAGGCAGTAGCTCTGACGGCCCTCCTGTCTTAGGTTAGTGAAAAATGTCACTC
TCTTACCCGTCATTGGCTGTCCAGCTTAGCTCGCAGGGGAGGTGGTCTGGGCCCATCGATTCTAGAAT
TAACCGGTGACCGGCTTGTACAGCTCGTCCATGCCGAGAGTGATCCCGGCGGCGGTCACGAACTCCAG
CAGGACCATGTGATCGCGCTTCTCGTTGGGGTCTTTGCTCAGGGCGGACTGGGTGCTCAGGTAGTGGT
TGTCGGGCAGCAGCACGGGGCCGTCGCCGATGGGGGTGTTCTGCTGGTAGTGGTCGGCGAGCTGCACG
CTGCCGTCCTCGATGTTGTGGCGGATCTTGAAGTTCACCTTGATGCCGTTCTTCTGCTTGTCGGCCAT
GATATAGACGTTGTGGCTGTTGTAGTTGTACTCCAGCTTGTGCCCCAGGATGTTGCCGTCCTCCTTGA
AGTCGATGCCCTTCAGCTCGATGCGGTTCACCAGGGTGTCGCCCTCGAACTTCACCTCGGCGCGGGTC
TTGTAGTTGCCGTCGTCCTTGAAGAAGATGGTGCGCTCCTGGACGTAGCCTTCGGGCATGGCGGACTT
GAAGAAGTCGTGCTGCTTCATGTGGTCGGGGTAGCGGCTGAAGCACTGCACGCCGTAGGTCAGGGTGG
TCACGAGGGTGGGCCAGGGCACGGGCAGCTTGCCGGTGGTGCAGATGAACTTCAGGGTCAGCTTGCCG
TAGGTGGCATCGCCCTCGCCCTCGCCGGACACGCTGAACTTGTGGCCGTTTACGTCGCCGTCCAGCTC
GACCAGGATGGGCACCACCCCGGTGAACAGCTCCTCGCCCTTGCTCACGCCGGTGACCGGCTTGTACA
GCTCGTCCATGCCGAGAGTGATCCCGGCGGCGGTCACGAACTCCAGCAGGACCATGTGATCGCGCTTC
TCGTTGGGGTCTTTGCTCAGGGCGGACTGGGTGCTCAGGTAGTGGTTGTCGGGCAGCAGCACGGGGCC
GTCGCCGATGGGGGTGTTCTGCTGGTAGTGGTCGGCGAGCTGCACGCTGCCGTCCTCGATGTTGTGGC
GGATCTTGAAGTTCACCTTGATGCCGTTCTTCTGCTTGTCGGCCATGATATAGACGTTGTGGCTGTTG
TAGTTGTACTCCAGCTTGTGCCCCAGGATGTTGCCGTCCTCCTTGAAGTCGATGCCCTTCAGCTCGAT
GCGGTTCACCAGGGTGTCGCCCTCGAACTTCACCTCGGCGCGGGTCTTGTAGTTGCCGTCGTCCTTGA
AGAAGATGGTGCGCTCCTGGACGTAGCCTTCGGGCATGGCGGACTTGAAGAAGTCGTGCTGCTTCATG
TGGTCGGGGTAGCGGCTGAAGCACTGCACGCCGTAGGTCAGGGTGGTCACGAGGGTGGGCCAGGGCAC
GGGCAGCTTGCCGGTGGTGCAGATGAACTTCAGGGTCAGCTTGCCGTAGGTGGCATCGCCCTCGCCCT
CGCCGGACACGCTGAACTTGTGGCCGTTTACGTCGCCGTCCAGCTCGACCAGGATGGGCACCACCCCG
GTGAACAGCTCCTCGCCCTTGCTCACGCCGGTGACCGGCTTGTACAGCTCGTCCATGCCGAGAGTGAT
CCCGGCGGCGGTCACGAACTCCAGCAGGACCATGTGATCGCGCTTCTCGTTGGGGTCTTTGCTCAGGG
CGGACTGGGTGCTCAGGTAGTGGTTGTCGGGCAGCAGCACGGGGCCGTCGCCGATGGGGGTGTTCTGC
TGGTAGTGGTCGGCGAGCTGCACGCTGCCGTCCTCGATGTTGTGGCGGATCTTGAAGTTCACCTTGAT
GCCGTTCTTCTGCTTGTCGGCCATGATATAGACGTTGTGGCTGTTGTAGTTGTACTCCAGCTTGTGCC
CCAGGATGTTGCCGTCCTCCTTGAAGTCGATGCCCTTCAGCTCGATGCGGTTCACCAGGGTGTCGCCC
TCGAACTTCACCTCGGCGCGGGTCTTGTAGTTGCCGTCGTCCTTGAAGAAGATGGTGCGCTCCTGGAC

GTAGCCTTCGGGCATGGCGGACTTGAAGAAGTCGTGCTGCTTCATGTGGTCGGGGTAGCGGCTGAAGC
ACTGCACGCCGTAGGTCAGGGTGGTCACGAGGGTGGGCCAGGGCACGGGCAGCTTGCCGGTGGTGCAG
ATGAACTTCAGGGTCAGCTTGCCGTAGGTGGCATCGCCCTCGCCCTCGCCGGACACGCTGAACTTGTG
GCCGTTTACGTCGCCGTCCAGCTCGACCAGGATGGGCACCACCCCGGTGAACAGCTCCTCGCCCTTGC
TCACGCCGGTCCCTGGCGCGCCTCCGCCTGCAGGACCCAGCCCAGGCTTGCCCAGTCTGTGGCCCTCG
TGGGCCAGCAGCCACTCTTTCACGGCGAGCCCGCCCTCGTAGCCCCCCACGTCCAGGTCGCCCTGCAC
CACCCGGTGGCAGGGGATCAGAATGGGCACGGGATTTCCGCTCAGGGCGGTTTTCACGGCGGCGGTGG
CGGCGGGATTGCCGGCCAGGGCGGCCAGGTGGCTGTAGCTGATGACCTCTCCGAACTTCACCACTTTC
AGCAGTTTCCACAGCACCTGGCGGGTAAAGCTCTCCTGCTGGAACACTGGGTGGTGCAGGGCTGGCAC
AGGGAACTCCTCGATGGCCTCAGGCTGGTGAAAGTAGGCGTTGAGCCAGGCGGTGGCCTGCATCAGTG
GCTCTGGTCCGCCCAGCACGGCGGCTGGGGCAGGCACTTCCACGGCGTCGGCGGCAGATGTTCCTTTG
CCCAGGAAGATGATACGGTGCAGGCCCTGTTCGCACCCAGACAGTTCCAGCTTGCCCAGAGGGCTATC
CAGGGTGGTGCGCTTCATTTCGCAGTCTTTGTCGCCGGCCATGCGGCCGCCGCGTCCTAGGTTTCGAC
CGCGGAGGCTGGATCGGTCCCGGTGTCTTCTATGGAGGTCAAAACAGCGTGGATGGCGTCTCCAGGCG
ATCTGACGGTTCACTAAACGAGCTCTGCTTATATAGGCCTCCCACCGTACACGCCTACAAGCTTCTTT
CACTTTTCTCTATCACTGATAGGGAGTGGTAAACTCGACTTTCACTTTTCTCTATCACTGATAGGGAG
TGGTAAACTCGACTTTCACTTTTCTCTATCACTGATAGGGAGTGGTAAACTCGACTTTCACTTTTCTC
TATCACTGATAGGGAGTGGTAAACTCGACTTTCACTTTTCTCTATCACTGATAGGGAGTGGTAAACTC
GACTTTCACTTTTCTCTATCACTGATAGGGAGTGGTAAACTCGACTTTCACTTTTCTCTATCACTGAT
AGGGAGTGGTAAACTCGAGGGTAGGCGTGTACGGTGGGAGGCCTATATAAGCAGAGCTCGTTTAGTGA
ACCGTCAGATCGCCTGGAGACGCCATCCACGCTGTTTTGACCTCCATAGAAGACACCGGGACCGATCC
AGCCTCCGCGGAATTCTTAATAGCGGCCGCATGGGACCAAAAAAGAAAAGAAAAGTTGCTCCAGGCTC
AGGTGACAAAGACTGCGAAATGAAGCGCACCACCCTGGATAGCCCTCTGGGCAAGCTGGAACTGTCTG
GGTGCGAACAGGGCCTGCACCGTATCATCTTCCTGGGCAAAGGAACATCTGCCGCCGACGCCGTGGAA
GTGCCTGCCCCAGCCGCCGTGCTGGGCGGACCAGAGCCACTGATCCAGGCCACCGCCTGGCTCAACGC
CTACTTTCACCAGCCTGAGGCCATCGAGGAGTTCCCTGTGCCAGCCCTGCACCACCCAGTGTTCCAGC
AGGAGAGCTTTACCCGCCAGGTGCTGTGGAAACTGCTGAAAGTGGTGAAGTTCGGAGAGGTCATCAGC
GAGAGCCACCTGGCCGCCCTGGTGGGCAATCCCGCCGCCACCGCCGCCGTGAACACCGCCCTGGACGG
AAATCCCGTGCCCATTCTGATCCCCTGCCACCGGGTGGTGCAGGGCGACAGCGACGTGGGGCCCTACC
TGGGCGGGCTCGCCGTGAAAGAGTGGCTGCTGGCCCACGAGGGCCACAGACTGGGCAAGCCTGGGCTG
GGTCCTGCAGGCGGAGGCGCGCCAGGGACCGGCGTGAGCAAGGGCGAGGAGGATAACATGGCCATCAT
CAAGGAGTTCATGCGCTTCAAGGTGCACATGGAGGGCTCCGTGAACGGCCACGAGTTCGAGATCGAGG
GCGAGGGCGAGGGCCGCCCCTACGAGGGCACCCAGACCGCCAAGCTGAAGGTGACCAAGGGTGGCCCC
CTGCCCTTCGCCTGGGACATCCTGTCCCCTCAGTTCATGTACGGCTCCAAGGCCTACGTGAAGCACCC
CGCCGACATCCCCGACTACTTGAAGCTGTCCTTCCCCGAGGGCTTCAAGTGGGAGCGCGTGATGAACT
TCGAGGACGGCGGCGTGGTGACCGTGACCCAGGACTCCTCCCTGCAGGACGGCGAGTTCATCTACAAG
GTGAAGCTGCGCGGCACCAACTTCCCCTCCGACGGCCCCGTAATGCAGAAGAAGACCATGGGCTGGGA
GGCCTCCTCCGAGCGGATGTACCCCGAGGACGGCGCCCTGAAGGGCGAGATCAAGCAGAGGCTGAAGC
TGAAGGACGGCGGCCACTACGACGCTGAGGTCAAGACCACCTACAAGGCCAAGAAGCCCGTGCAGCTG
CCCGGCGCCTACAACGTCAACATCAAGTTGGACATCACCTCCCACAACGAGGACTACACCATCGTGGA
ACAGTACGAACGCGCCGAGGGCCGCCACTCCACCGGCGGCATGGACGAGCTGTACAAGCCGGTCACCG
GCGTGAGCAAGGGCGAGGAGGATAACATGGCCATCATCAAGGAGTTCATGCGCTTCAAGGTGCACATG
GAGGGCTCCGTGAACGGCCACGAGTTCGAGATCGAGGGCGAGGGCGAGGGCCGCCCCTACGAGGGCAC
CCAGACCGCCAAGCTGAAGGTGACCAAGGGTGGCCCCCTGCCCTTCGCCTGGGACATCCTGTCCCCTC
AGTTCATGTACGGCTCCAAGGCCTACGTGAAGCACCCCGCCGACATCCCCGACTACTTGAAGCTGTCC
TTCCCCGAGGGCTTCAAGTGGGAGCGCGTGATGAACTTCGAGGACGGCGGCGTGGTGACCGTGACCCA
GGACTCCTCCCTGCAGGACGGCGAGTTCATCTACAAGGTGAAGCTGCGCGGCACCAACTTCCCCTCCG
ACGGCCCCGTAATGCAGAAGAAGACCATGGGCTGGGAGGCCTCCTCCGAGCGGATGTACCCCGAGGAC
GGCGCCCTGAAGGGCGAGATCAAGCAGAGGCTGAAGCTGAAGGACGGCGGCCACTACGACGCTGAGGT
CAAGACCACCTACAAGGCCAAGAAGCCCGTGCAGCTGCCCGGCGCCTACAACGTCAACATCAAGTTGG
ACATCACCTCCCACAACGAGGACTACACCATCGTGGAACAGTACGAACGCGCCGAGGGCCGCCACTCC
ACCGGCGGCATGGACGAGCTGTACAAGCCGGTCACCGGCGTGAGCAAGGGCGAGGAGGATAACATGGC
CATCATCAAGGAGTTCATGCGCTTCAAGGTGCACATGGAGGGCTCCGTGAACGGCCACGAGTTCGAGA
TCGAGGGCGAGGGCGAGGGCCGCCCCTACGAGGGCACCCAGACCGCCAAGCTGAAGGTGACCAAGGGT

GGCCCCCTGCCCTTCGCCTGGGACATCCTGTCCCCTCAGTTCATGTACGGCTCCAAGGCCTACGTGAA
GCACCCCGCCGACATCCCCGACTACTTGAAGCTGTCCTTCCCCGAGGGCTTCAAGTGGGAGCGCGTGA
TGAACTTCGAGGACGGCGGCGTGGTGACCGTGACCCAGGACTCCTCCCTGCAGGACGGCGAGTTCATC
TACAAGGTGAAGCTGCGCGGCACCAACTTCCCCTCCGACGGCCCCGTAATGCAGAAGAAGACCATGGG
CTGGGAGGCCTCCTCCGAGCGGATGTACCCCGAGGACGGCGCCCTGAAGGGCGAGATCAAGCAGAGGC
TGAAGCTGAAGGACGGCGGCCACTACGACGCTGAGGTCAAGACCACCTACAAGGCCAAGAAGCCCGTG
CAGCTGCCCGGCGCCTACAACGTCAACATCAAGTTGGACATCACCTCCCACAACGAGGACTACACCAT
CGTGGAACAGTACGAACGCGCCGAGGGCCGCCACTCCACCGGCGGCATGGACGAGCTGTACAAGCCGG
TCACCGGTTAATTCTAGAATCGATGTAGGAGGTACTAAGCCGGTCATCATCACCATCACCATTGAGTT
TAAACCCGCTGATCAGCCTCGACTGTGCCTTCTAGTTGCCAGCCATCTGTTGTTTGCCCCTCCCCCGT
GCCTTCCTTGACCCTGGAAGGTGCCACTCCCACTGTCCTTTCCTAATAAAATGAGGAAATTGCATCGC
ATTGTCTGAGTAGGTGTCATTCTATTCTGGGGGGTGGGGTGGGGCAGGACAGCAAGGGGGAGGATTGG
GAAGACAATAGCAGGCATGCTGGGGATGCGGTGGGCTCTATGGCGTGTGTCAGTTAGGGTGTGGAAAG
TCCCCAGGCTCCCCAGCAGGCAGAAGTATGCAAAGCATGCATCTCAATTAGTCAGCAACCAGGTGTGG
AAAGTCCCCAGGCTCCCCAGCAGGCAGAAGTATGCAAAGCATGCATCTCAATTAGTCAGCAACCATAG
TCCCGCCCCTAACTCCGCCCATCCCGCCCCTAACTCCGCCCAGTTCCGCCCATTCTCCGCCCCATGGC
TGACTAATTTTTTTTATTTATGCAGAGGCCGAGGCCGCCTCTGCCTCTGAGCTATTCCAGAAGTAGTG
AGGAGGCTTTTTTGGAGGCCTAGGCTTTTGCAAAAAGCTCCCATGACCGAGTACAAGCCCACGGTGCG
CCTCGCCACCCGCGACGACGTCCCCAGGGCCGTACGCACCCTCGCCGCCGCGTTCGCCGACTACCCCG
CCACGCGCCACACCGTCGATCCGGACCGCCACATCGAGCGGGTCACCGAGCTGCAAGAACTCTTCCTC
ACGCGCGTCGGGCTCGACATCGGCAAGGTGTGGGTCGCGGACGACGGCGCCGCGGTGGCGGTCTGGAC
CACGCCGGAGAGCGTCGAAGCGGGGGCGGTGTTCGCCGAGATCGGCCCGCGCATGGCCGAGTTGAGCG
GTTCCCGGCTGGCCGCGCAGCAACAGATGGAAGGCCTCCTGGCGCCGCACCGGCCCAAGGAGCCCGCG
TGGTTCCTGGCCACCGTCGGCGTCTCGCCCGACCACCAGGGCAAGGGTCTGGGCAGCGCCGTCGTGCT
CCCCGGAGTGGAGGCGGCCGAGCGCGCCGGGGTGCCCGCCTTCCTGGAGACCTCCGCGCCCCGCAACC
TCCCCTTCTACGAGCGGCTCGGCTTCACCGTCACCGCCGACGTCGAGGTGCCCGAAGGACCGCGCACC
TGGTGCATGACCCGCAAGCCCGGTGCCTGAGCGGGACTCTGGGGTTCGAAATGACCGACCAAGCGACG
CCCGAAATGACCGACCAAGCGACGCCCAACCTGCCATCACGAGATTTCGATTCCACCGCCGCCTTCTA
TGAAAGGTTGGGCTTCGGAATCGTTTTCCGGGACGCCGGCTGGATGATCCTCCAGCGCGGGGATCTCA
TGCTGGAGTTCTTCGCCCACCCCAACTTGTTTATTGCAGCTTATAATGGTTACAAATAAAGCAATAGC
ATCACAAATTTCACAAATAAAGCATTTTTTTCACTGCATTCTAGTTGTGGTTTGTCCAAACTCATCAA
TGTATCTTATCATGTCTGTATACCGTCGACCTCTAGCTAGTCGAGTTAATTAACGAGAGCATAATATT
GATATGTGCCAAAGTTGTTTCTGACTGACTAATAAGTATAATTTGTTTCTATTATGTATAGGTTAAGC
TAATTACTTATTTTATAATACAACATGACTGTTTTTAAAGTACAAAATAAGTTTATTTTTGTAAAAGA
GAGAATGTTTAAAAGTTTTGTTACTTTATAGAAGAAATTTTGAGTTTTTGTTTTTTTTTAATAAATAA
ATAAACATAAATAAATTGTTTGTTGAATTTATTATTAGTATGTAAGTGTAAATATAATAAAACTTAAT
ATCTATTCAAATTAATAAATAAACCTCGATATACAGACCGATAAAACACATGCGTCAATTTTACGCAT
GATTATCTTTAACGTACGTCACAATATGATTATCTTTCTAGGGTTAAATAATAGTTTCTAATTTTTTT
ATTATTCAGCCTGCTGTCGTGAATACCGAGCTCCAATTCGCCCTATAGTGAGTCGTATTACAATTCAC
TGGCCGTCGTTTTACAACGTCGTGACTGGGAAAACCCTGGCGTTACCCAACTTAATCGCCTTGCAGCA
CATCCCCCTTTCGCCAGCTGGCGTAATAGCGAAGAGGCCCGCACCGATCGCCCTTCCCAACAGTTGCG
CAGCCTGAATGGCGAATGGCGCGACGCGCCCTGTAGCGGCGCATTAAGCGCGGCGGGTGTGGTGGTTA
CGCGCAGCGTGACCGCTACACTTGCCAGCGCCCTAGCGCCCGCTCCTTTCGCTTTCTTCCCTTCCTTT
CTCGCCACGTTCGCCGGCTTTCCCCGTCAAGCTCTAAATCGGGGGCTCCCTTTAGGGTTCCGATTTAG
TGCTTTACGGCACCTCGACCCCAAAAAACTTGATTAGGGTGATGGTTCACGTAGTGGGCCATCGCCCT
GATAGACGGTTTTTCGCCCTTTGACGTTGGAGTCCACGTTCTTTAATAGTGGACTCTTGTTCCAAACT
GGAACAACACTCAACCCTATCTCGGTCTATTCTTTTGATTTATAAGGGATTTTGCCGATTTCGGCCTA
TTGGTTAAAAAATGAGCTGATTTAACAAAAATTTAACGCGAATTTTAACAAAATATTAACGTTTACAA
TTTCC

Xlone-Puro_SNAP-CLIP-display

ATCACCTCGAGTTTACTCCCTATCAGTGATAGAGAACGTATGAAGAGTTTACTCCCTATCAGTGATAG
AGAACGTATGCAGACTTTACTCCCTATCAGTGATAGAGAACGTATAAGGAGTTTACTCCCTATCAGTG
ATAGAGAACGTATGACCAGTTTACTCCCTATCAGTGATAGAGAACGTATCTACAGTTTACTCCCTATC
AGTGATAGAGAACGTATATCCAGTTTACTCCCTATCAGTGATAGAGAACGTATAAGCTTTGCTTATGT
AAACCAGGGCGCCTATAAAAGAGTGCTGATTTTTTGAGTAAACTTCAATTCCACAACACTTTTGTCTT
ATACCAACTTTCCGTACCACTTCCTACCCTCGTAAAGGTACCGAGCTCGGATCCACTAGTAACGGCCG
CCAGTGTGCTGGAATTCGGCTTGGGGATATCCACCATGGAGACAGACACACTCCTGCTATGGGTACTG
CTGCTCTGGGTTCCAGGTTCCACTGGTGACTATCCATATGATGTTCCAGATTATGCTGGGGCCCAGCC
GGCCAGATCTATGGATAAAGACTGCGAAATGAAACGCACTACCCTGGACAGCCCGCTGGGTAAACTGG
AACTGAGCGGTTGCGAGCAGGGCCTGCACGAAATCATTTTCCTGGGCAAAGGTACGAGCGCTGCGGAT
GCGGTCGAAGTACCGGCTCCGGCTGCGGTTCTGGGTGGCCCGGAGCCGCTGATGCAGGCTACCGCATG
GCTGAACGCATATTTTCACCAGCCAGAGGCGATTGAAGAGTTCCCTGTACCGGCCCTGCATCACCCGG
TATTTCAGCAGGAATCCTTTACCCGTCAGGTACTGTGGAAACTGCTGAAAGTAGTGAAATTCGGCGAG
GTGATCTCCTATTCCCATCTGGCGGCGCTGGCTGGCAACCCAGCGGCAACTGCGGCGGTGAAAACCGC
TCTGTCTGGTAATCCGGTGCCAATTCTGATCCCGTGCCACCGTGTGGTTCAGGGCGACCTGGACGTAG
GTGGCTACGAAGGCGGCCTGGCTGTGAAAGAATGGCTGCTGGCGCACGAAGGTCACCGTCTGGGTAAA
CCAGGCCTGGGCGGCCGCCTGGAAGTTCTGTTCCAGGGCCCGAAAGCTTTCCTCGAGATGGACAAGGA
TTGTGAGATGAAGCGTACCACTCTGGATTCTCCACTGGGCAAGCTGGAGCTGTCTGGCTGTGAACAAG
GTCTGCATGAGATTATCTTTCTGGGTAAGGGCACCTCCGCGGCGGATGCTGTTGAGGTTCCAGCGCCA
GCGGCTGTGCTGGGCGGTCCAGAACCACTGATCCAAGCGACTGCGTGGCTGAATGCGTACTTCCATCA
ACCGGAAGCTATCGAGGAATTTCCGGTTCCAGCGCTGCACCATCCAGTTTTCCAACAAGAGAGCTTCA
CTCGCCAAGTTCTGTGGAAGCTGCTGAAGGTTGTTAAGTTTGGTGAAGTTATTAGCGAGAGCCACCTG
GCTGCTCTGGTTGGTAATCCGGCTGCGACCGCTGCTGTTAACACTGCGCTGGACGGCAACCCAGTTCC
GATCCTGATTCCATGTCATCGCGTTGTGCAAGGTGATTCCGATGTTGGCCCATATCTGGGTGGTCTGG
CGGTTAAGGAGTGGCTGCTGGCTCATGAGGGCCATCGCCTGGGCAAGCCGGGTCTGGGTGGAGCTCTG
GCCGGAGGCATGTCCCATCACTGGGGGTACGGCAAACACAACGGACCTGAGCACTGGCATAAGGACTT
CCCCATTGCCAAGGGAGAGCGCCAGTCCCCTGTTGACATCGACACTCATACAGCCAAGTATGACCCTT
CCCTGAAGCCCCTGTCTGTTTCCTATGATCAAGCAACTTCCCTGAGGATCCTCAACAATGGTCATGCT
TTCAACGTGGAGTTTGATGACTCTCAGGACAAAGCAGTGCTCAAGGGAGGACCCCTGGATGGCACTTA
CAGATTGATTCAGTTTCACTTTCACTGGGGTTCACTTGATGGACAAGGTTCAGAGCATACTGTGGATA
AAAAGAAATATGCTGCAGAACTTCACTTGGTTCACTGGAACACCAAATATGGGGATTTTGGGAAAGCT
GTGCAGCAACCTGATGGACTGGCCGTTCTAGGTATTTTTTTGAAGGTTGGCAGCGCTAAACCGGGCCT
TCAGAAAGTTGTTGACGTGCTGGATTCCATTAAAACAAAGGGCAAGAGTGCTGACTTCACTAACTTCG
ATCCTCGTGGCCTCCTTCCTGAATCCCTGGATTACTGGACCTACCCAGGCTCACTGACCACCCCTCCT
CTTCTGGAATGTGTGACCTGGATTGTGCTCAAGGAACCCATCAGCGTCAGCAGCGAGCAGGTGTTGAA
ATTCCGTAAACTTAACTTCAATGGGGAGGGTGAACCCGAAGAACTGATGGTGGACAACTGGCGCCCAG
CTCAGCCACTGAAGAACAGGCAAATCAAAGCTTCCTTCAAAGTCGACGAACAAAAACTCATCTCAGAA
GAGGATCTGAATGCTGTGGGCCAGGACACGCAGGAGGTCATCGTGGTGCCACACTCCTTGCCCTTTAA
GGTGGTGGTGATCTCAGCCATCCTGGCCCTGGTGGTGCTCACCATCATCTCCCTTATCATCCTCATCA
TGCTTTGGCAGAAGAAGCCACGTTAGTCTAGTAGACCACCTCCCCTGCGAGCTAAGCTGGACAGCCAA
TGACGGGTAAGAGAGTGACATTTTTCACTAACCTAAGACAGGAGGGCCGTCAGAGCTACTGCCTAATC
CAAAGACGGGTAAAAGTGATAAAAATGTATCACTCCAACCTAAGACAGGCGCAGCTTCCGAGGGATTT
GAGATCCAGACATGATAAGATACATTGATGAGTTTGGACAAACCAAAACTAGAATGCAGTGAAAAAAA
TGCCTTATTTGTGAAATTTGTGATGCTATTGCCTTATTTGTAACCATTATAAGCTGCAATAAACAAGT
TTGATATCTATAACAAGAAAATATATATATAATAAGTTATCACGTAAGTAGAACATGAAATAACAATA
TAATTATCGTATGAGTTAAATCTTAAAAGTCACGTAAAAGATAATCATGCGTCATTTTGACTCACGCG
GTCGTTATAGTTCAAAATCAGTGACACTTACCGCATTGACAAGCACGCCTCACGGGAGCTCCAAGCGG
CGACTGAGATGTCCTAAATGCACAGCGACGGATTCGCGCTATTTAGAAAGAGAGAGCAATATTTCAAG
AATGCATGCGTCAATTTTACGCAGACTATCTTTCTAGGGTTAAGAATTCACTGGCCGTCGTTTTACAA
CGTCGTGACTGGGAAAACCCTGGCGTTACCCAACTTAATCGCCTTGCAGCACATCCCCCTTTCGCCAG
CTGGCGTAATAGCGAAGAGGCCCGCACCGATCGCCCTTCCCAACAGTTGCGCAGCCTGAATGGCGAAT
GGCGCCTGATGCGGTATTTTCTCCTTACGCATCTGTGCGGTATTTCACACCGCATATGGTGCACTCTC

AGTACAATCTGCTCTGATGCCGCATAGTTAAGCCAGCCCCGACACCCGCCAACACCCGCTGACGCGCC
CTGACGGGCTTGTCTGCTCCCGGCATCCGCTTACAGACAAGCTGTGACCGTCTCCGGGAGCTGCATGT
GTCAGAGGTTTTCACCGTCATCACCGAAACGCGCGAGACGAAAGGGCCTCGTGATACGCCTATTTTTA
TAGGTTAATGTCATGATAATAATGGTTTCTTAGACGTCAGGTGGCACTTTTCGGGGAAATGTGCGCGG
AACCCCTATTTGTTTATTTTTCTAAATACATTCAAATATGTATCCGCTCATGAGACAATAACCCTGAT
AAATGCTTCAATAATATTGAAAAAGGAAGAGTATGAGTATTCAACATTTCCGTGTCGCCCTTATTCCC
TTTTTTGCGGCATTTTGCCTTCCTGTTTTTGCTCACCCAGAAACGCTGGTGAAAGTAAAAGATGCTGA
AGATCAGTTGGGTGCACGAGTGGGTTACATCGAACTGGATCTCAACAGCGGTAAGATCCTTGAGAGTT
TTCGCCCCGAAGAACGTTTTCCAATGATGAGCACTTTTAAAGTTCTGCTATGTGGCGCGGTATTATCC
CGTATTGACGCCGGGCAAGAGCAACTCGGTCGCCGCATACACTATTCTCAGAATGACTTGGTTGAGTA
CTCACCAGTCACAGAAAAGCATCTTACGGATGGCATGACAGTAAGAGAATTATGCAGTGCTGCCATAA
CCATGAGTGATAACACTGCGGCCAACTTACTTCTGACAACGATCGGAGGACCGAAGGAGCTAACCGCT
TTTTTGCACAACATGGGGGATCATGTAACTCGCCTTGATCGTTGGGAACCGGAGCTGAATGAAGCCAT
ACCAAACGACGAGCGTGACACCACGATGCCTGTAGCAATGGCAACAACGTTGCGCAAACTATTAACTG
GCGAACTACTTACTCTAGCTTCCCGGCAACAATTAATAGACTGGATGGAGGCGGATAAAGTTGCAGGA
CCACTTCTGCGCTCGGCCCTTCCGGCTGGCTGGTTTATTGCTGATAAATCTGGAGCCGGTGAGCGTGG
GTCTCGCGGTATCATTGCAGCACTGGGGCCAGATGGTAAGCCCTCCCGTATCGTAGTTATCTACACGA
CGGGGAGTCAGGCAACTATGGATGAACGAAATAGACAGATCGCTGAGATAGGTGCCTCACTGATTAAG
CATTGGTAACTGTCAGACCAAGTTTACTCATATATACTTTAGATTGATTTAAAACTTCATTTTTAATT
TAAAAGGATCTAGGTGAAGATCCTTTTTGATAATCTCATGACCAAAATCCCTTAACGTGAGTTTTCGT
TCCACTGAGCGTCAGACCCCGTAGAAAAGATCAAAGGATCTTCTTGAGATCCTTTTTTTCTGCGCGTA
ATCTGCTGCTTGCAAACAAAAAAACCACCGCTACCAGCGGTGGTTTGTTTGCCGGATCAAGAGCTACC
AACTCTTTTTCCGAAGGTAACTGGCTTCAGCAGAGCGCAGATACCAAATACTGTTCTTCTAGTGTAGC
CGTAGTTAGGCCACCACTTCAAGAACTCTGTAGCACCGCCTACATACCTCGCTCTGCTAATCCTGTTA
CCAGTGGCTGCTGCCAGTGGCGATAAGTCGTGTCTTACCGGGTTGGACTCAAGACGATAGTTACCGGA
TAAGGCGCAGCGGTCGGGCTGAACGGGGGGTTCGTGCACACAGCCCAGCTTGGAGCGAACGACCTACA
CCGAACTGAGATACCTACAGCGTGAGCTATGAGAAAGCGCCACGCTTCCCGAAGGGAGAAAGGCGGAC
AGGTATCCGGTAAGCGGCAGGGTCGGAACAGGAGAGCGCACGAGGGAGCTTCCAGGGGGAAACGCCTG
GTATCTTTATAGTCCTGTCGGGTTTCGCCACCTCTGACTTGAGCGTCGATTTTTGTGATGCTCGTCAG
GGGGGCGGAGCCTATGGAAAAACGCCAGCAACGCGGCCTTTTTACGGTTCCTGGCCTTTTGCTGGCCT
TTTGCTCACATGTTCTTTCCTGCGTTATCCCCTGATTCTGTGGATAACCGTATTACCGCCTTTGAGTG
AGCTGATACCGCTCGCCGCAGCCGAACGACCGAGCGCAGCGAGTCAGTGAGCGAGGAAGCGGAAGAGC
GCCCAATACGCAAACCGCCTCTCCCCGCGCGTTGGCCGATTCATTAATGCAGCTGGCACGACAGGTTT
CCCGACTGGAAAGCGGGCAGTGAGCGCAACGCAATTAATGTGAGTTAGCTCACTCATTAGGCACCCCA
GGCTTTACACTTTATGCTTCCGGCTCGTATGTTGTGTGGAATTGTGAGCGGATAACAATTTCACACAG
GAAACAGCTATGACCATGATTACGCCAAGGTCGACTTAACCCTAGAAAGATAATCATATTGTGACGTA
CGTTAAAGATAATCATGCGTAAAATTGACGCATGTGTTTTATCGGTCTGTATATCGAGGTTTATTTAT
TAATTTGAATAGATATTAAGTTTTATTATATTTACACTTACATACTAATAATAAATTCAACAAACAAT
TTATTTATGTTTATTTATTTATTAAAAAAAAACAAAAACTCAAAATTTCTTCTATAAAGTAACAAAAC
TTTTAGCAGTGAAAAAAATGCTTTATTTGTGAAATTTGTGATGCTATTGCTTTATTTGTAACCATTAT
AAGCTGCAATAAACAAGTTAACAACAACAATTGCATTCATTTTATGTTTCAGGTTCAGGGGGAGGTGT
GGGAGGTTTTTTAAAGCAAGTAAAACCTCTACAAATGTGGTATGGCTGATTATGATCCTCTGGAGATC
CTAGGCTAGGCACCGGGCTTGCGGGTCATGCACCAGGTGCGCGGTCCTTCGGGCACCTCGACGTCGGC
GGTGACGGTGAAGCCGAGCCGCTCGTAGAAGGGGAGGTTGCGGGGCGCGGAGGTCTCCAGGAAGGCGG
GCACCCCGGCGCGCTCGGCCGCCTCCACTCCGGGGAGCACGACGGCGCTGCCCAGACCCTTGCCCTGG
TGGTCGGGCGAGACGCCGACGGTGGCCAGGAACCACGCGGGCTCCTTGGGCCGGTGCGGCGCCAGGAG
GCCTTCCATCTGTTGCTGCGCGGCCAGCCGGGAACCGCTCAACTCGGCCATGCGCGGGCCGATCTCGG
CGAACACCGCCCCCGCTTCGACGCTCTCCGGCGTGGTCCAGACCGCCACCGCGGCGCCGTCGTCCGCG
ACCCACACCTTGCCGATGTCGAGCCCGACGCGCGTGAGGAAGAGTTCTTGCAGCTCGGTGACCCGCTC
GATGTGGCGGTCCGGATCGACGGTGTGGCGCGTGGCGGGGTAGTCGGCGAACGCGGCGGCGAGGGTGC
GTACGGCCCTGGGGACGTCGTCGCGGGTGGCGAGGCGCACCGTGGGCTTGTACTCGGTCATGGGGCCG
GGGTTCTCCTCCACGTCGCCGGCCTGCTTCAGCAGGCTGAAGTTGGTGGCGCCGCTGCCCCCGGGGAG
CATGTCAAGGTCAAAATCGTCAAGAGCGTCAGCAGGCAGCATATCAAGGTCAAAGTCGTCAAGGGCAT
CGGCTGGGAGCATGTCTAAGTCAAAATCGTCAAGGGCGTCGGTCGGCCCGCCGCTTTCGCACTTTAGC

TGTTTCTCCAGGCCACATATGATTAGTTCCAGGCCGAAAAGGAAGGCAGGTTCGGCTCCCTGCCGGTC
GAACAGCTCAATTGCTTGTTTCAGAAGTGGGGGCATAGAATCGGTGGTAGGTGTCTCTCTTTCCTCTT
TTGCTACTTGATGCTCCTGTTCCTCCAATACGCAGCCCAGTGTAAAGTGGCCCACGGCGGACAGAGCG
TACAGTGCGTTCTCCAGGGAGAAGCCTTGCTGACACAGGAACGCGAGCTGATTTTCCAGGGTTTCGTA
CTGTTTCTCTGTTGGGCGGGTGCCGAGATGCACTTTAGCCCCGTCGCGATGTGAGAGGAGAGCACAGC
GGTATGACTTGGCGTTGTTCCGCAGAAAGTCTTGCCATGACTCGCCTTCCAGGGGGCAGGAGTGGGTA
TGATGCCTGTCCAGCATCTCGATTGGCAGGGCATCGAGCAGGGCCCGCTTGTTCTTCACGTGCCAGTA
CAGGGTAGGCTGCTCAACTCCCAGCTTTTGAGCGAGTTTCCTTGTCGTCAGGCCTTCGATACCGACTC
CATTGAGTAATTCCAGAGCAGAGTTTATGACTTTGCTCTTGTCCAGTCTAGACATCTTATCGTCATCG
TCTTTGTAATCCATGGTGGCGGATCCCGCGTCACGACACCTGTGTTCTGGCGGCAAACCCGTTGCGAA
AAAGAACGTTCACGGCGACTACTGCACTTATATACGGTTCTCCCCCACCCTCGGGAAAAAGGCGGAGC
CAGTACACGACATCACTTTCCCAGTTTACCCCGCGCCACCTTCTCTAGGCACCGGTTCAATTGCCGAC
CCCTCCCCCCAACTTCTCGGGGACTGTGGGCGATGTGCGCTCTGCCCACTGACGGGCACCGGAGCCAC
TCGAGTGGAATT

pcDNA3.1_mBP

GACGGATCGGGAGATCTCCCGATCCCCTATGGTGCACTCTCAGTACAATCTGCTCTGATGCCGCATAG
TTAAGCCAGTATCTGCTCCCTGCTTGTGTGTTGGAGGTCGCTGAGTAGTGCGCGAGCAAAATTTAAGC
TACAACAAGGCAAGGCTTGACCGACAATTGCATGAAGAATCTGCTTAGGGTTAGGCGTTTTGCGCTGC
TTCGCGATGTACGGGCCAGATATACGCGTTGACATTGATTATTGACTAGTGATTATTGAGCCATAGAA
TTCGAGCTTGCATGCCTGCAGGTCGTTACATAACTTACGGTAAATGGCCCGCCTGGCTGACCGCCCAA
CGACCCCCGCCCATTGACGTCAATAATGACGTATGTTCCCATAGTAACGCCAATAGGGACTTTCCATT
GACGTCAATGGGTGGAGTATTTACGGTAAACTGCCCACTTGGCAGTACATCAAGTGTATCATATGCCA
AGTACGCCCCCTATTGACGTCAATGACGGTAAATGGCCCGCCTGGCATTATGCCCAGTACATGACCTT
ATGGGACTTTCCTACTTGGCAGTACATCTACGTATTAGTCATCGCTATTACCATGGTGATGCGGTTTT
GGCAGTACATCAATGGGCGTGGATAGCGGTTTGACTCACGGGGATTTCCAAGTCTCCACCCCATTGAC
GTCAATGGGAGTTTGTTTTGGCACCAAAATCAACGGGACTTTCCAAAATGTCGTAACAACTCCGCCCC
ATTGACGCAAATGGGCGGTAGGCGTGTACGGTGGGAGGTCTATATAAGCAGAGCTCGTTTAGTGAACC
GTCAGATCGCCTGGAGACGCCATCCACGCTGTTTTGACCTCCATAGAAGACACCGGGACCGATCCAGC
CTCCGGACTCTAGAGGATCCGGTACTCGAGGAACTGAAAAACCAGAAAGTTAACTGGTAAGTTTAGTC
TTTTTGTCTTTTATTTCAGGTCCCGGATCCGGTGGTGGTGCAAATCAAAGAACTGCTCCTCAGTGGAT
GTTGCCTTTACTTCTAGGCCTGTACGGAAGTGTTACTTCTGCTCTAAAAGCTGCGGAATTGTACCCAA
TTCGTTAAGGCCAAATTGGCCACCATGGGCTCTAGCCTGGACGACGAGCACATCCTGAGCGCCCTGCT
GCAGAGCGACGACGAACTGGTGGGCGAGGACAGCGACAGCGAGGTCAGCGACCACGTGTCCGAGGACG
ACGTGCAGTCCGACACCGAGGAAGCCTTCATCGACGAGGTGCACGAAGTGCAGCCTACCAGCAGCGGC
TCCGAGATCCTGGACGAGCAGAACGTGATCGAGCAGCCTGGCAGCTCCCTGGCCAGCAACAGAATCCT
GACCCTGCCCCAGAGAACCATCAGAGGCAAGAACAAGCACTGCTGGTCCACCTCCAAGAGCACCAGGC
GGAGCAGAGTGTCCGCCCTGAACATCGTGCGGAGCCAGAGGGGCCCCACCAGAATGTGCAGAAACATC
TACGACCCCCTGCTGTGCTTCAAGCTGTTCTTCACCGACGAGATCATCAGCGAGATCGTGAAGTGGAC
CAACGCCGAGATCAGCCTGAAGAGGCGGGAGAGCATGACCAGCGCCACCTTCAGAGACACCAACGAGG
ACGAGATCTACGCCTTCTTCGGCATCCTGGTGATGACCGCCGTGAGAAAGGACAACCACATGAGCACC
GACGACCTGTTCGACAGATCCCTGAGCATGGTGTACGTGTCCGTGATGAGCAGAGACAGATTCGACTT
CCTGATCAGATGCCTGAGAATGGACGACAAGAGCATCAGACCCACCCTGCGGGAGAACGACGTGTTCA
CCCCCGTGCGGAAGATCTGGGACCTGTTCATCCACCAGTGCATCCAGAACTACACCCCTGGCGCCCAC
CTGACCATCGATGAGCAGCTGCTGGGCTTCAGAGGCAGATGCCCCTTCAGAGTGTACATCCCCAACAA
GCCCAGCAAGTACGGCATCAAGATCCTGATGATGTGCGACAGCGGCACCAAGTACATGATCAACGGCA
TGCCCTACCTGGGCAGAGGCACCCAGACAAACGGCGTGCCCCTGGGCGAGTACTACGTGAAAGAACTG
AGCAAGCCTGTGCATGGCAGCTGCAGGAACATCACCTGCGACAACTGGTTCACCAGCATCCCCCTGGC
CAAGAACCTGCTGCAGGAACCCTACAAGCTGACCATCGTGGGCACCGTGCGGAGCAACAAGCGGGAGA
TCCCAGAGGTGCTGAAGAACAGCAGATCCAGACCTGTGGGAACAAGCATGTTCTGCTTCGACGGCCCC
CTGACCCTGGTGTCCTACAAGCCCAAGCCCGCCAAGATGGTGTACCTGCTGTCCAGCTGCGACGAGGA
CGCCAGCATCAACGAGAGCACCGGCAAGCCCCAGATGGTGATGTACTACAACCAGACCAAGGGCGGCG

TGGACACCCTGGACCAGATGTGCAGCGTGATGACCTGCAGCAGAAAGACCAACAGATGGCCCATGGCC
CTGCTGTACGGCATGATCAATATCGCCTGCATCAACAGCTTCATCATCTACAGCCACAACGTGTCCAG
CAAGGGCGAGAAGGTGCAGAGCCGGAAGAAATTCATGCGGAACCTGTACATGAGCCTGACCTCCAGCT
TCATGAGAAAGAGACTGGAAGCCCCCACCCTGAAGAGATACCTGCGGGACAACATCAGCAACATCCTG
CCCAAGGAAGTGCCAGGAACAAGCGACGACAGCACCGAGGAACCCGTGATGAAGAAGAGGACCTACTG
CACCTACTGTCCCAGCAAGATCAGAAGAAAGGCCAACGCCAGCTGCAAGAAATGCAAAAAAGTGATCT
GCCGGGAGCACAACATCGACATGTGCCAGAGCTGTTTCTGAGGCCGTAACGGCCGCCAGAATTGGGGA
TCCAGACATGATAAGATACATTGATGAGTTTGGACAAACCACAACTAGAATGCAGTGAAAAAAATGCT
TTATTTGTGAAATTTGTGATGCTATTGCTTTATTTGTAACCATTATAAGCTGCAATAAACAAGTTAAC
GCGGAAGCTTGGTACCGAGCTCGGATCCACTAGCGGCCGCTCGAGTCTAGAGGGCCCTTCGAACAAAA
ACTCATCTCAGAAGAGGATCTGAATATGCATACCGGTCATCATCACCATCACCATTGAGTTTAAACCC
GCTGATCAGCCTCGACTGTGCCTTCTAGTTGCCAGCCATCTGTTGTTTGCCCCTCCCCCGTGCCTTCC
TTGACCCTGGAAGGTGCCACTCCCACTGTCCTTTCCTAATAAAATGAGGAAATTGCATCGCATTGTCT
GAGTAGGTGTCATTCTATTCTGGGGGGTGGGGTGGGGCAGGACAGCAAGGGGGAGGATTGGGAAGACA
ATAGCAGGCATGCTGGGGATGCGGTGGGCTCTATGGCTTCTGAGGCGGAAAGAACCAGCTGGGGCTCT
AGGGGGTATCCCCACGCGCCCTGTAGCGGCGCATTAAGCGCGGCGGGTGTGGTGGTTACGCGCAGCGT
GACCGCTACACTTGCCAGCGCCCTAGCGCCCGCTCCTTTCGCTTTCTTCCCTTCCTTTCTCGCCACGT
TCGCCGGCTTTCCCCGTCAAGCTCTAAATCGGGGGCTCCCTTTAGGGTTCCGATTTAGTGCTTTACGG
CACCTCGACCCCAAAAAACTTGATTAGGGTGATGGTTCACGTAGTGGGCCATCGCCCTGATAGACGGT
TTTTCGCCCTTTGACGTTGGAGTCCACGTTCTTTAATAGTGGACTCTTGTTCCAAACTGGAACAACAC
TCAACCCTATCTCGGTCTATTCTTTTGATTTATAAGGGATTTTGCCGATTTCGGCCTATTGGTTAAAA
AATGAGCTGATTTAACAAAAATTTAACGCGAATTAATTCTGTGGAATGTGTGTCAGTTAGGGTGTGGA
AAGTCCCCAGGCTCCCCAGCAGGCAGAAGTATGCAAAGCATGCATCTCAATTAGTCAGCAACCAGGTG
TGGAAAGTCCCCAGGCTCCCCAGCAGGCAGAAGTATGCAAAGCATGCATCTCAATTAGTCAGCAACCA
TAGTCCCGCCCCTAACTCCGCCCATCCCGCCCCTAACTCCGCCCAGTTCCGCCCATTCTCCGCCCCAT
GGCTGACTAATTTTTTTTATTTATGCAGAGGCCGAGGCCGCCTCTGCCTCTGAGCTATTCCAGAAGTA
GTGAGGAGGCTTTTTTGGAGGCCTAGGCTTTTGCAAAAAGCTCCCGGGAGCTTGTATATCCATTTTCG
GATCTGATCAAGAGACAGGATGAGGATCGTTTCGCATGATTGAACAAGATGGATTGCACGCAGGTTCT
CCGGCCGCTTGGGTGGAGAGGCTATTCGGCTATGACTGGGCACAACAGACAATCGGCTGCTCTGATGC
CGCCGTGTTCCGGCTGTCAGCGCAGGGGCGCCCGGTTCTTTTTGTCAAGACCGACCTGTCCGGTGCCC
TGAATGAACTGCAGGACGAGGCAGCGCGGCTATCGTGGCTGGCCACGACGGGCGTTCCTTGCGCAGCT
GTGCTCGACGTTGTCACTGAAGCGGGAAGGGACTGGCTGCTATTGGGCGAAGTGCCGGGGCAGGATCT
CCTGTCATCTCACCTTGCTCCTGCCGAGAAAGTATCCATCATGGCTGATGCAATGCGGCGGCTGCATA
CGCTTGATCCGGCTACCTGCCCATTCGACCACCAAGCGAAACATCGCATCGAGCGAGCACGTACTCGG
ATGGAAGCCGGTCTTGTCGATCAGGATGATCTGGACGAAGAGCATCAGGGGCTCGCGCCAGCCGAACT
GTTCGCCAGGCTCAAGGCGCGCATGCCCGACGGCGAGGATCTCGTCGTGACCCATGGCGATGCCTGCT
TGCCGAATATCATGGTGGAAAATGGCCGCTTTTCTGGATTCATCGACTGTGGCCGGCTGGGTGTGGCG
GACCGCTATCAGGACATAGCGTTGGCTACCCGTGATATTGCTGAAGAGCTTGGCGGCGAATGGGCTGA
CCGCTTCCTCGTGCTTTACGGTATCGCCGCTCCCGATTCGCAGCGCATCGCCTTCTATCGCCTTCTTG
ACGAGTTCTTCTGAGCGGGACTCTGGGGTTCGAAATGACCGACCAAGCGACGCCCAACCTGCCATCAC
GAGATTTCGATTCCACCGCCGCCTTCTATGAAAGGTTGGGCTTCGGAATCGTTTTCCGGGACGCCGGC
TGGATGATCCTCCAGCGCGGGGATCTCATGCTGGAGTTCTTCGCCCACCCCAACTTGTTTATTGCAGC
TTATAATGGTTACAAATAAAGCAATAGCATCACAAATTTCACAAATAAAGCATTTTTTTCACTGCATT
CTAGTTGTGGTTTGTCCAAACTCATCAATGTATCTTATCATGTCTGTATACCGTCGACCTCTAGCTAG
AGCTTGGCGTAATCATGGTCATAGCTGTTTCCTGTGTGAAATTGTTATCCGCTCACAATTCCACACAA
CATACGAGCCGGAAGCATAAAGTGTAAAGCCTGGGGTGCCTAATGAGTGAGCTAACTCACATTAATTG
CGTTGCGCTCACTGCCCGCTTTCCAGTCGGGAAACCTGTCGTGCCAGCTGCATTAATGAATCGGCCAA
CGCGCGGGGAGAGGCGGTTTGCGTATTGGGCGCTCTTCCGCTTCCTCGCTCACTGACTCGCTGCGCTC
GGTCGTTCGGCTGCGGCGAGCGGTATCAGCTCACTCAAAGGCGGTAATACGGTTATCCACAGAATCAG
GGGATAACGCAGGAAAGAACATGTGAGCAAAAGGCCAGCAAAAGGCCAGGAACCGTAAAAAGGCCGCG
TTGCTGGCGTTTTTCCATAGGCTCCGCCCCCCTGACGAGCATCACAAAAATCGACGCTCAAGTCAGAG
GTGGCGAAACCCGACAGGACTATAAAGATACCAGGCGTTTCCCCCTGGAAGCTCCCTCGTGCGCTCTC
CTGTTCCGACCCTGCCGCTTACCGGATACCTGTCCGCCTTTCTCCCTTCGGGAAGCGTGGCGCTTTCT
CATAGCTCACGCTGTAGGTATCTCAGTTCGGTGTAGGTCGTTCGCTCCAAGCTGGGCTGTGTGCACGA

ACCCCCCGTTCAGCCCGACCGCTGCGCCTTATCCGGTAACTATCGTCTTGAGTCCAACCCGGTAAGAC
ACGACTTATCGCCACTGGCAGCAGCCACTGGTAACAGGATTAGCAGAGCGAGGTATGTAGGCGGTGCT
ACAGAGTTCTTGAAGTGGTGGCCTAACTACGGCTACACTAGAAGAACAGTATTTGGTATCTGCGCTCT
GCTGAAGCCAGTTACCTTCGGAAAAAGAGTTGGTAGCTCTTGATCCGGCAAACAAACCACCGCTGGTA
GCGGTTTTTTTGTTTGCAAGCAGCAGATTACGCGCAGAAAAAAAGGATCTCAAGAAGATCCTTTGATC
TTTTCTACGGGGTCTGACGCTCAGTGGAACGAAAACTCACGTTAAGGGATTTTGGTCATGAGATTATC
AAAAAGGATCTTCACCTAGATCCTTTTAAATTAAAAATGAAGTTTTAAATCAATCTAAAGTATATATG
AGTAAACTTGGTCTGACAGTTACCAATGCTTAATCAGTGAGGCACCTATCTCAGCGATCTGTCTATTT
CGTTCATCCATAGTTGCCTGACTCCCCGTCGTGTAGATAACTACGATACGGGAGGGCTTACCATCTGG
CCCCAGTGCTGCAATGATACCGCGAGACCCACGCTCACCGGCTCCAGATTTATCAGCAATAAACCAGC
CAGCCGGAAGGGCCGAGCGCAGAAGTGGTCCTGCAACTTTATCCGCCTCCATCCAGTCTATTAATTGT
TGCCGGGAAGCTAGAGTAAGTAGTTCGCCAGTTAATAGTTTGCGCAACGTTGTTGCCATTGCTACAGG
CATCGTGGTGTCACGCTCGTCGTTTGGTATGGCTTCATTCAGCTCCGGTTCCCAACGATCAAGGCGAG
TTACATGATCCCCCATGTTGTGCAAAAAAGCGGTTAGCTCCTTCGGTCCTCCGATCGTTGTCAGAAGT
AAGTTGGCCGCAGTGTTATCACTCATGGTTATGGCAGCACTGCATAATTCTCTTACTGTCATGCCATC
CGTAAGATGCTTTTCTGTGACTGGTGAGTACTCAACCAAGTCATTCTGAGAATAGTGTATGCGGCGAC
CGAGTTGCTCTTGCCCGGCGTCAATACGGGATAATACCGCGCCACATAGCAGAACTTTAAAAGTGCTC
ATCATTGGAAAACGTTCTTCGGGGCGAAAACTCTCAAGGATCTTACCGCTGTTGAGATCCAGTTCGAT
GTAACCCACTCGTGCACCCAACTGATCTTCAGCATCTTTTACTTTCACCAGCGTTTCTGGGTGAGCAA
AAACAGGAAGGCAAAATGCCGCAAAAAAGGGAATAAGGGCGACACGGAAATGTTGAATACTCATACTC
TTCCTTTTTCAATATTATTGAAGCATTTATCAGGGTTATTGTCTCATGAGCGGATACATATTTGAATG
TATTTAGAAAAATAAACAAATAGGGGTTCCGCGCACATTTCCCCGAAAAGTGCCACCTGACGTC

Exemplary Sanger sequencing traces

Sanger sequencing trace obtained for the PCR product covering the Y701C site in STAT1 upon transfection of 0.5 pmol BG-Y701C and 2 pm NH-T288A guideRNA (compare figure 2d). Bystander-free editing is observed at nucleotide 112 (Y701 site, asterisk).

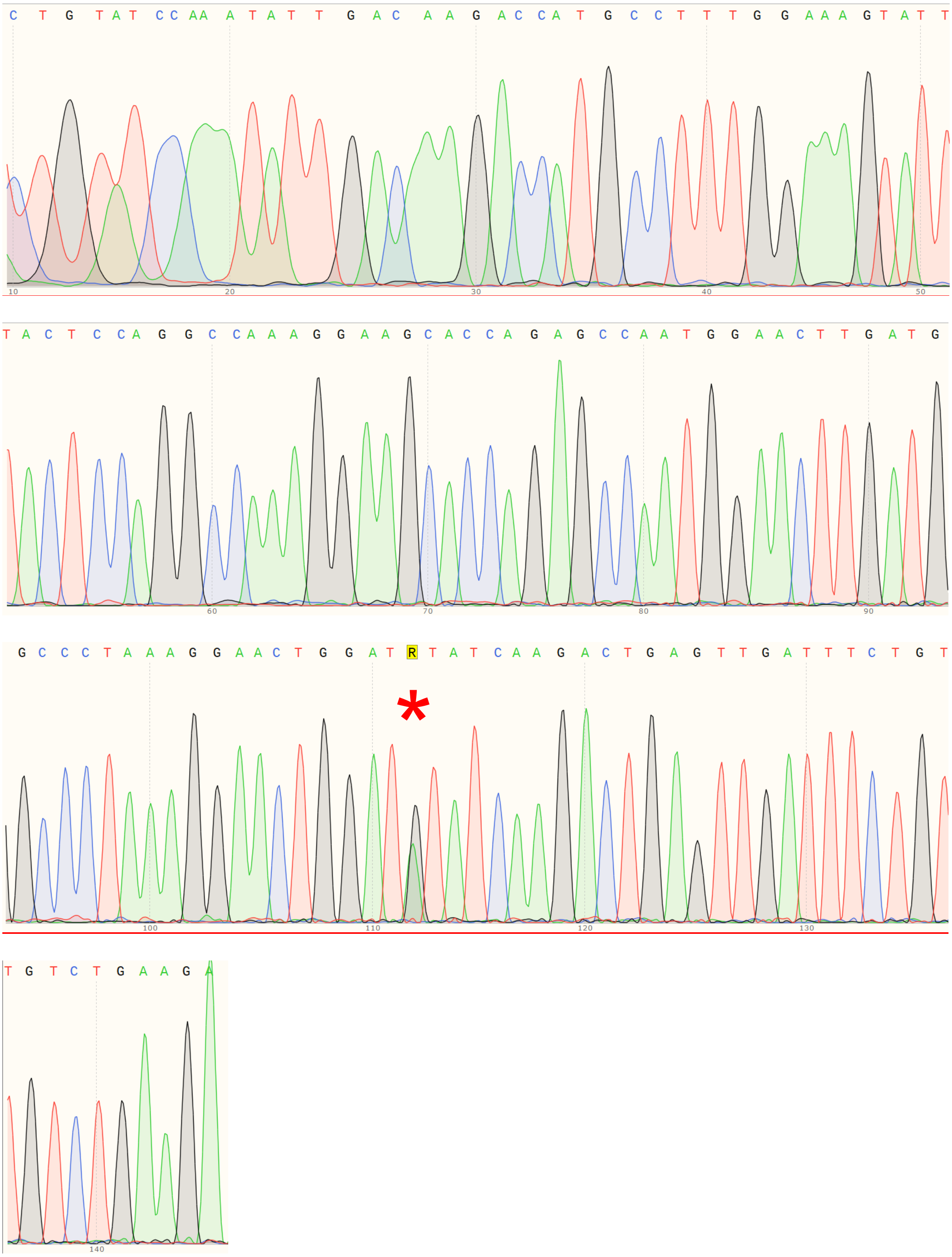

Sanger sequencing trace obtained for the PCR product covering the Y701C site in STAT1 upon transfection of 0.5 pmol NH-Y701C and 2 pm BG-T288A guideRNA (compare figure 2d). No editing is observed at nucleotide 112 (Y701 site, asterisk), as expected.

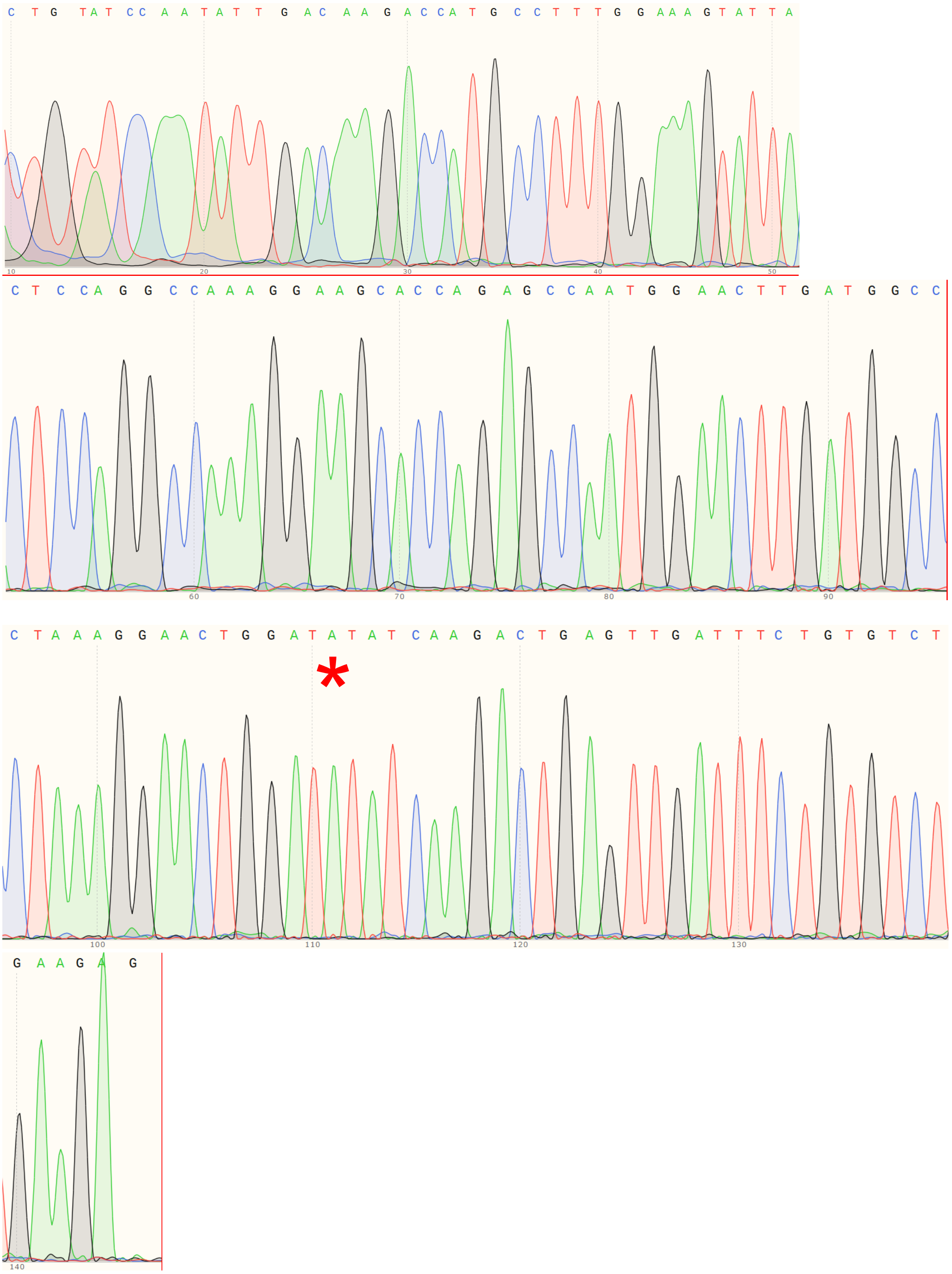

Sanger sequencing trace obtained for the PCR product covering T288A site in STAT1 upon transfection of 0.5 pmol NH-Y701C and 2 pmol BG-T288A guideRNAs (compare figure 2d). Bystander-free editing is observed at nucleotide 171 (T288A site, asterisk).

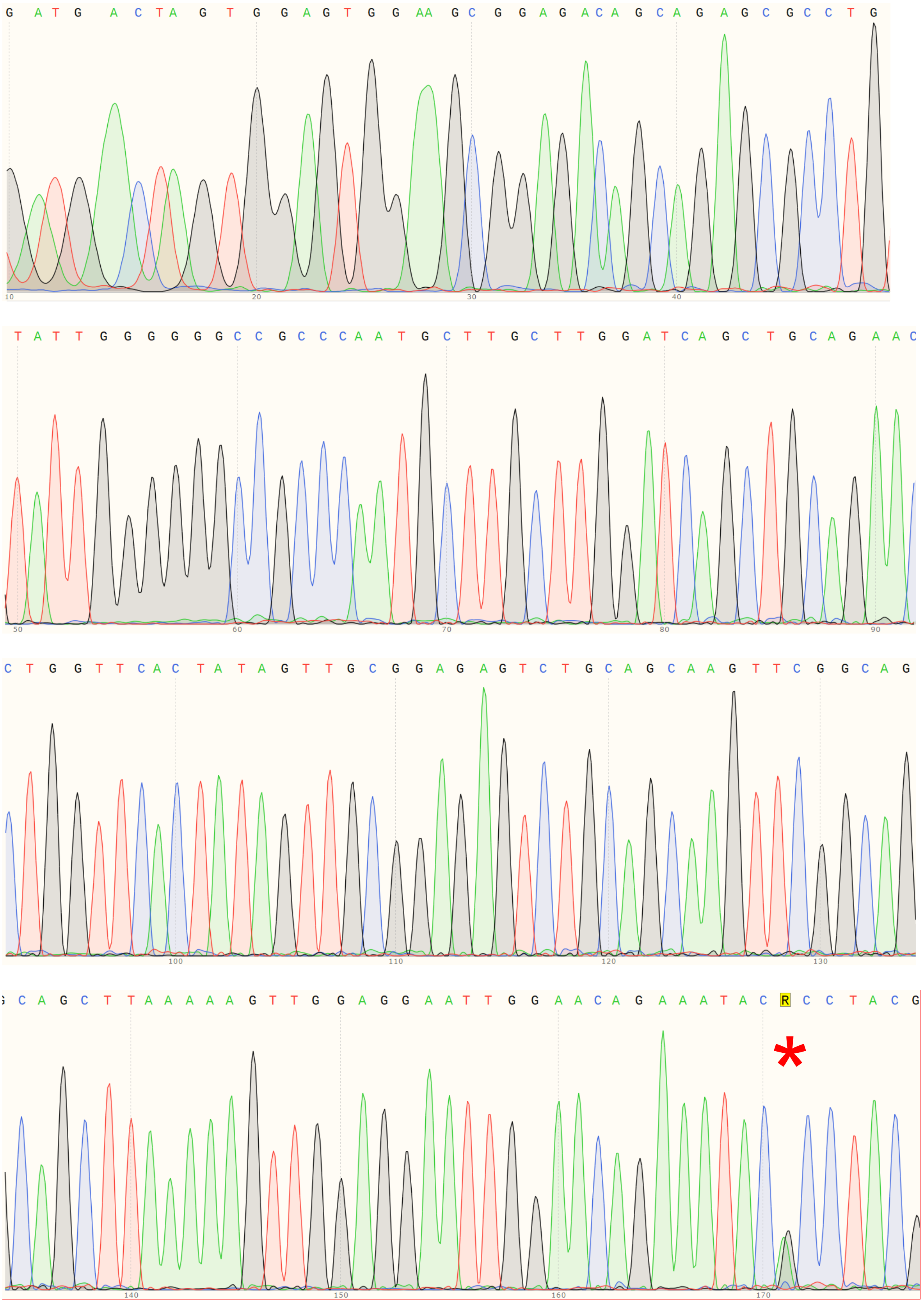

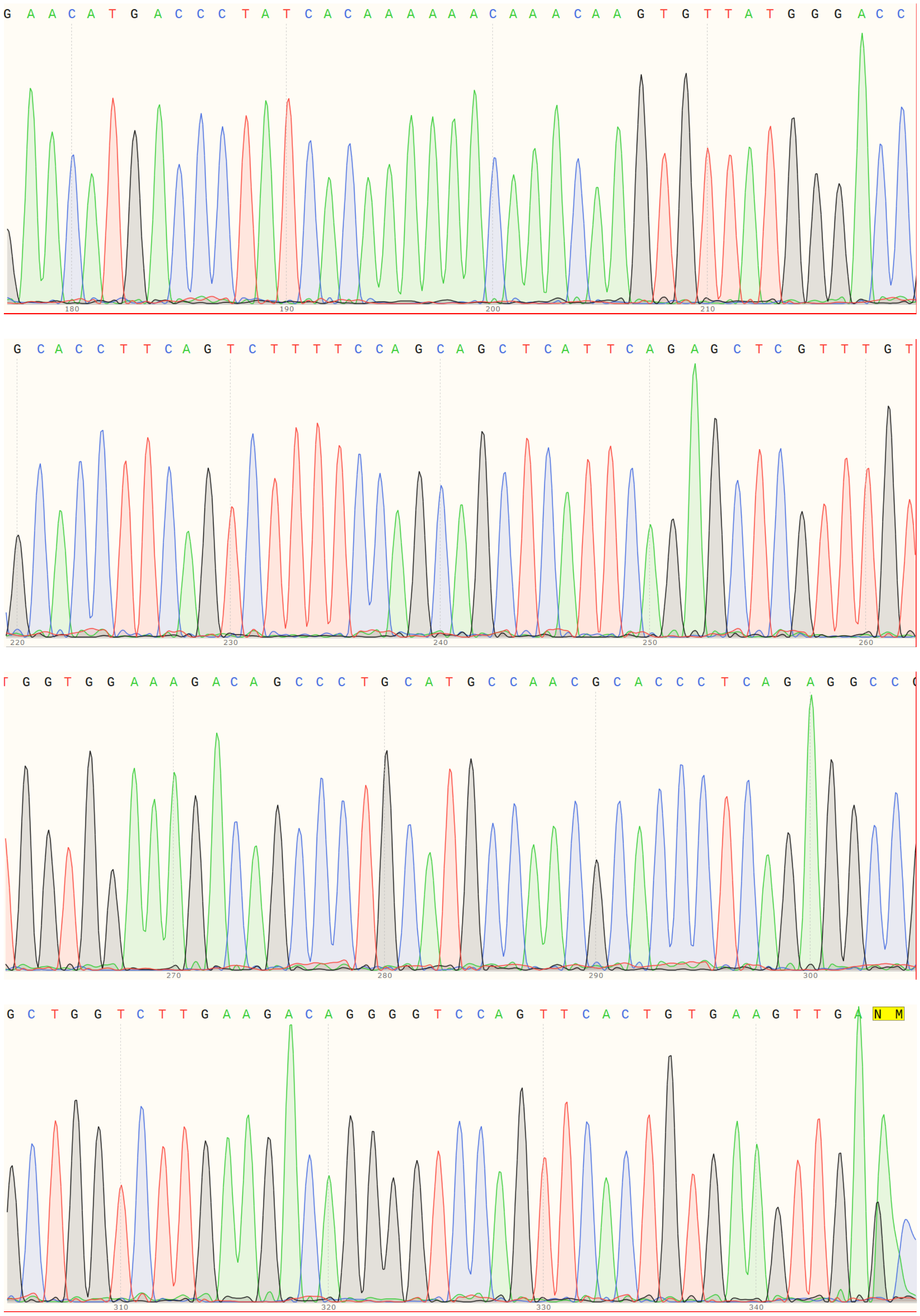
G A A C A T G A C C C T A T C A C A A A A A A C A A A C A A G T G T T A T G G G A C C
180
190
200
210
G C A C C T T C A G T C T T T T C C A G C A G C T C A T T C A G A G C T C G T T T G T
220
230
240
250
260
T G G T G G A A A G A C A G C C C T G C A T G C C A A C G C A C C C T C A G A G G C C
270
280
290
300
G C T G G T C T T G A A G A C A G G G G T C C A G T T C A C T G T G A A G T T G A N M
310
320
330
340

Sanger sequencing trace obtained for the PCR product covering T288A site in STAT1 upon transfection of 0.5 pmol NH-Y701C and 2 pmol BG-T288A guideRNAs (compare figure 2d). No editing is observed at nucleotide 171 (T288A site, asterisk), as expected.

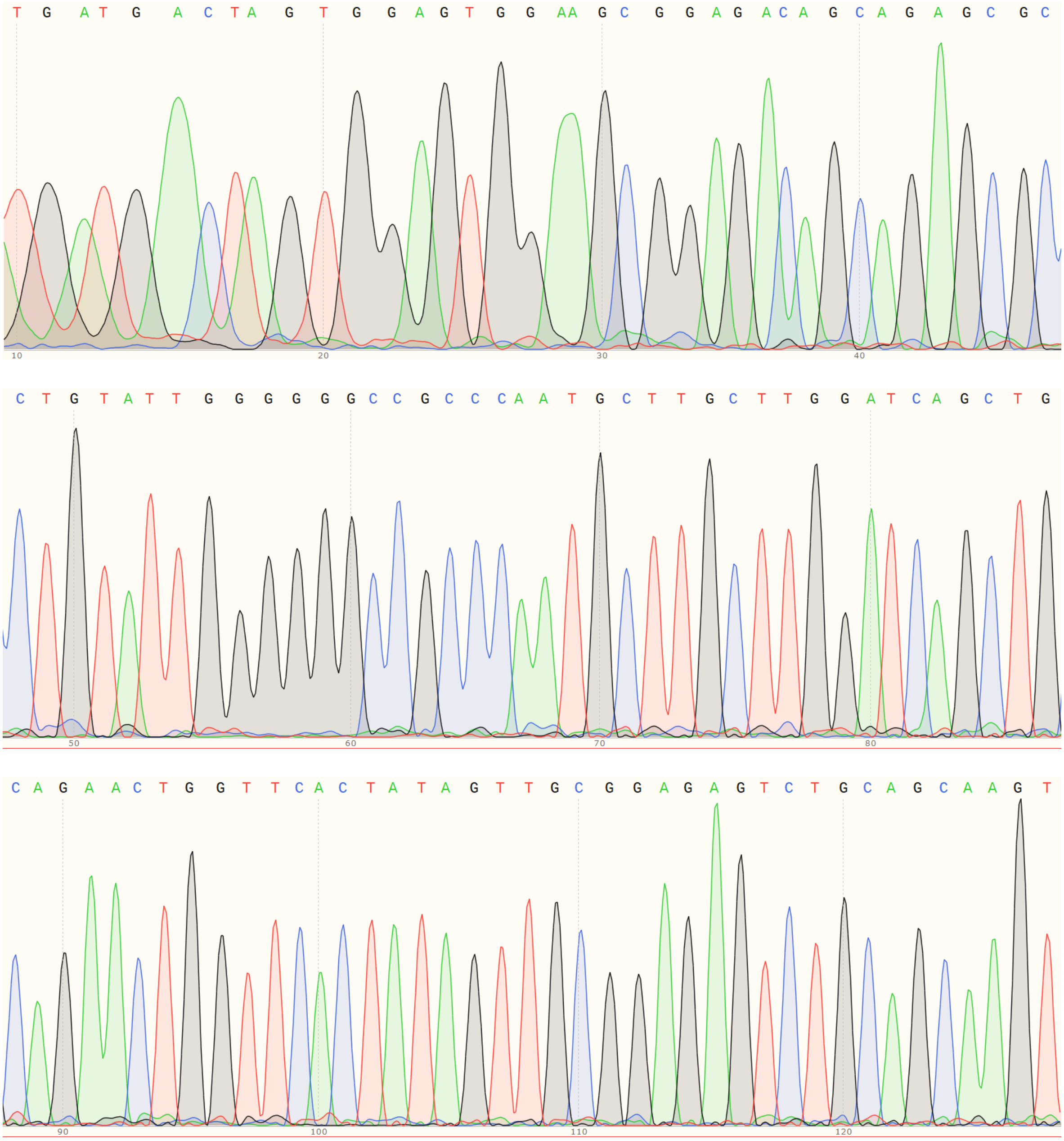

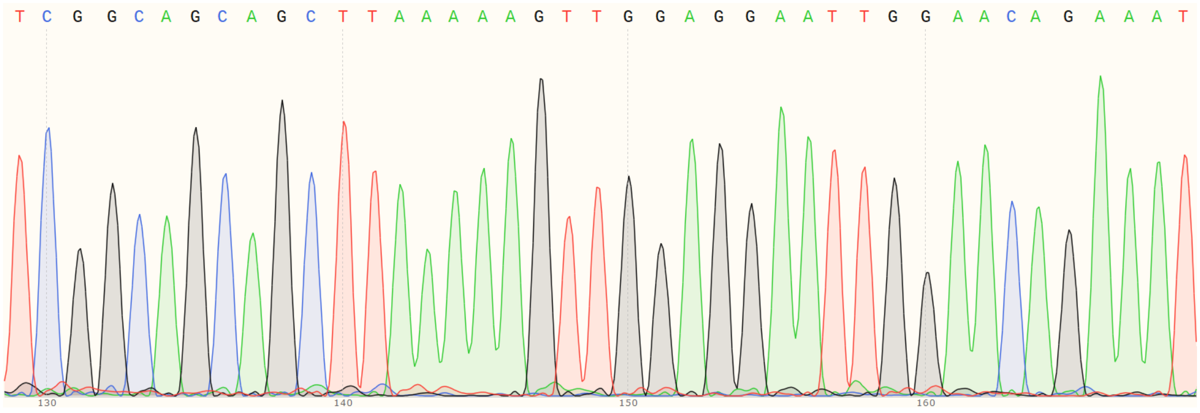
T C G G C A G C A G C T T A A A A A G T T G G A G G A A T T G G A A C A G A A A T
130 140 150 160

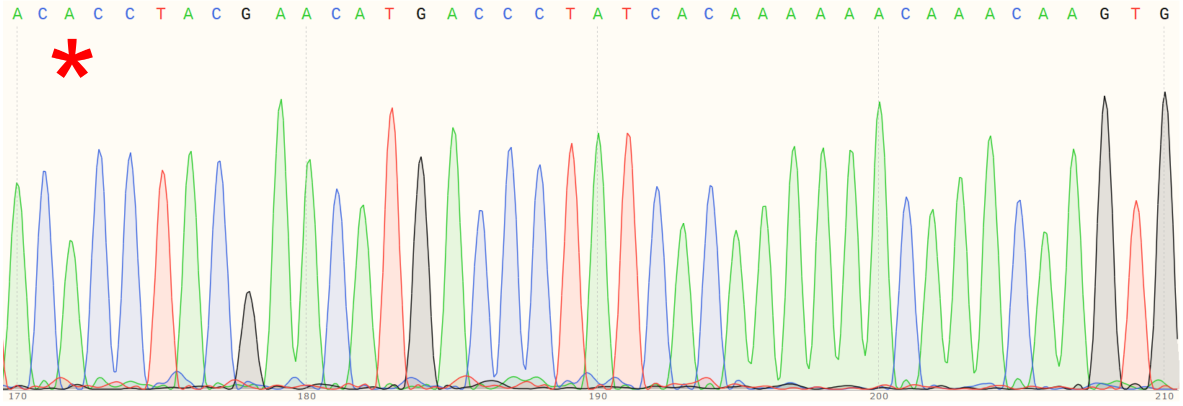
A C A C C T A C G A A C A T G A C C C T A T C A C A A A A A A C A A A C A A G T G
*
170 180 190 200 210

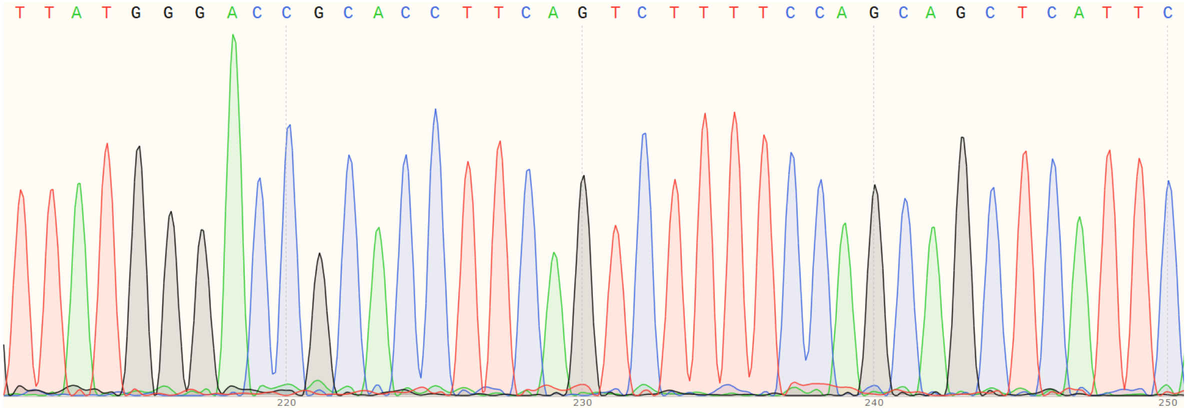
T T A T G G G A C C G C A C C T T C A G T C T T T T C C A G C A G C T C A T T C
220 230 240 250

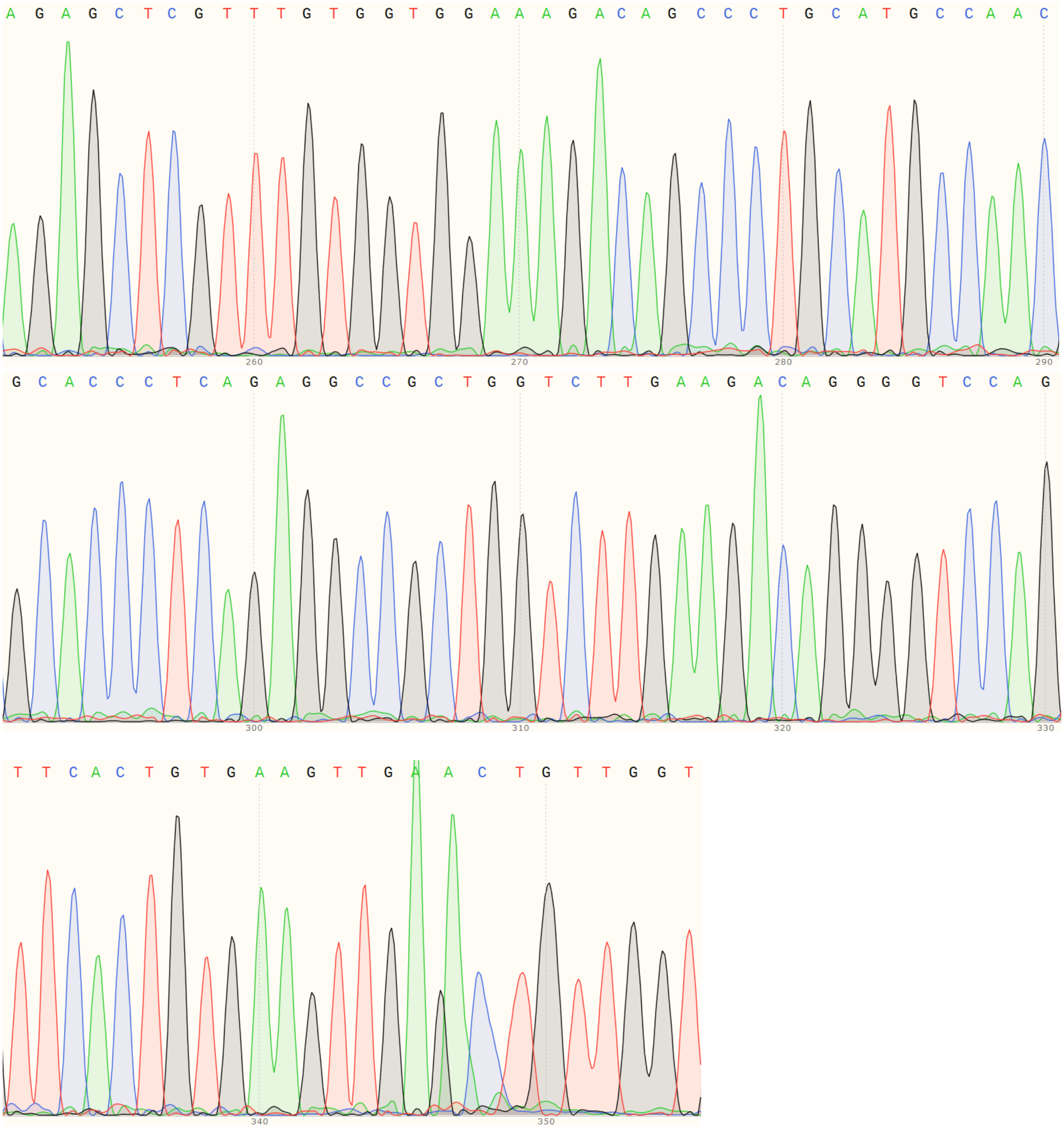
A G A G C T C G T T T G T G G T G G A A A G A C A G C C C T G C A T G C C A A C
260 270 280 290
G C A C C C T C A G A G G C C G C T G G T C T T G A A G A C A G G G G T C C A G
300 310 320 330
T T C A C T G T G A A G T T G A A C T G T T G G T
340 350
